# State-to-State Variation in Opioid Dispensing Changes Following the Release of the 2016 CDC Guideline for Prescribing Opioids for Chronic Pain

**DOI:** 10.1001/jamanetworkopen.2023.32507

**Published:** 2023-09-11

**Authors:** Xiru Lyu, Gery P. Guy, Grant T. Baldwin, Jan L. Losby, Amy S. B. Bohnert, Jason E. Goldstick

**Affiliations:** 1 Injury Prevention Center, University of Michigan, Ann Arbor; 2 Division of Overdose Prevention, Centers for Disease Control and Prevention, Atlanta, Georgia; 3 Department of Anesthesiology, University of Michigan, Ann Arbor; 4 Department of Psychiatry, University of Michigan, Ann Arbor; 5 Department of Emergency Medicine, University of Michigan, Ann Arbor

## Abstract

**Importance:**

Evidence suggests that opioid prescribing was reduced nationally following the 2016 release of the Guideline for Prescribing Opioids for Chronic Pain by the US Centers for Diseases Control and Prevention (CDC). State-to-state variability in postguideline changes has not been quantified and could point to further avenues for reducing opioid-related harms.

**Objective:**

To estimate state-level changes in opioid dispensing following the 2016 CDC Guideline release and explore state-to-state heterogeneity in those changes.

**Design, Setting, and Participants:**

This cross-sectional study included information on opioid prescriptions for US individuals between 2012 and 2018 from an administrative database. Serial cross-sections of monthly opioid dispensing trajectories in each US state and the District of Columbia were analyzed using segmented regression to characterize preguideline dispensing trajectories and to estimate how those trajectories changed following the 2016 guideline release. Data were analyzed January to March 2023.

**Exposure:**

The March 2016 CDC Guideline for Prescribing Opioids for Chronic Pain.

**Main Outcomes and Measures:**

Four measures of opioid dispensing: opioid dispensing rate per 100 000 persons, long-acting opioid dispensing rate per 100 000 persons, high-dose (90 or more morphine milligram equivalents [MME] per day) dispensing rate per 100 000 persons, and average per capita MME. All measures were calculated monthly, from January 2012 through December 2018.

**Results:**

Data from approximately 58 900 retail pharmacies were included in analysis, representing approximately 92% of US retail prescriptions. The overall monthly dispensing rate in the US in early 2012 was approximately 7000 per 100 000 population. Following the 2016 guideline release, the already-decreasing slope accelerated nationally for the overall dispensing rate (preguideline slope, −23.19; postguideline slope, −48.97; change in slope, 25.97 [95% CI, 18.67-32.95]), long-acting dispensing rate (preguideline slope, −1.03; postguideline slope, −5.94; change in slope, 4.90 [95% CI, 4.26-5.55]), high-dose dispensing (preguideline slope, −3.52; postguideline slope, −7.63; change in slope, 4.11 [95% CI, 3.49-4.73]), and per-capita MME (preguideline slope, −0.22; postguideline slope, −0.58; change in slope, 0.36 [95% CI, 0.30-0.42]). For all outcomes, nearly all states showed analogous acceleration of an already-decreasing slope, but there was substantial state-to-state heterogeneity. Slope changes (preguideline − postguideline slope) ranged from 9.15 (Massachusetts) to 74.75 (Mississippi) for overall dispensing, 1.88 (Rhode Island) to 13.41 (Maine) for long-acting dispensing, 0.71 (District of Columbia) to 13.68 (Maine) for high-dose dispensing, and 0.06 (Hawaii) to 0.91 (Arkansas) for per capita MME.

**Conclusions and Relevance:**

The 2016 CDC Guideline release was associated with broad reductions in prescription opioid dispensing, and those changes showed substantial geographic variability. Determining the factors associated with these state-level differences may inform further improvements to ensure safe prescribing practices.

## Key Points

**Question:**

Did changes in opioid prescribing following the US Centers for Disease Control and Prevention (CDC) releasing the 2016 CDC Guideline for Prescribing Opioids for Chronic Pain vary across states?

**Findings:**

In this cross-sectional study, a vast majority of states showed decreasing preguideline trends for overall opioid dispensing, high-dose dispensing, long-acting dispensing, and average morphine milligram equivalents per capita that accelerated following the release of the 2016 CDC Guideline, although there was substantial state-to-state variability in those changes.

**Meaning:**

These results suggest that understanding why some states showed greater changes than others may inform subsequent approaches to safe pain management.

## Teaser

This cross-sectional study of opioid prescription data compares changes in prescription rates by US state following the 2016 release of the US Centers for Disease Control and Prevention Guideline for Prescribing Opioids for Chronic Pain.

## Introduction

In March 2016, the US Centers for Disease Control and Prevention (CDC) published the CDC Guideline for Prescribing Opioids for Chronic Pain (hereafter the 2016 CDC Guideline)^1^ to curb opioid-related harms that have escalated over the past 2 decades. The 2016 CDC Guideline provides evidence-based recommendations on prescribing opioids to patients aged 18 years and older in primary care settings to treat chronic pain, excluding active cancer pain, palliative care, and end-of-life care.^1,2,3^ The goal of this voluntary guideline was to reduce opioid-related harms by enhancing safety of pain care, improving communication between physician and patient on the risks and benefits of opioid therapy and reducing the risks of long-term opioid use (eg, opioid use disorder), while also providing patients with chronic pain needed pain relief.

Prior evaluations of the 2016 CDC Guideline have estimated changes in opioid prescribing following the guideline release. The first evaluation showed accelerations of previously decreasing trends in several opioid prescribing outcomes including overall prescribing, high-dose prescribing, mean morphine milligram equivalents (MME) per capita, and opioid and benzodiazepine coprescribing.^4^ Subsequent evaluations showed opioid prescribing decreased substantially relative to the preguideline trends among certain patient populations with chronic pain,^5^ that rates and characteristics of opioid initiation changed following the 2016 CDC Guideline release,^6^ and that the observed accelerating declines were observed across most specialties.^7^ Other analyses showed accelerating rates of rapid tapering of opioids—treatment not consistent with guideline-concordant care—following the guideline release,^8^ which reflects broader concerns about unintended consequences of the 2016 CDC Guideline and the undertreatment of pain.^9,10^ Emphasizing individualized patient-centered care and shared decision-making is one of many goals of the updated 2022 CDC Clinical Practice Guideline for Prescribing Opioids for Pain.^11,12^ While other related analyses have been conducted—such as analyses showing increased rates of nonopioid prescribing following the 2016 CDC Guideline release^13^—none have examined state-to-state variability in the changes following the 2016 CDC Guideline release, which is the purpose of this report.

There is substantial regional heterogeneity in overdose morbidity and mortality,^14,15,16^ state-to-state variability opioid prescribing rates,^17,18^ and in states’ capacity to deliver medication-assisted opioid use disorder treatment,^19^ all of which suggest possible regional variability in opioid prescribing changes following the 2016 CDC Guideline release. This study analyzes several measures of opioid dispensing rates using an administrative database at the state level, focusing on disaggregating the national trends that have been the focus of all previous 2016 CDC Guideline evaluations and showing state-level trends and changes. We hypothesized that there would be substantial state-to-state heterogeneity in the nationally observed change in prescribing practices following the release of the 2016 CDC Guideline.

## Methods

### Data and Outcomes

We analyzed monthly opioid dispensing data from the Xponent database (IQVIA)^20^ for all 50 states and the District of Columbia (expressed as *51 states* hereafter) between 2012 and 2018. This study uses no human participant data and is exempt from institutional review board review and informed consent requirements per the Common Rule; we followed Strengthening the Reporting of Observational Studies in Epidemiology (STROBE) reporting guideline.

The Xponent administrative database provides weighted projections of outpatient opioid prescriptions from approximately 58 900 US retail pharmacies that dispense approximately 92% of US retail prescriptions. These data have been used in several previous studies of opioid prescribing trajectories.^18,21^ Due to changes in how the number of prescriptions filled are counted after 2018,^22^ this analysis only focused on trends up to 2018. All calculations excluded buprenorphine products intended to treat opioid use disorder. Additionally, the only buprenorphine products left in the data set (Butrans and Belbuca) were also excluded from the analysis of mean MME per capita due to the lack of a well-validated MME conversion factor for those medications. A table with all drugs in the data set that met inclusion criteria, and whether or not the drug is long-acting, is given in eTable 1 in Supplement 1.

We used annual US Census state-level population estimates available through the American Community Survey^23^ to compute per capita prescribing measures. Using those data sources, for each month we computed 4 state-level opioid prescribing outcomes. The first was overall opioid dispensing rate, which is the total number of opioid prescriptions dispensed per 100 000 persons. The second was high-dosage dispensing rate, which is the total number of high-dosage opioid prescriptions dispensed (each 90 MME/d or higher) per 100 000 persons. Third, long-acting (eg, methadone) dispensing rate, which is the total number of long-acting opioid prescriptions dispensed per 100 000 persons. And the fourth was mean MME per capita, calculated by dividing the total MME dispensed by the size of the population. MME was calculated as the product of quantity, strength per unit, and MME conversion factor, summed across each month in each state. As shorthand, we refer to the first 3 outcomes as prescriptions per 100 000 persons throughout the study.

### Statistical Analysis

To quantify changes in prescribing trajectories before vs after the 2016 CDC Guideline release, we used interrupted time series analysis with segmented regressions.^24^ These models essentially fit linear regression models to the trends before and after the guidelines were released, allowing both the level and slope to change after the 2016 CDC Guideline release. Level changes (ie, shifts in the model intercept following the 2016 CDC Guideline release) correspond to an immediate shift in each outcome following the 2016 CDC Guideline release, and slope changes correspond to a change in the rate of change (eg, acceleration of a decreasing trend). The breakpoint in these regressions corresponded to the timing of the 2016 CDC Guideline release—March 2016. Because this voluntary guideline did not have any global implementation we chose not to incorporate an implementation lag into the analysis. We note that this choice, if anything, is conservative because if there truly was an implementation lag, the assumption of an immediate effect would mix the periods immediately prior to and following the release of the guideline, attenuating any estimate of the postguideline change.

The analysis focuses on state-to-state heterogeneity in the primary inferential target: the difference in estimated preguideline and postguideline slopes. Specifically, for a given state, we modeled the month *t* dispensing rate, *Y_t_*, as a linear function of *t* (time in months, starting from January 2012), the slope and intercept of which were allowed to change based on the indicator *G_t_*, for which “1” is if month *t* is after the guideline release and “0” otherwise. The segmented regression equation is:

*Y_t_*_ = _*β*_0 _+ *β*_1_t + *β*_2_*G_t_* + *β_3_*t*G_t_* + ε_t_

where β_2_ quantifies the level shift following the guideline release, and β_3_ (the focal parameter) quantifies the change in slope. An increase in the slope suggests a movement in the trend toward less prescribing, either through reversing an increasing slope or accelerating an already decreasing slope. For example, with a preguideline slope of 1 and a postguideline slope of −1, the slope change was calculated as 2, implying a reversal of the increasing prescribing trend. Similarly, an increase in the magnitude of a decreasing slope—for example, if the preguideline slope was −1 and the postguideline to −3—would be calculated as a positive slope change (2, in this case).

We first descriptively showed changes in the distribution of prescribing levels with histograms of the prescribing outcomes—at the state level—at the first time point (January 2012) and the final time point (December 2018). Then, we fit the segmented regression models to each state and mapped the postguideline slope changes for each outcome, to explore geographic variability in the changes. For each outcome, we also quantified and displayed prescribing changes at the national level using the same data, to provide context for the state-level variability. Estimation uncertainty (eg, for confidence intervals) was quantified using Huber-White robust standard errors, which provides, among other things, inference that is robust for potential temporal autocorrelation. As a sensitivity analysis to evaluate whether the postguideline changes were affected by regression to the mean—ie, if states with higher baseline levels showed greater changes following the 2016 CDC Guideline release—we plotted the prescribing levels at the first time point (January 2012) in each state against the observed slope difference (at the state level) for each outcome and calculated the Pearson correlation. All statistical analyses were performed in R version 4.3.1 (R Project for Statistical Computing), and the package sandwich was utilized for calculating Huber-White robust standard errors. All significance testing was based on 2-sided tests with a threshold of *P* < .05.

## Results

**Figure 1.**
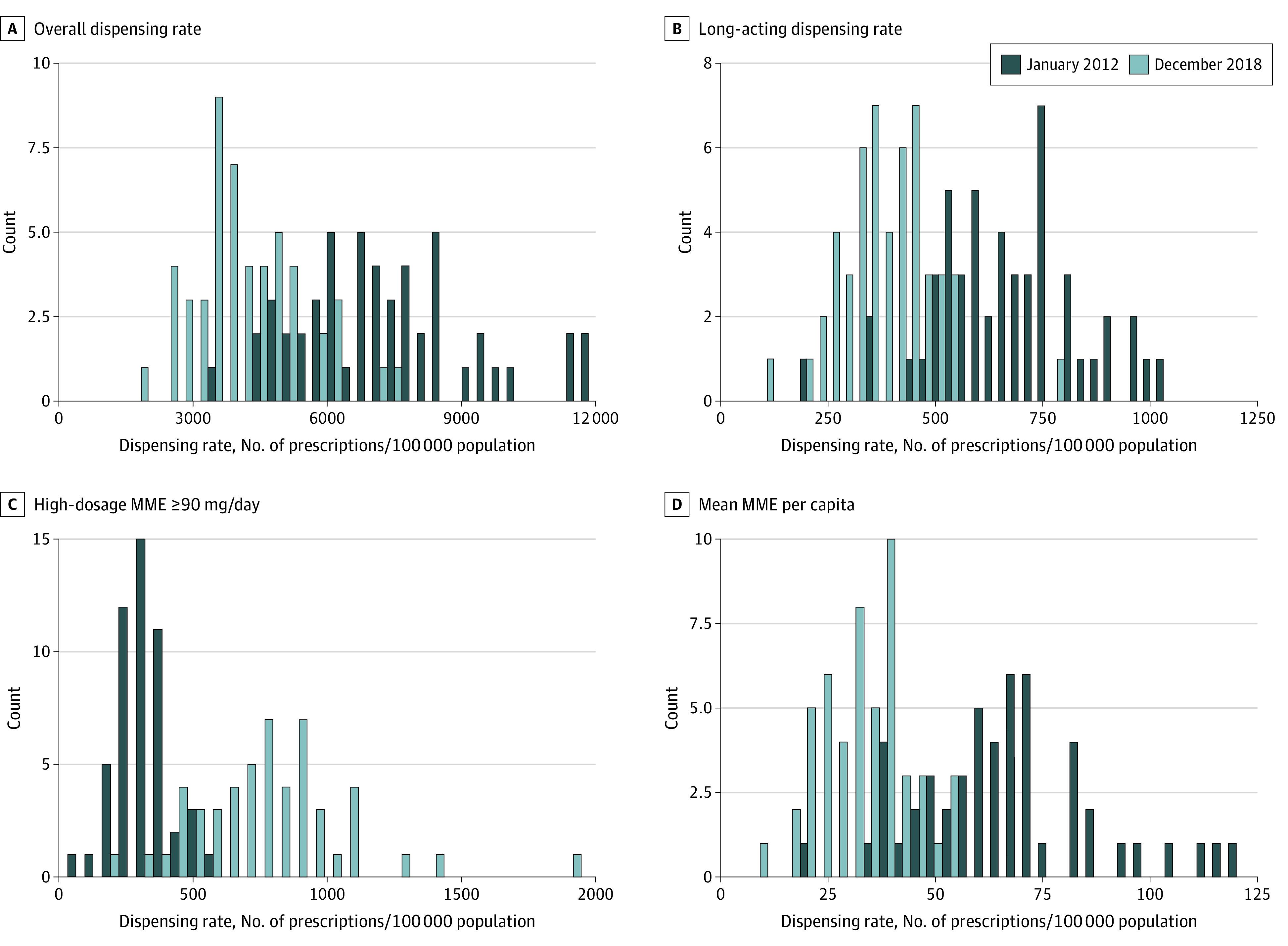
Distributions of Each State-Level Opioid Prescribing Measure in January 2012 and in December 2018 MME indicates morphine milligram equivalents.

Compared with the preguideline period in January 2012, distributions of all 4 outcome measures in December 2018 were more concentrated at lower values, showing general reductions in all 4 prescribing outcomes following the release of the 2016 CDC Guideline (Figure 1). For example, there were 30 states with a mean MME per capita of over 60 in January 2012, and there were zero such states in December 2018. Subsequent analyses focused on changes to the state-level prescribing trajectories, rather than simple pre-post comparisons that could be confounded by preexisting secular trends.

### Overall Dispensing Rate

**Figure 2.**
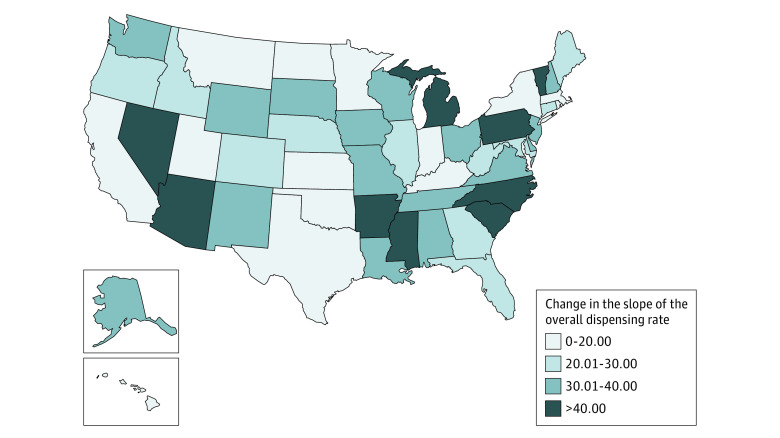
Estimated State-Level Change of the Overall Prescribing Rate Slope Following the 2016 CDC Guideline Release States are colored relative to the magnitude of estimated overall prescribing rate slope changes from the preguideline to the postguideline period. Darker colors correspond to larger changes (preguideline slope – postguideline slope) in the slope. Changes were not statistically significant for Kentucky, Oklahoma, and Texas.

At the national level, starting from an overall monthly dispensing rate of approximately 7000 per 100 000 population in early 2012, the already decreasing trend in the monthly dispensing rate per 100 000 persons more than doubled following the 2016 CDC Guideline release, increasing in magnitude from −23.19 to −48.97 (β_3_ = −25.79; 95% CI, −32.95 to −18.67). Among the 51 states, 48 (94%) had a statistically significant change in the overall dispensing rate slope following the release of 2016 CDC Guideline (Figure 2). Most of these changes corresponded to the acceleration of an already decreasing trend (eFigure 1 in Supplement 1). Particularly, all states in the Northeast and West regions had statistically significant postguideline slope changes in the overall dispensing rate trend, and all states showing nonsignificant reductions were in the South (eTable 2 in Supplement 1). The change of trends was the least for Massachusetts (from a preguideline monthly reduction of 27.25 prescriptions per 100 000 to a postguideline monthly reduction of 36.40 prescriptions per 100 000) and the most for Mississippi (from a monthly decrease of 23.86 prescriptions per 100 000 prior to the guideline to a monthly decrease of 98.61 prescriptions per 100 000 after the guideline release).

### Long-Acting Dispensing Rate

**Figure 3.**
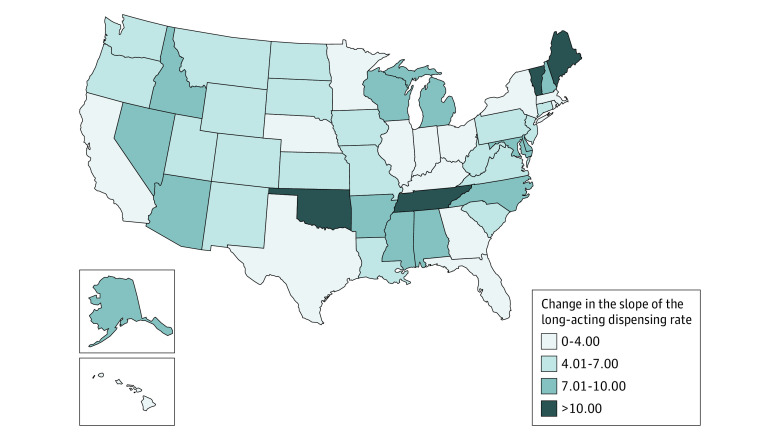
Estimated State-Level Change of the Long-Acting Prescribing Rate Slope Following the 2016 CDC Guideline Release States are colored relative to the magnitude of estimated long-acting dispensing rate slope changes from the preguideline to the postguideline period. Darker colors correspond to larger changes (preguideline slope – postguideline slope) in the slope. Changes were statistically significant for all states.

Nationally, baseline monthly rates of long-acting opioid dispensing in early 2012 were approximately 600 per 100 000 persons. There was a decreasing trend showing a mean monthly reduction of 1.03 long-acting prescriptions per 100 000 persons prior to the guideline release; that declining rate increased more than 5-fold to 5.94 high-dose prescriptions per 100 000 persons following the 2016 CDC Guideline release (β_3_ = −4.90; 95% CI, −5.55 to −4.26). Following the 2016 CDC Guideline release, all 51 states had a statistically significant reduction in the dispensing rate slope for long-acting prescriptions (Figure 3). Most of these changes were accelerations of previously existing decreasing trends (eFigure 2 in Supplement 1). The 4 states with the largest slope changes were in the South and Northeast regions (eTable 2 in Supplement 1). Moreover, the slope change was estimated to be the least in Rhode Island, with the preguideline monthly decrease of 3.27 long-acting prescriptions per 100 000 persons further accelerated to a monthly decrease of 5.14 prescriptions per 100 000 postguideline. The greatest change happened in Maine, whose nearly flat preguideline slope shifted to a decrease of 13.18 prescriptions 100 000 postguideline.

### High-Dose Dispensing Rate

**Figure 4.**
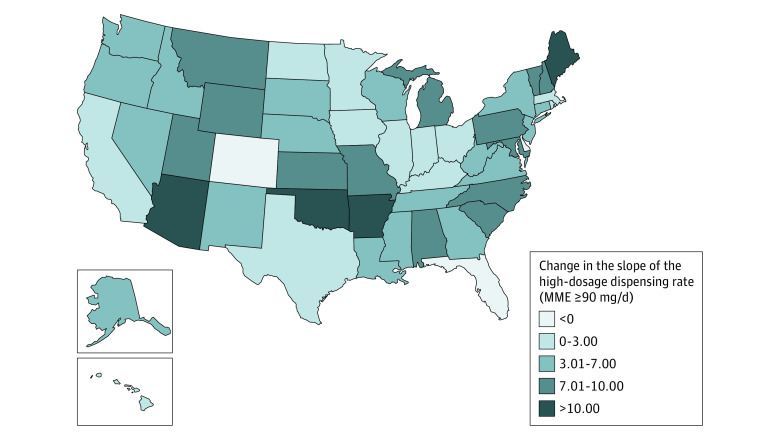
Estimated State-Level Change of the High-Dosage Prescribing Rate Slope Following the 2016 CDC Guideline Release High dosage was defined as a prescription for an opioid of 90 morphine milligram equivalents per day. States are colored relative to the magnitude of estimated high-dosage dispensing rate changes from the preguideline to the postguideline period. Darker colors correspond to larger changes (preguideline slope – postguideline slope) in the slope. Changes were not statistically significant for Colorado, Delaware, and Massachusetts.

There was a national baseline of approximately 700 high-dose prescriptions each month per 100 000 population. Prior to the 2016 CDC Guideline release there was a national average decrease of 3.52 high-dose prescriptions per 100 000 population, and that rate of decrease more than doubled, to 7.63, following the guideline release (β_3_ = −4.11; 95% CI, −4.73 to −3.49). A large majority of states (48 of 51 [94%]) had a statistically significant change in the slope of the high-dosage (90 MME/d or higher) dispensing rate (Figure 4). Florida was the only state with a negative preguideline slope that showed a statistically significant shallowing (ie, decrease in magnitude) during the postguideline period (ie, prescribing rates were declining at a slower rate following the 2016 CDC Guideline release); following the 2016 CDC Guideline release, all other states had either an acceleration of the preguideline decreasing trend or a reversal of the increasing trend (eFigure 3 in Supplement 1). Additionally, the Midwest was the only region showing statistically significant postguideline slope changes in all states (eTable 2 in Supplement 1), with magnitudes ranging from 1.11 to 7.94 (eFigure 3 in Supplement 1). Of note, the trend difference was the smallest for DC, where the preguideline decreasing slope of 1.04 prescriptions per 100 000 per month changed to a decreasing slope of 1.76 prescriptions 100 000 per month in the postguideline period. The largest change was seen in Maine, where the monthly decrease of 2.83 high-dosage prescriptions per 100 000 persons further changed more than 6-fold to a 16.51-monthly decrease.

### Mean MME per Capita

**Figure 5.**
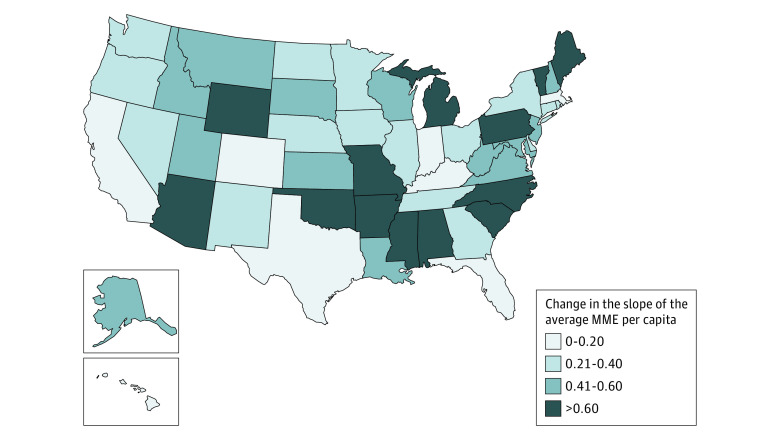
Estimated State-Level Change of the Mean Morphine Milligram Equivalents (MME) per Capita Slope Following the 2016 CDC Guideline Release States are colored relative to the magnitude of estimated mean MME per capita slope changes from the preguideline to the postguideline period. Darker colors correspond to larger changes (preguideline slope – postguideline slope) in the slope. Changes were not statistically significant for Florida.

The mean per capita MME dispensed in early 2012 was approximately 62.2. Similar to the other outcomes, mean MME per capita was decreasing nationally prior to the guideline release (0.22 MME per capita per month), and that rate of decrease more than doubled (0.58 MME per capita per month) following the guideline release (β_3_ = −0.36; 95% CI, −0.42 to −0.30). There were 50 states (98%) with a statistically significant reduction in the mean MME per capita slope following the 2016 CDC Guideline release (Figure 5). As with the other outcomes, most of these changes reflected an acceleration of preexisting decreasing trends (eFigure 4 in Supplement 1). Of note, all states in the Northeast, Midwest, and West regions observed statistically significant slope changes toward reduced dispensing (eTable 2 in Supplement 1). Additionally, the smallest slope change was seen in Hawaii (the monthly slope changed from −0.28 preguideline to −0.35 postguideline), while the largest change was in Arkansas, where a nearly flat preguideline slope shifted to a decreasing slope of 0.86 MME per capita per month.

### Baseline Dispensing Rates and Postguideline Changes

In eFigure 5 in Supplement 1 we show scatterplots of the dispensing rates in each state in January 2012 vs the postguideline slope change. We observed weak Pearson correlations for the overall dispensing rate (*R^2^* = 0.08), high-dose dispensing rate (*R^2^* = 0.03), and the mean MME per capita (*R^2^* = 0.05), suggesting that the baseline dispensing rate was negligibly associated with the postguideline slope change for those outcomes. For long-acting dispensing there was a larger effect size for the association (*R^2^* = 0.36), showing that states with larger dispensing rates in January 2012 tended to have larger postguideline slope changes.

## Discussion

Our study examined geographic differences in opioid prescribing practice changes following the release of the CDC 2016 Guideline for Prescribing Opioids for Chronic Pain. There is previous evidence that, while dispensing rates were already decreasing, the release of the 2016 CDC Guideline corresponded to an acceleration of those decreasing trends,^4,6^ and our results largely corroborate those findings. Across four different dispensing measures, we found broad acceleration of previously existing decreases in dispensing, though magnitude of these changes varied geographically. Specifically, we found changes corresponding to greater decreases universally with regard to long-acting prescribing. Most states (ie, more than 90%) observed changes toward reduced dispensing (eg, an acceleration of an already decreasing slope) in the other 3 outcomes. At the same time, changes corresponding to a shallowing of the decreasing slope were rare. The only example was Florida, which showed a shallowing of the decreasing slope in the high-dose dispensing rate following the 2016 CDC Guideline release. These findings provide further evidence that the 2016 CDC Guideline release coincided to decreases in opioid dispensing, although other cooccurring changes may explain the state-to-state differences.

Although the 2016 CDC Guideline may have helped to catalyze other secular changes in the opioid policy landscape, there remains great state-to-state heterogeneity, which may explain some of the geographic trends found here. There is documented state-to-state variability in the existence of laws governing opioid prescribing and related factors, including: continuing medical education requirements, pain management clinic policies, laws restricting or prohibiting multiple prescribers, and drug supply management policies.^25^ At the same time, by December 2021, all 50 states and the District of Columbia have implemented prescription drug monitoring programs.^26^ Therefore, despite state-to-state heterogeneity in the opioid policy landscape, the consistent opioid prescribing practice changes across states found in this report suggested many common threads as well.

Other state-level factors that may relate to variability in opioid-related outcomes at the state level include pre-2016 CDC Guideline dispensing trends and opioid overdose mortality rates. Indeed, sensitivity analyses showed that baseline (January 2012) rates of long-acting dispensing were correlated with postguideline changes in long-acting prescribing trajectories, though preguideline rates of dispensing were very weakly correlated with postguideline changes in dispensing for the other 3 outcomes. Regarding opioid overdose mortality, within the 50 states and the District of Columbia, the age-adjusted opioid overdose death rate per 100 000 persons varied from 5.3 (Nebraska) to 70.0 (West Virginia)^27^ in 2020. Further research is required to determine whether variability in overdose mortality and other opioid-related harms (eg, treatment admissions for opioid use disorder) create differential reactivity to external factors that may change prescribing, such as the 2016 CDC Guideline.

### Limitations

This study had several limitations. First, the opioid prescribing policy landscape has evolved considerably since the CDC 2016 Guideline release,^28^ including major initiatives such as the 21st Century Cares Act and universal implementation of prescription drug monitoring programs across the country, and so trend changes in opioid prescribing practices may not be solely attributed to the 2016 CDC Guideline. However, it stands to reason that the 2016 CDC Guideline release may have helped to catalyze adjustments in prescription practices, making those types of secular changes and their effects part and parcel of the 2016 CDC Guideline effect. Regardless, the focus of this work was on describing state-to-state heterogeneity in previously identified changes in prescription practices following the 2016 CDC Guideline release, and less on causal attribution. Second, the Xponent administrative database has no patient-level demographic information. Therefore, beyond geographic trends, this data set cannot permit further analysis on demographic variations in opioid prescribing practices. In addition, given changes to the data, analyses could only be conducted through 2018. Lastly, this study focused on dispensing data from retail pharmacies, and as a result it provided no insights on illicit opioid use, an issue that also plays an important role in the opioid overdose epidemic.

## Conclusions

This study demonstrated that, while the release of CDC 2016 Guideline was associated with nearly universal reductions in opioid prescribing, there were still notable geographic differences in these changes. These findings highlight state-level trends and variation in prescribing practices over time, which may inform efforts to improve prescribing practices and ultimately reduce opioid-related harms.

The 2022 CDC Clinical Practice Guideline to Prescribing Opioids for Pain^29^ updates the 2016 CDC Guideline and the new guideline supports flexible, tailored, patient-centered care to help clinicians work with their patients to provide safe and effective pain care. Future research should evaluate changes to pain care following its release.

## Notes

Supplement 1. **eTable 1.** Description of Product Name, Generic Drug Name, and Whether or Not the Drugs Are Long-Acting for Each Drug Identified in the IQVIA Data Set That Contributed to the Overall Dispensing Totals **eTable 2.** Changes in Slope (Pre- vs. Post-Guideline) by Census Region for Each of the Four Primary Outcomes **eFigure 1.** Trends in the Overall Dispensing Rate From 2012 Through 2018, With Linear Regression Lines Showing the Pre- and Post-Guideline Slopes Superimposed **eFigure 2.** Trends in the Long-Acting Dispensing Rate From 2012 Through 2018, With Linear Regression Lines Showing the Pre- and Post-Guideline Slopes Superimposed **eFigure 3.** Trends in the High-Dose (≥90 MME/Day) Dispensing Rate From 2012 Through 2018, With Linear Regression Lines Showing the Pre- and Post-Guideline Slopes Superimposed **eFigure 4.** Trends in the Per Capita MME Dispensing From 2012 Through 2018, With Linear Regression Lines Showing the Pre- and Post-Guideline Slopes Superimposed **eFigure 5.** Association Between Baseline Dispensing Levels and the Slope Change (Pre- vs Post-Guideline) at the State-Level for Each of the Four Primary Outcomes

Supplement 2.
Data Sharing Statement
