# Supplementary material for: State-to-State Variation in Opioid Dispensing Changes Following the Release of the 2016 CDC Guideline for Prescribing Opioids for Chronic Pain
Source: JAMA Netw Open. 2023 Sep 11;6(9):e2332507. doi: 10.1001/jamanetworkopen.2023.32507 (PMC10495870; doi:10.1001/jamanetworkopen.2023.32507)
Supplement: Supplement 1. — eTable 1. Description of Product Name, Generic Drug Name, and Whether or Not the Drugs Are Long-Acting for Each Drug Identified in the IQVIA Data Set That Contributed to the Overall Dispensing Totals eTable 2. Changes in Slope (Pre- vs. Post-Guideline) by Census Region for Each of the Four Primary Outcomes eFigure 1. Trends in the Overall Dispensing Rate From 2012 Through 2018, With Linear Regression Lines Showing the Pre- and Post-Guideline Slopes Superimposed eFigure 2. Trends in the Long-Acting Dispensing Rate From 2012 Through 2018, With Linear Regression Lines Showing the Pre- and Post-Guideline Slopes Superimposed eFigure 3. Trends in the High-Dose (≥90 MME/Day) Dispensing Rate From 2012 Through 2018, With Linear Regression Lines Showing the Pre- and Post-Guideline Slopes Superimposed eFigure 4. Trends in the Per Capita MME Dispensing From 2012 Through 2018, With Linear Regression Lines Showing the Pre- and Post-Guideline Slopes Superimposed eFigure 5. Association Between Baseline Dispensing Levels and the Slope Change (Pre- vs Post-Guideline) at the State-Level for Each of the Four Primary Outcomes [file jamanetwopen-e2332507-s001.pdf]

Supplemental Online Content

Lyu X, Guy G, Baldwin G, Losby JL, Bohnert ASB, Goldstick JE. State-to-state variation in opioid dispensing changes following the release of the 2016 CDC Guideline for Prescribing Opioids for Chronic Pain. *JAMA Netw Open.* 2023;6(9):e2332507. doi:10.1001/jamanetworkopen.2023.32507

eTable 1. Description of Product Name, Generic Drug Name, and Whether or Not the Drugs Are Long-Acting for Each Drug Identified in the IQVIA Data Set That Contributed to the Overall Dispensing Totals

eTable 2. Changes in Slope (Pre- vs. Post-Guideline) by Census Region for Each of the Four Primary Outcomes

eFigure 1. Trends in the Overall Dispensing Rate From 2012 Through 2018, With Linear Regression Lines Showing the Pre- and Post-Guideline Slopes Superimposed

eFigure 2. Trends in the Long-Acting Dispensing Rate From 2012 Through 2018, With Linear Regression Lines Showing the Pre- and Post-Guideline Slopes Superimposed

eFigure 3. Trends in the High-Dose (≥90 MME/Day) Dispensing Rate From 2012 Through 2018, With Linear Regression Lines Showing the Pre- and Post-Guideline Slopes Superimposed

eFigure 4. Trends in the Per Capita MME Dispensing From 2012 Through 2018, With Linear Regression Lines Showing the Pre- and Post-Guideline Slopes Superimposed

eFigure 5. Association Between Baseline Dispensing Levels and the Slope Change (Pre- vs Post-Guideline) at the State-Level for Each of the Four Primary Outcomes

This supplemental material has been provided by the authors to give readers additional information about their work.

© 2023 Lyu X et al. *JAMA Network Open*.

eTable 1. Description of product name, generic drug name, and whether or not the drugs are long-acting for each drug identified in the IQVIA data set that contributed to the overall dispensing totals

PRODUCT NAME	Generic Drug Name	Drug	LongShortActing
AVINZA	Morphine Sulfate	Morphine LA	LA
METHADONE HCL INTENSOL	Methadone Hydrochloride	Methadone	LA
METHADONE HCL	Methadone Hydrochloride	Methadone	LA
ULTRAM ER	Tramadol Hydrochloride	Tramadol	LA
MORPHINE SULFATE	Morphine Sulfate	Morphine LA	LA
FENTANYL	Fentanyl	Fentanyl LA	LA
METHADOSE	Methadone Hydrochloride	Methadone	LA
METHADONE HYDROCHLORIDE	Methadone Hydrochloride	Methadone	LA
FENTANYL TRANSDERMAL SYSTEM	Fentanyl	Fentanyl LA	LA
TRAMADOL HYDROCHLORIDE	Tramadol Hydrochloride	Tramadol	LA
EXALGO	Hydromorphone Hydrochloride	Hydromorphone	LA
KADIAN	Morphine Sulfate	Morphine LA	LA
DURAGESIC	Fentanyl	Fentanyl LA	LA
NUCYNTA ER	Tapentadol Hydrochloride	Tapentadol	LA
OXYCONTIN	Oxycodone Hydrochloride	Oxycodone LA	LA
MS CONTIN	Morphine Sulfate	Morphine LA	LA
RYZOLT	Tramadol Hydrochloride	Tramadol	LA
BUTRANS	Buprenorphine	Buprenorphine	LA
OPANA ER	Oxymorphone Hydrochloride	Oxymorphone LA	LA
ACETAMINOPHEN W/CODEINE	Acetaminophen/Codeine Phosphate	Codeine	SA
ACETAMINOPHEN/CODEINE	Acetaminophen/Codeine Phosphate	Codeine	SA
TRAMADOL HCL	Tramadol Hydrochloride	Tramadol	SA
ACETAMINOPHEN AND CODEINE PHOSPHATE	Acetaminophen/Codeine Phosphate	Codeine	SA
BUTALBITAL-ACETAMINOPHEN-CAFFEINE-CODEINE PHOSPHAT	APAP/Butalbital/Caff/Codeine Phos	Codeine	SA
BUTORPHANOL TARTRATE	Butorphanol Tartrate	Butorphanol	SA
BUTALBITAL-ASPIRIN-CAFFEINE-CODEINE	Aspirin/Butalbital/Caffeine/Codeine Phosphate	Codeine	SA
PENTAZOCINE HCL-NALOXONE HCL	Naloxone Hydrochloride/Pentazocine Hydrochloride	Pentazocine	SA
PENTAZOCINE HCL-ACETAMINOPHEN	Acetaminophen/Pentazocine Hydrochloride	Pentazocine	SA
BUTALBITAL-ACETAMINOPHEN-CAFFEINE-CODEINE	APAP/Butalbital/Caff/Codeine Phos	Codeine	SA
TRAMADOL HYDROCHLORIDE	Tramadol Hydrochloride	Tramadol	SA
TYLENOL WITH CODEINE NO. 3	Acetaminophen/Codeine Phosphate	Codeine	SA
TYLENOL WITH CODEINE NO. 4	Acetaminophen/Codeine Phosphate	Codeine	SA
ULTRAM	Tramadol Hydrochloride	Tramadol	SA
ASCOMP W/CODEINE	Aspirin/Butalbital/Caffeine/Codeine Phosphate	Codeine	SA
RYBIX ODT	Tramadol Hydrochloride	Tramadol	SA
ABSTRAL	Fentanyl Citrate	Fentanyl SA	SA

© 2023 Lyu X et al. *JAMA Network Open*.

ACETAMINOPHEN-HYDROCODONE BITARTRATE	Acetaminophen/Hydrocodone Bitartrate	Hydrocodone	SA
ACETAMINOPHEN-HYDROCODONE BITARTRATE	Acetaminophen/Hydrocodone Bitartr	Hydrocodone	SA
ACETAMINOPHEN-OXYCODONE	Acetaminophen/Oxycodone Hydrochloride	Oxycodone SA	SA
HYDROMORPHONE HCL	Hydromorphone Hydrochloride	Hydromorphone	SA
HYDROMORPHONE HCL/SODIUM CHLORIDE	Hydromorphone Hydrochloride/Sodium Chloride	Hydromorphone	SA
HYDROMORPHONE HCL-SODIUM CHLORIDE	Hydromorphone Hydrochloride/Sodium Chloride	Hydromorphone	SA
HYDROMORPHONE HYDROCHLORIDE	Hydromorphone Hydrochloride	Hydromorphone	SA
MORPHINE SULFATE	Morphine Sulfate	Morphine SA	SA
CODEINE SULFATE	Codeine Sulfate	Codeine	SA
OXYMORPHONE HYDROCHLORIDE	Oxymorphone Hydrochloride	Oxymorphone SA	SA
OXYCODONE-ACETAMINOPHEN	Acetaminophen/Oxycodone Hydrochloride	Oxycodone SA	SA
ROXICET	Acetaminophen/Oxycodone Hydrochloride	Oxycodone SA	SA
MEPERIDINE HCL	Meperidine Hydrochloride	Meperidine	SA
VICODIN	Acetaminophen/Hydrocodone Bitartrate	Hydrocodone	SA
VICODIN ES	Acetaminophen/Hydrocodone Bitartrate	Hydrocodone	SA
HYDROCODONE BITARTRATE-IBUPROFEN	Hydrocodone Bitartrate/Ibuprofen	Hydrocodone	SA
ORAL TRANSMUCOSAL FENTANYL CITRATE	Fentanyl Citrate	Fentanyl SA	SA
OXYCODONE HYDROCHLORIDE	Oxycodone Hydrochloride	Oxycodone SA	SA
OXYCODONE HCL-IBUPROFEN	Ibuprofen/Oxycodone Hydrochloride	Oxycodone SA	SA
OXYCODONE HCL-ASPIRIN	Aspirin/Oxycodone Hydrochloride	Oxycodone SA	SA
OXYCODONE AND ACETAMINOPHEN	Acetaminophen/Oxycodone Hydrochloride	Oxycodone SA	SA
HYDROCODONE BITARTRATE-ACETAMINOPHEN	Acetaminophen/Hydrocodone Bitartrate	Hydrocodone	SA
OXYCODONE HCL-ACETAMINOPHEN	Acetaminophen/Oxycodone Hydrochloride	Oxycodone SA	SA
OXYCODONE HCL	Oxycodone Hydrochloride	Oxycodone SA	SA
FENTANYL CITRATE	Fentanyl Citrate	Fentanyl SA	SA
OXYCODONE-ASPIRIN	ASA/Oxycodone HCl/Oxycodone Terephthalate	Oxycodone SA	SA
MEPERITAB	Meperidine Hydrochloride	Meperidine	SA
TYLOX	Acetaminophen/Oxycodone Hydrochloride	Oxycodone SA	SA
NUCYNTA	Tapentadol Hydrochloride	Tapentadol	SA
LORTAB 5/500	Acetaminophen/Hydrocodone Bitartrate	Hydrocodone	SA
LORTAB 10/500	Acetaminophen/Hydrocodone Bitartrate	Hydrocodone	SA
NORCO	Acetaminophen/Hydrocodone Bitartrate	Hydrocodone	SA

© 2023 Lyu X et al. *JAMA Network Open*.

DILAUDID	Hydromorphone Hydrochloride	Hydromorphone	SA
ENDODAN	Aspirin/Oxycodone Hydrochloride	Oxycodone SA	SA
ENDOCET	Acetaminophen/Oxycodone Hydrochloride	Oxycodone SA	SA
DURAMORPH	Morphine Sulfate	Morphine SA	SA
FENTORA	Fentanyl Citrate	Fentanyl SA	SA
PERCODAN	Aspirin/Oxycodone Hydrochloride	Oxycodone SA	SA
OPANA	Oxymorphone Hydrochloride	Oxymorphone SA	SA
PERCOCET	Acetaminophen/Oxycodone Hydrochloride	Oxycodone SA	SA
ZYDONE	Acetaminophen/Hydrocodone Bitartrate	Hydrocodone	SA
REPREXAIN	Hydrocodone Bitartrate/Ibuprofen	Hydrocodone	SA
ROXICODONE	Oxycodone Hydrochloride	Oxycodone SA	SA
OXYCODONE HYDROCHLORIDE	Oxycodone Hydrochloride	Oxycodone LA	LA
HYDROMORPHONE HYDROCHLORIDE-SODIUM CHLORIDE	Hydromorphone Hydrochloride/Sodium Chloride	Hydromorphone	SA
HYDROSTAT IR	Hydromorphone Hydrochloride	Hydromorphone	SA
HY-PHEN	Acetaminophen/Hydrocodone Bitartrate	Hydrocodone	SA
IBUDONE	Hydrocodone Bitartrate/Ibuprofen	Hydrocodone	SA
INFUMORPH	Morphine Sulfate	Morphine SA	SA
INFUMORPH 200	Morphine Sulfate	Morphine SA	SA
INFUMORPH 500	Morphine Sulfate	Morphine SA	SA
LAZANDA	Fentanyl Citrate	Fentanyl SA	SA
LIQUICET	Acetaminophen/Hydrocodone Bitartrate	Hydrocodone	SA
LORCET	Acetaminophen/Hydrocodone Bitartrate	Hydrocodone	SA
LORCET	Acetaminophen/Hydrocodone Bitartr	Hydrocodone	SA
LORCET 10/650	Acetaminophen/Hydrocodone Bitartrate	Hydrocodone	SA
VICODIN HP	Acetaminophen/Hydrocodone Bitartrate	Hydrocodone	SA
CO-GESIC	Acetaminophen/Hydrocodone Bitartrate	Hydrocodone	SA
OXYMORPHONE HYDROCHLORIDE	Oxymorphone Hydrochloride	Oxymorphone LA	LA
FIORICET WITH CODEINE	APAP/Butalbital/Caff/Codeine Phos	Codeine	SA
SYNALGOS-DC	Aspirin/Caffeine/Dihydrocodeine Bitartrate	Dihydrocodeine	SA
FIORINAL W/CODEINE	Aspirin/Butalbital/Caffeine/Codeine Phosphate	Codeine	SA
ACTIQ	Fentanyl Citrate	Fentanyl SA	SA
LORCET HD	Acetaminophen/Hydrocodone Bitartrate	Hydrocodone	SA
LORCET HD	Acetaminophen/Hydrocodone Bitartr	Hydrocodone	SA
LORCET PLUS	Acetaminophen/Hydrocodone Bitartrate	Hydrocodone	SA
LORCET PLUS	Acetaminophen/Hydrocodone Bitartr	Hydrocodone	SA
LORCET-HD	Acetaminophen/Hydrocodone Bitartrate	Hydrocodone	SA

© 2023 Lyu X et al. *JAMA Network Open*.

LORTAB	Acetaminophen/Hydrocodone Bitartrate	Hydrocodone	SA
LORTAB 10/325	Acetaminophen/Hydrocodone Bitartrate	Hydrocodone	SA
LORTAB 2.5/500	Acetaminophen/Hydrocodone Bitartrate	Hydrocodone	SA
LORTAB 5/325	Acetaminophen/Hydrocodone Bitartrate	Hydrocodone	SA
LORTAB 7	Acetaminophen/Hydrocodone Bitartrate	Hydrocodone	SA
LORTAB 7.5/325	Acetaminophen/Hydrocodone Bitartrate	Hydrocodone	SA
LORTAB 7.5/500	Acetaminophen/Hydrocodone Bitartrate	Hydrocodone	SA
LORTAB ASA	Aspirin/Hydrocodone Bitartrate	Hydrocodone	SA
LORTAB LIQUID	Acetaminophen/Hydrocodone Bitartrate	Hydrocodone	SA
MAGNACET	Acetaminophen/Oxycodone Hydrochloride	Oxycodone SA	SA
MARGESIC-H	Acetaminophen/Hydrocodone Bitartrate	Hydrocodone	SA
MAXIDONE	Acetaminophen/Hydrocodone Bitartrate	Hydrocodone	SA
MEDIPAIN 5	Acetaminophen/Hydrocodone Bitartrate	Hydrocodone	SA
MEGAGESIC	Acetaminophen/Hydrocodone Bitartrate	Hydrocodone	SA
MEGAMOR	Acetaminophen/Hydrocodone Bitartrate	Hydrocodone	SA
MEPEREDINE	Meperidine Hydrochloride	Meperidine	SA
MEPERIDINE	Meperidine Hydrochloride	Meperidine	SA
MEPERIDINE HYDROCHLORIDE	Meperidine Hydrochloride	Meperidine	SA
METHADONE HCL CONCENTRATE	Methadone Hydrochloride	Methadone	LA
MORPHINE	Morphine Sulfate	Morphine SA	SA
MORPHINE SULF	Morphine Sulfate	Morphine SA	SA
VICOPROFEN	Hydrocodone Bitartrate/Ibuprofen	Hydrocodone	SA
ANEXSIA	Acetaminophen/Hydrocodone Bitartrate	Hydrocodone	SA
ACETAMINOPHEN, CAFFEINE, AND DIHYDROCODEINE	Acetaminophen/Caffeine/Dihydrocodeine Bitartrate	Dihydrocodeine	SA
TREZIX	Acetaminophen/Caffeine/Dihydrocodeine Bitartrate	Dihydrocodeine	SA
LEVORPHANOL TARTRATE	Levorphanol Tartrate	Levorphanol	LA
CARISOPRODOL/ASPIRIN/CODEINE	Aspirin/Carisoprodol/Codeine Phosphate	Codeine	SA
SUBSYS	Fentanyl	Fentanyl SA	SA
STAGESIC	Acetaminophen/Hydrocodone Bitartrate	Hydrocodone	SA
XODOL 7.5/300	Acetaminophen/Hydrocodone Bitartrate	Hydrocodone	SA
XOLOX	Acetaminophen/Oxycodone Hydrochloride	Oxycodone SA	SA
XODOL	Acetaminophen/Hydrocodone Bitartrate	Hydrocodone	SA
XODOL 5/300	Acetaminophen/Hydrocodone Bitartrate	Hydrocodone	SA
DOLOPHINE HCL	Methadone Hydrochloride	Methadone	LA
CONZIP	Tramadol Hydrochloride	Tramadol	LA

© 2023 Lyu X et al. *JAMA Network Open*.

MORPHINE SULFATE IMMEDIATE RELEASE	Morphine Sulfate	Morphine SA	SA
MORPHINE SULFATE IR	Morphine Sulfate	Morphine SA	SA
MS/L	Morphine Sulfate	Morphine SA	SA
MS/L CONCENTRATE	Morphine Sulfate	Morphine SA	SA
MS/S	Morphine Sulfate	Morphine SA	SA
MSIR	Morphine Sulfate	Morphine SA	SA
NARVOX	Acetaminophen/Oxycodone Hydrochloride	Oxycodone SA	SA
NORCET	Acetaminophen/Hydrocodone Bitartrate	Hydrocodone	SA
NORCET-7.5	Acetaminophen/Hydrocodone Bitartrate	Hydrocodone	SA
PRIMLEV	Acetaminophen/Oxycodone Hydrochloride	Oxycodone SA	SA
NOVAPLUS FENTANYL	Fentanyl	Fentanyl LA	LA
NUMORPHAN HCL	Oxymorphone Hydrochloride	Oxymorphone SA	SA
OMS	Morphine Sulfate	Morphine SA	SA
ONCET	Acetaminophen/Hydrocodone Bitartrate	Hydrocodone	SA
ONSOLIS	Fentanyl Citrate	Fentanyl SA	SA
OXECTA	Oxycodone Hydrochloride	Oxycodone SA	SA
OXY IR	Oxycodone Hydrochloride	Oxycodone SA	SA
OXYCET	Acetaminophen/Oxycodone Hydrochloride	Oxycodone SA	SA
ZAMICET	Acetaminophen/Hydrocodone Bitartrate	Hydrocodone	SA
ACETAMINOPHEN/CODEINE #2	Acetaminophen/Codeine Phosphate	Codeine	SA
ACETAMINOPHEN/CODEINE #3	Acetaminophen/Codeine Phosphate	Codeine	SA
ACETAMINOPHEN/CODEINE #4	Acetaminophen/Codeine Phosphate	Codeine	SA
ACETAMINOPHEN/CODEINE PHOSPHATE	Acetaminophen/Codeine Phosphate	Codeine	SA
ACETAMINOPHEN/PENTAZOCINE HCL	Acetaminophen/Pentazocine Hydrochloride	Pentazocine	SA
ACETAMINOPHEN/PROPOXYPHENE	Acetaminophen/Propoxyphene Hydrochloride	Propoxyphene	SA
ACETAMINOPHEN/PROPOXYPHENE HCL	Acetaminophen/Propoxyphene Hydrochloride	Propoxyphene	SA
ACETAMINOPHEN/PROPOXYPHENE NAPSYLATE	Acetaminophen/Propoxyphene Napsylate	Propoxyphene	SA
ACETAMINOPHEN-CODEINE PHOSPHATE	Acetaminophen/Codeine Phosphate	Codeine	SA
ACETATAB	Acetaminophen/Codeine Phosphate	Codeine	SA
APAP AND CODEINE	Acetaminophen/Codeine Phosphate	Codeine	SA
APAP W/ CODEINE	Acetaminophen/Codeine Phosphate	Codeine	SA
OXYCODONE HCL AND ACETAMINOPHEN	Acetaminophen/Oxycodone Hydrochloride	Oxycodone SA	SA
APAP W/CODEINE	Acetaminophen/Codeine Phosphate	Codeine	SA
APAP W/PROPOXYPHENE NAPSYLATE	Acetaminophen/Propoxyphene Napsylate	Propoxyphene	SA
DEPODUR	Morphine Sulfate Liposome	Morphine LA	LA
DISKETS DISPERSIBLE	Methadone Hydrochloride	Methadone	LA
APAP/BUTALBITAL/CAFFEINE/CODEIN E	APAP/Butalbital/Caff/Codeine Phos	Codeine	SA

© 2023 Lyu X et al. *JAMA Network Open*.

APAP/CODEINE	Acetaminophen/Codeine Phosphate	Codeine	SA
ENDODAN	ASA/Oxycodone HCl/Oxycodone Terephthalate	Oxycodone SA	SA
OXYCODONE HCL-ACETAMINOPHEN	Acetaminophen/Oxycodone Hydrochlo	Oxycodone SA	SA
ORAMORPH SR	Morphine Sulfate	Morphine LA	LA
A.P.C. W/CODEINE	A.P.C. W/Codeine	Codeine	SA
APAP/PROPOXYPHENE	Acetaminophen/Propoxyphene Hydrochloride	Propoxyphene	SA
APAP/PROPOXYPHENE	Acetaminophen/Propoxyphene Napsylate	Propoxyphene	SA
APAP/PROPOXYPHENE NAPSYLATE	Acetaminophen/Propoxyphene Napsylate	Propoxyphene	SA
OXYCODONE HCL-OXYCODONE TEREPHTHALATE-ASPIRIN	ASA/Oxycodone HCl/Oxycodone Terephthalate	Oxycodone SA	SA
APAP/HYDROCODONE BITARTRATE	Acetaminophen/Hydrocodone Bitartrate	Hydrocodone	SA
APAP/PROPOXYPHENE HCL	Acetaminophen/Propoxyphene Hydrochloride	Propoxyphene	SA
OXYCODONE W/APAP	Acetaminophen/Oxycodone Hydrochloride	Oxycodone SA	SA
OXYCODONE W/ASPIRIN	ASA/Oxycodone HCl/Oxycodone Terephthalate	Oxycodone SA	SA
EMBEDA	Morphine Sulfate/Naltrexone Hydrochloride	Morphine LA	LA
OXYCODONE HCL	Oxycodone Hydrochloride	Oxycodone LA	LA
APAP/PROPOXYPHENE NAPSYLATE	Propoxyphene Napsylate	Propoxyphene	SA
OXYCODONE-APAP	Acetaminophen/Oxycodone Hydrochloride	Oxycodone SA	SA
OXYFAST	Oxycodone Hydrochloride	Oxycodone SA	SA
PANACET 5/500	Acetaminophen/Hydrocodone Bitartrate	Hydrocodone	SA
PANASAL 5/500	Aspirin/Hydrocodone Bitartrate	Hydrocodone	SA
PANLOR	Acetaminophen/Hydrocodone Bitartrate	Hydrocodone	SA
PERCODAN	ASA/Oxycodone HCl/Oxycodone Terephthalate	Oxycodone SA	SA
PERCODAN DEMI	ASA/Oxycodone HCl/Oxycodone Terephthalate	Oxycodone SA	SA
PERCODAN-DEMI	ASA/Oxycodone HCl/Oxycodone Terephthalate	Oxycodone SA	SA
PERCOLONE	Oxycodone Hydrochloride	Oxycodone SA	SA
PERLOXX	Acetaminophen/Oxycodone Hydrochloride	Oxycodone SA	SA
POLYGESIC	Acetaminophen/Hydrocodone Bitartrate	Hydrocodone	SA
PRIMALEV	Acetaminophen/Oxycodone Hydrochloride	Oxycodone SA	SA
PRIMLEV	Acetaminophen/Oxycodone Hydrochlo	Oxycodone SA	SA
PROCET	Acetaminophen/Hydrocodone Bitartrate	Hydrocodone	SA
PROPAIN HC	Acetaminophen/Hydrocodone Bitartrate	Hydrocodone	SA
RESCUDOSE	Morphine Sulfate	Morphine SA	SA
RMS	Morphine Sulfate	Morphine SA	SA

© 2023 Lyu X et al. *JAMA Network Open*.

RO-CODONE	Acetaminophen/Hydrocodone Bitartrate	Hydrocodone	SA
ROGESIC #3	Acetaminophen/Hydrocodone Bitartrate	Hydrocodone	SA
ROXANOL	Morphine Sulfate	Morphine SA	SA
ROXANOL UD	Morphine Sulfate	Morphine SA	SA
ROXANOL-T	Morphine Sulfate	Morphine SA	SA
ROXICODONE INTENSOL	Oxycodone Hydrochloride	Oxycodone SA	SA
ROXIPRIN	ASA/Oxycodone HCl/Oxycodone Terephthalate	Oxycodone SA	SA
SENEFEN III	Acetaminophen/Hydrocodone Bitartrate	Hydrocodone	SA
SK-OXYCODONE W/APAP	Acetaminophen/Oxycodone Hydrochloride	Oxycodone SA	SA
SK-OXYCODONE W/ASPIRIN	ASA/Oxycodone HCl/Oxycodone Terephthalate	Oxycodone SA	SA
STAGESIC-10	Acetaminophen/Hydrocodone Bitartrate	Hydrocodone	SA
EMBEDA	Morphine Sulfate/Naltrexon	Morphine LA	LA
CAPITAL W/CODEINE	Acetaminophen/Codeine Phosphate	Codeine	SA
T-GESIC	Acetaminophen/Hydrocodone Bitartrate	Hydrocodone	SA
T-GESIC FORTE	Acetaminophen/Hydrocodone Bitartrate	Hydrocodone	SA
UGESIC	Acetaminophen/Hydrocodone Bitartrate	Hydrocodone	SA
VANACET	Acetaminophen/Hydrocodone Bitartrate	Hydrocodone	SA
VAPOCET	Acetaminophen/Hydrocodone Bitartrate	Hydrocodone	SA
VENDONE	Acetaminophen/Hydrocodone Bitartrate	Hydrocodone	SA
VERDROCET	Acetaminophen/Hydrocodone Bitartrate	Hydrocodone	SA
XYLON 10	Hydrocodone Bitartrate/Ibu	Hydrocodone	SA
APAP/PROPROXYPHENE	Acetaminophen/Propoxyphene Hydrochloride	Propoxyphene	SA
APAP/PROPROXYPHENE NAPSYLATE	Acetaminophen/Propoxyphene Napsylate	Propoxyphene	SA
ASCRIPTIN W/CODEINE	Aspirin (Buffered)/Codeine Phosphate	Codeine	SA
ASPIRIN & CAFFEINE W/PROPOXYPHENE	Aspirin/Caffeine/Propoxyphene Hydrochloride	Propoxyphene	SA
ALLAY	Acetaminophen/Hydrocodone Bitartrate	Hydrocodone	SA
ASPIRIN W/CODEINE	Aspirin/Codeine Phosphate	Codeine	SA
ASPIRIN, CAFFEINE, AND DIHYDROCODEINE BITARTRATE	Aspirin/Caffeine/Dihydrocodeine Bitartrate	Dihydrocodein e	SA
ASPIRIN/BUTALBITAL/CAFFEINE/CODE INE	Aspirin/Butalbital/Caffeine/Codeine Phosphate	Codeine	SA
ASPIRIN/CAFFEINE/PROPOXYPHENE	Aspirin/Caffeine/Propoxyphene Hydrochloride	Propoxyphene	SA
ASPIRIN/CAFFEINE/PROPOXYPHENE HYDROCHLORIDE	Aspirin/Caffeine/Propoxyphene Hydrochloride	Propoxyphene	SA
ASPIRIN/CODEINE	Aspirin/Codeine Phosphate	Codeine	SA
B-A-C #3	Aspirin/Butalbital/Caffeine/Codeine Phosphate	Codeine	SA

© 2023 Lyu X et al. *JAMA Network Open*.

BALACET 325	Acetaminophen/Propoxyphene Napsylate	Propoxyphene	SA
BEXOPHENE	Aspirin/Caffeine/Propoxyphene Hydrochloride	Propoxyphene	SA
BUFFERIN W/CODEINE	Aspirin (Buffered)/Codeine Phosphate	Codeine	SA
BUT/APAP/CAFF/COD	APAP/Butalbital/Caff/Codeine Phos	Codeine	SA
BUTAL/APAP/CAFF/COD	APAP/Butalbital/Caff/Codeine Phos	Codeine	SA
BUTALBITAL COMPOUND W/CODEINE	Aspirin/Butalbital/Caffeine/Codeine Phosphate	Codeine	SA
BUTALBITAL COMPOUND/CODEINE	Aspirin/Butalbital/Caffeine/Codeine Phosphate	Codeine	SA
BUTALBITAL/APAP/CAFF/COD	APAP/Butalbital/Caff/Codeine Phos	Codeine	SA
BUTALBITAL/APAP/CAFFEINE/CODEIN E	APAP/Butalbital/Caff/Codeine Phos	Codeine	SA
BUTALBITAL/ASPIRIN/CAFFEINE W/CODEI	Aspirin/Butalbital/Caffeine/Codeine Phosphate	Codeine	SA
BUTALBITAL/ASPIRIN/CAFFEINE/CODE INE	Aspirin/Butalbital/Caffeine/Codeine Phosphate	Codeine	SA
ALOR 5/500	Aspirin/Hydrocodone Bitartrate	Hydrocodone	SA
AMACODONE	Acetaminophen/Hydrocodone Bitartrate	Hydrocodone	SA
BUTORPHANOL	Butorphanol Tartrate	Butorphanol	SA
COASTALDYNE	Acetaminophen/Codeine Phosphate	Codeine	SA
COCET	Acetaminophen/Codeine Phosphate	Codeine	SA
COCET PLUS	Acetaminophen/Codeine Phosphate	Codeine	SA
CODAPHEN	Acetaminophen/Codeine Phosphate	Codeine	SA
COTANAL-65	Propoxyphene Hydrochloride	Propoxyphene	SA
DARVOCET A500	Acetaminophen/Propoxyphene Napsylate	Propoxyphene	SA
DARVOCET-N 100	Acetaminophen/Propoxyphene Napsylate	Propoxyphene	SA
DARVOCET-N 50	Acetaminophen/Propoxyphene Napsylate	Propoxyphene	SA
DARVON	Propoxyphene Hydrochloride	Propoxyphene	SA
DARVON COMPOUND	Aspirin/Caffeine/Propoxyphene Hydrochloride	Propoxyphene	SA
DARVON COMPOUND 32	Aspirin/Caffeine/Propoxyphene Hydrochloride	Propoxyphene	SA
DARVON COMPOUND-65	Aspirin/Caffeine/Propoxyphene Hydrochloride	Propoxyphene	SA
DARVON-N	Propoxyphene Napsylate	Propoxyphene	SA
DHC PLUS	Acetaminophen/Caffeine/Dihydrocod eine Bitartrate	Dihydrocodein e	SA
DOLENE	Propoxyphene Hydrochloride	Propoxyphene	SA
DOLENE AP-65	Acetaminophen/Propoxyphene Hydrochloride	Propoxyphene	SA
DOLENE COMPOUND-65	Aspirin/Caffeine/Propoxyphene Hydrochloride	Propoxyphene	SA
DORAPHEN CO-65	Aspirin/Caffeine/Propoxyphene Hydrochloride	Propoxyphene	SA
DORAPHEN-65	Propoxyphene Hydrochloride	Propoxyphene	SA
DOXAPAP-N 100	Acetaminophen/Propoxyphene Napsylate	Propoxyphene	SA
DOXAPHENE COMPOUND	Aspirin/Caffeine/Propoxyphene Hydrochloride	Propoxyphene	SA
D-REX 65	Acetaminophen/Propoxyphene Hydrochloride	Propoxyphene	SA

© 2023 Lyu X et al. *JAMA Network Open*.

D-REX 65	Aspirin/Caffeine/Propoxyphene Hydrochloride	Propoxyphene	SA
D-REX 65	Propoxyphene Hydrochloride	Propoxyphene	SA
E-LOR	Acetaminophen/Propoxyphene Hydrochloride	Propoxyphene	SA
EMCODEINE	Aspirin/Codeine Phosphate	Codeine	SA
EMPIRIN W/CODEINE	Aspirin/Codeine Phosphate	Codeine	SA
EMPRACET	Acetaminophen/Codeine Phosphate	Codeine	SA
EZ III	Acetaminophen/Codeine Phosphate	Codeine	SA
FEBRIDYNE	Acetaminophen/Codeine Phosphate	Codeine	SA
FIORGEN W/CODEINE	Aspirin/Butalbital/Caffeine/Codeine Phosphate	Codeine	SA
FIORICET W/CODEINE	APAP/Butalbital/Caff/Codeine Phos	Codeine	SA
FIORTAL W/CODEINE	Aspirin/Butalbital/Caffeine/Codeine Phosphate	Codeine	SA
FUSEPAQ SYNAPRYN	Tramadol Hydrochloride	Tramadol	SA
G-3	APAP/Butabarbital Na/Codeine Phos	Codeine	SA
IDENAL	Aspirin/Butalbital/Caffeine/Codeine Phosphate	Codeine	SA
ISOBUTAL	Aspirin/Butalbital/Caffeine/Codeine Phosphate	Codeine	SA
ISOLLYL W/CODEINE	Aspirin/Butalbital/Caffeine/Codeine Phosphate	Codeine	SA
MARGESIC #3	Acetaminophen/Codeine Phosphate	Codeine	SA
MARGESIC 65	Propoxyphene Hydrochloride	Propoxyphene	SA
MARGESIC A-C	Aspirin/Caffeine/Propoxyphene Hydrochloride	Propoxyphene	SA
M-GESIC	Acetaminophen/Codeine Phosphate	Codeine	SA
MYGRANOL W/CODEINE	Aspirin/Butalbital/Caffeine/Codeine Phosphate	Codeine	SA
NALOXONE/PENTAZOCINE	Naloxone Hydrochloride/Pentazocine Hydrochloride	Pentazocine	SA
PANADOL W/CODEINE	Acetaminophen/Codeine Phosphate	Codeine	SA
PANCET	Acetaminophen/Propoxyphene Hydrochloride	Propoxyphene	SA
PANLOR DC	Acetaminophen/Caffeine/Dihydrocodeine Bitartrate	Dihydrocodeine	SA
PANLOR SS	Acetaminophen/Caffeine/Dihydrocodeine Bitartrate	Dihydrocodeine	SA
PANLOR-DC	Acetaminophen/Caffeine/Dihydrocodeine Bitartrate	Dihydrocodeine	SA
PANLOR-SS	Acetaminophen/Caffeine/Dihydrocodeine Bitartrate	Dihydrocodeine	SA
PC-CAP	Aspirin/Caffeine/Propoxyphene Hydrochloride	Propoxyphene	SA
PENTAZOCIN/NALOXONE	Naloxone Hydrochloride/Pentazocine Hydrochloride	Pentazocine	SA
PHENAPHEN W/CODEINE	Acetaminophen/Codeine Phosphate	Codeine	SA
PHENAPHEN-650 W/CODEINE	Acetaminophen/Codeine Phosphate	Codeine	SA
PHRENILIN W/CAFFEINE/CODEINE	APAP/Butalbital/Caff/Codeine Phos	Codeine	SA
PHRENILIN W/CODEINE	Acetaminophen/Butalbital/Codeine Phosphate	Codeine	SA
PHRENILIN WITH CAFFEINE AND CODEINE	APAP/Butalbital/Caff/Codeine Phos	Codeine	SA
PP-CAP	Propoxyphene Hydrochloride	Propoxyphene	SA
PRO POX 65	Propoxyphene Hydrochloride	Propoxyphene	SA

© 2023 Lyu X et al. *JAMA Network Open*.

PRO POX PLUS	Aspirin/Caffeine/Propoxyphene Hydrochloride	Propoxyphene	SA
PRO POX W/APAP	Acetaminophen/Propoxyphene Hydrochloride	Propoxyphene	SA
PRONAP-100	Acetaminophen/Propoxyphene Napsylate	Propoxyphene	SA
PROPACET 100	Acetaminophen/Propoxyphene Napsylate	Propoxyphene	SA
PROPOXACET	Acetaminophen/Propoxyphene Hydrochloride	Propoxyphene	SA
PROPOXACET-N	Acetaminophen/Propoxyphene Napsylate	Propoxyphene	SA
PROPOXACET-N 100	Acetaminophen/Propoxyphene Napsylate	Propoxyphene	SA
PROPOXY NAPS/APAP	Acetaminophen/Propoxyphene Napsylate	Propoxyphene	SA
PROPOXY NAPS/APAP-100	Acetaminophen/Propoxyphene Napsylate	Propoxyphene	SA
PROPOXY/APAP-65	Acetaminophen/Propoxyphene Hydrochloride	Propoxyphene	SA
PROPOXYCON	Propoxyphene Hydrochloride	Propoxyphene	SA
PROPOXYPHENE	Propoxyphene Hydrochloride	Propoxyphene	SA
PROPOXYPHENE COMPOUND	Aspirin/Caffeine/Propoxyphene Hydrochloride	Propoxyphene	SA
PROPOXYPHENE COMPOUND-65	Aspirin/Caffeine/Propoxyphene Hydrochloride	Propoxyphene	SA
PROPOXYPHENE HCL	Propoxyphene Hydrochloride	Propoxyphene	SA
PROPOXYPHENE HCL COMPOUND	Aspirin/Caffeine/Propoxyphene Hydrochloride	Propoxyphene	SA
PROPOXYPHENE HCL W/ACETAMINOPHEN	Acetaminophen/Propoxyphene Hydrochloride	Propoxyphene	SA
PROPOXYPHENE HYDROCHLORIDE	Propoxyphene Hydrochloride	Propoxyphene	SA
PROPOXYPHENE NAPSYLATE	Propoxyphene Napsylate	Propoxyphene	SA
PROPOXYPHENE NAPSYLATE & ACETAMINOPHEN	Acetaminophen/Propoxyphene Napsylate	Propoxyphene	SA
ANOLOR DH 5	Acetaminophen/Hydrocodone Bitartrate	Hydrocodone	SA
APAP W/HYDROCODONE	Acetaminophen/Hydrocodone Bitartrate	Hydrocodone	SA
PROPOXYPHENE NAPSYLATE AND ACETAMINOPHEN	Acetaminophen/Propoxyphene Napsylate	Propoxyphene	SA
PROPOXYPHENE NAPSYLATE W/APAP	Acetaminophen/Propoxyphene Napsylate	Propoxyphene	SA
HYDROGESIC	Acetaminophen/Hydrocodone Bitartrate	Hydrocodone	SA
PROPOXYPHENE NAPSYLATE/ACETAMINOPHEN	Acetaminophen/Propoxyphene Napsylate	Propoxyphene	SA
PROPOXYPHENE NAPSYLATE/APAP	Acetaminophen/Propoxyphene Napsylate	Propoxyphene	SA
PROPOXYPHENE W/ASPIRIN & CAFFEINE	Aspirin/Caffeine/Propoxyphene Hydrochloride	Propoxyphene	SA
PROPOXYPHENE-N W/ APAP	Acetaminophen/Propoxyphene Napsylate	Propoxyphene	SA
PROVAL	Acetaminophen/Codeine Phosphate	Codeine	SA
PYREGESIC-C	Acetaminophen/Codeine Phosphate	Codeine	SA
REGAL-DC	Dihydrocodeine/Apap/Caffeine	Dihydrocodein e	SA
SK-65	Propoxyphene Hydrochloride	Propoxyphene	SA

© 2023 Lyu X et al. *JAMA Network Open*.

SK-65 APAP	Acetaminophen/Propoxyphene Hydrochloride	Propoxyphene	SA
SK-65 COMPOUND	Aspirin/Caffeine/Propoxyphene Hydrochloride	Propoxyphene	SA
SK-APAP W/CODEINE	Acetaminophen/Codeine Phosphate	Codeine	SA
APAP W/OXYCODONE	Acetaminophen/Oxycodone Hydrochloride	Oxycodone SA	SA
SOMA COMPOUND W/CODEINE	Aspirin/Carisoprodol/Codeine Phosphate	Codeine	SA
STADOL NS	Butorphanol Tartrate	Butorphanol	SA
TALACEN	Acetaminophen/Pentazocine Hydrochloride	Pentazocine	SA
TALWIN COMPOUND	Aspirin/Pentazocine Hydrochloride	Pentazocine	SA
TALWIN NX	Naloxone Hydrochloride/Pentazocine Hydrochloride	Pentazocine	SA
TRAMADOL	Tramadol Hydrochloride	Tramadol	SA
HYDROMORPHONE HCL	Hydromorphone Hydrochloride	Hydromorphone	LA
HYDROMORPHONE HYDROCHLORIDE	Hydromorphone Hydrochloride	Hydromorphone	LA
HYSINGLA ER	Hydrocodone Bitartrate	Hydrocodone	LA
LEVO-DROMORAN	Levorphanol Tartrate	Levorphanol	LA
METHADONE	Methadone Hydrochloride	Methadone	LA
METHADONE DISP	Methadone Hydrochloride	Methadone	LA
APAP/HYDROCODONE	Acetaminophen/Hydrocodone Bitartrate	Hydrocodone	SA
ETH-OXYDOSE	Oxycodone Hydrochloride	Oxycodone SA	SA
METHADOSE DISP	Methadone Hydrochloride	Methadone	LA
TRAMADOL HCL/ACETAMINOPHEN	Acetaminophen/Tramadol Hydrochlor	Tramadol	SA
TRAMADOL HCL-ACETAMINOPHEN	Acetaminophen/Tramadol Hydrochloride	Tramadol	SA
APAP/OXYCODONE	Acetaminophen/Oxycodone Hydrochloride	Oxycodone SA	SA
ASPIRIN W/OXYCODONE	ASA/Oxycodone HCl/Oxycodone Terephthalate	Oxycodone SA	SA
ASPIRIN/OXYCODONE	ASA/Oxycodone HCl/Oxycodone Terephthalate	Oxycodone SA	SA
ASTRAMORPH	Morphine Sulfate	Morphine SA	SA
ASTRAMORPH/PF	Morphine Sulfate	Morphine SA	SA
AZDONE	Aspirin/Hydrocodone Bitartrate	Hydrocodone	SA
B & O SUPPRETTES 15A	Belladonna Alkaloids/Opium Alkaloids	Opium	SA
B & O SUPPRETTES 16A	Belladonna Alkaloids/Opium Alkaloids	Opium	SA
MORPHINE SULFATE CONTROLLED RELEASE	Morphine Sulfate	Morphine LA	LA
MORPHINE SULFATE CR	Morphine Sulfate	Morphine LA	LA
MORPHINE SULFATE ER	Morphine Sulfate	Morphine LA	LA
BANCAP H.C.	Acetaminophen/Hydrocodone Bitartrate	Hydrocodone	SA
DILAUDID-HP	Hydromorphone Hydrochloride	Hydromorphone	SA
A.B.C. W/CODEINE	Aspirin/Butalbital/Caffeine/Codeine Phosphate	Codeine	SA
TREZIX	Acetaminophen/Caffeine/Dih	Dihydrocodeine	SA

© 2023 Lyu X et al. *JAMA Network Open*.

TY-CAPS W/CODEINE	Acetaminophen/Codeine Phosphate	Codeine	SA
TYLAPRIN W/CODEINE	Acetaminophen/Codeine Phosphate	Codeine	SA
TYLENOL W/CODEINE	Acetaminophen/Codeine Phosphate	Codeine	SA
TYLENOL W/CODEINE #1	Acetaminophen/Codeine Phosphate	Codeine	SA
TYLENOL W/CODEINE #2	Acetaminophen/Codeine Phosphate	Codeine	SA
TYLENOL W/CODEINE #3	Acetaminophen/Codeine Phosphate	Codeine	SA
TYLENOL W/CODEINE #4	Acetaminophen/Codeine Phosphate	Codeine	SA
TYLENOL W/CODEINE NO 4	Acetaminophen/Codeine Phosphate	Codeine	SA
TYLENOL WITH CODEINE	Acetaminophen/Codeine Phosphate	Codeine	SA
ULTRACET	Acetaminophen/Tramadol Hydrochloride	Tramadol	SA
NOVAPLUS FENTANYL TRANSDERMAL SYSTEM	Fentanyl	Fentanyl LA	LA
ORLAAM	Levomethadyl Acetate Hydrochloride	Levomethadyl	LA
OXYCODONE	Oxycodone Hydrochloride	Oxycodone LA	LA
OXYCODONE CR	Oxycodone Hydrochloride	Oxycodone LA	LA
A.S.A. & CODEINE COMPOUND	Aspirin/Codeine Phosphate	Codeine	SA
ACETACO	Acetaminophen/Codeine Phosphate	Codeine	SA
ACETAMIN W/ CODEINE	Acetaminophen/Codeine Phosphate	Codeine	SA
ACETAMIN W/CODEINE	Acetaminophen/Codeine Phosphate	Codeine	SA
ACETAMIN/BUTAL/CAFF/COD	APAP/Butalbital/Caff/Codeine Phos	Codeine	SA
ACETAMINOPHEN	Acetaminophen/Codeine Phosphate	Codeine	SA
VOPAC	Acetaminophen/Codeine Phosphate	Codeine	SA
WYGESIC	Acetaminophen/Propoxyphene Hydrochloride	Propoxyphene	SA
ACETAMINOPHEN AND CODEINE PHOSPHATE #3	Acetaminophen/Codeine Phosphate	Codeine	SA
BANCAP HC	Acetaminophen/Hydrocodone Bitartrate	Hydrocodone	SA
BELLADONNA/OPIUM	Belladonna Alkaloids/Opium Alkaloids	Opium	SA
CEFACLOR	Morphine Sulfate	Morphine SA	SA
CETA PLUS	Acetaminophen/Hydrocodone Bitartrate	Hydrocodone	SA
CODEINE	Codeine Sulfate	Codeine	SA
CODEINE PHOSPHATE	Codeine Phosphate	Codeine	SA
CODEINE PHOSPHATE	Codeine Sulfate	Codeine	SA
ZERLOR	Acetaminophen/Caffeine/Dihydrocodeine Bitartrate	Dihydrocodeine	SA
OXYCODONE HYDROCHLORIDE CR	Oxycodone Hydrochloride	Oxycodone LA	LA
OXYCONTIN CR	Oxycodone Hydrochloride	Oxycodone LA	LA
OXYCONTIN HYDROCHLORIDE ER	Oxycodone Hydrochloride	Oxycodone LA	LA
OXYMORPHONE HCL	Oxymorphone Hydrochloride	Oxymorphone LA	LA
PALLADONE	Hydromorphone Hydrochloride	Hydromorphone	LA
ROXANOL	Morphine Sulfate	Morphine LA	LA
TRAMADOL HCL	Tramadol Hydrochloride	Tramadol	LA
ULTRAM	Tramadol Hydrochloride	Tramadol	LA
XARTEMIS XR	Acetaminophen/Oxycodone Hydrochloride	Oxycodone LA	LA
XARTEMIS XR	Acetaminophen/Oxycodone Hydrochlo	Oxycodone LA	LA
ZOHYDRO ER	Hydrocodone Bitartrate	Hydrocodone	LA

© 2023 Lyu X et al. *JAMA Network Open*.

COMBUNOX	Ibuprofen/Oxycodone Hydrochloride	Oxycodone SA	SA
DAMASON-P	Aspirin/Hydrocodone Bitartrate	Hydrocodone	SA
DAZIDOX	Oxycodone Hydrochloride	Oxycodone SA	SA
DEMEROL	Meperidine Hydrochloride	Meperidine	SA
DEMEROL APAP	Acetaminophen/Meperidine Hydrochloride	Meperidine	SA
DEMEROL HYDROCHLORIDE	Meperidine Hydrochloride	Meperidine	SA
ACETAMINOPHEN W/PROPOXYPHENE	Acetaminophen/Propoxyphene Hydrochloride	Propoxyphene	SA
ACETAMINOPHEN W/PROPOXYPHENE HCL	Acetaminophen/Propoxyphene Hydrochloride	Propoxyphene	SA
DILAUDID-5	Hydromorphone Hydrochloride	Hydromorphone	SA
DOLACET	Acetaminophen/Hydrocodone Bitartrate	Hydrocodone	SA
DOLAGESIC	Acetaminophen/Hydrocodone Bitartrate	Hydrocodone	SA
DOLFEN	Acetaminophen/Hydrocodone Bitartrate	Hydrocodone	SA
DOLO-PAP	Acetaminophen/Hydrocodone Bitartrate	Hydrocodone	SA
DOLOREX FORTE	Acetaminophen/Hydrocodone Bitartrate	Hydrocodone	SA
DUOCET	Acetaminophen/Hydrocodone Bitartrate	Hydrocodone	SA
DURADYNE DHC	Acetaminophen/Hydrocodone Bitartrate	Hydrocodone	SA
ACETAMINOPHEN AND CODEINE PHOSPHATE #4	Acetaminophen/Codeine Phosphate	Codeine	SA
ENDOCODONE	Oxycodone Hydrochloride	Oxycodone SA	SA
FENTANYL ORALET	Fentanyl Citrate	Fentanyl SA	SA
FENTANYL TROCHE	Fentanyl Citrate	Fentanyl SA	SA
GESIC 5	Acetaminophen/Hydrocodone Bitartrate	Hydrocodone	SA
HY-5	Acetaminophen/Hydrocodone Bitartrate	Hydrocodone	SA
HYCOGESIC	Acetaminophen/Hydrocodone Bitartrate	Hydrocodone	SA
HYCOMED	Acetaminophen/Hydrocodone Bitartrate	Hydrocodone	SA
HYCO-PAP	Acetaminophen/Hydrocodone Bitartrate	Hydrocodone	SA
HYCOTAB	Acetaminophen/Hydrocodone Bitartrate	Hydrocodone	SA
HYDROCET	Acetaminophen/Hydrocodone Bitartrate	Hydrocodone	SA
HYDROCOD BIT & ACET	Acetaminophen/Hydrocodone Bitartrate	Hydrocodone	SA
A.B.C. COMPOUND W/CODEINE	Aspirin/Butalbital/Caffeine/Codeine Phosphate	Codeine	SA
ACETAMINOPHEN W/PROPOXYPHENE HCL	Acetaminophen/Propoxyphene Napsylate	Propoxyphene	SA
ACETAMINOPHEN WITH CODEINE	Acetaminophen/Codeine Phosphate	Codeine	SA
ACETAMINOPHEN W/ CODEINE	Acetaminophen/Codeine Phosphate	Codeine	SA
HYDROCODONE BITARTRATE-ACETAMINOPHEN	Acetaminophen/Hydrocodone Bitartr	Hydrocodone	SA

© 2023 Lyu X et al. *JAMA Network Open*.

ACETAMINOPHEN/COD #4	Acetaminophen/Codeine Phosphate	Codeine	SA
A.P.C. W/BUTALBITAL & CODEINE	Aspirin/Butalbital/Caffeine/Codeine Phosphate	Codeine	SA
HYDROCODONE BITARTRATE-IBUPROFEN	Hydrocodone Bitartrate/Ibu	Hydrocodone	SA
HYDROCODONE W/APAP	Acetaminophen/Hydrocodone Bitartrate	Hydrocodone	SA
HYDROMORPHONE	Hydromorphone Hydrochloride	Hydromorphone	SA
HYDROMORPHONE HCL	Hydromorphone Hydrochloride	Hydromorphone SA	SA
OXYMORPHONE HCL	Oxymorphone Hydrochloride	Oxymorphone SA	SA
VICOPROFEN	Hydrocodone Bitartrate/Ibuprofen	Hydrocodone SA	SA
VICODIN	Acetaminophen/Hydrocodone Bitartrate	Hydrocodone SA	SA
VICODIN ES	Acetaminophen/Hydrocodone Bitartrate	Hydrocodone SA	SA
VICODIN HP	Acetaminophen/Hydrocodone Bitartrate	Hydrocodone SA	SA
TRAMADOL HCL	Tramadol Hydrochloride	Tramadol SA	SA
HYDROCODONE BITARTRATE-IBUPROFEN	Hydrocodone Bitartrate/Ibuprofen	Hydrocodone SA	SA
HYDROCODONE BITARTRATE-ACETAMINOPHEN	Acetaminophen/Hydrocodone Bitartrate	Hydrocodone SA	SA
TRAMADOL HCL/ACETAMINOPHEN	Acetaminophen/Tramadol Hydrochloride	Tramadol SA	SA
HYDROMORPHONE HCL	Hydromorphone Hydrochloride	Hydromorphone LA	LA
BUTALBITAL-APAP-CAFFEINE-CODEINE	Apap/Butalbital/Caff/Codeine Phos	Codeine	SA
TRAMADOL HCL	Tramadol Hydrochloride	Tramadol LA	LA
EXALGO	Hydromorphone Hydrochloride	Hydromorphone LA	LA
ZOHYDRO ER	Hydrocodone Bitartrate	Hydrocodone LA	LA
ULTRACET	Acetaminophen/Tramadol Hydrochloride	Tramadol SA	SA
ULTRAM ER	Tramadol Hydrochloride	Tramadol LA	LA
ULTRAM	Tramadol Hydrochloride	Tramadol SA	SA
NUCYNTA	Tapentadol Hydrochloride	Tapentadol SA	SA
NUCYNTA ER	Tapentadol Hydrochloride	Tapentadol LA	LA
NORCO	Acetaminophen/Hydrocodone Bitartrate	Hydrocodone SA	SA
HYSINGLA ER	Hydrocodone Bitartrate	Hydrocodone LA	LA
DILAUDID	Hydromorphone Hydrochloride	Hydromorphone SA	SA
CONZIP	Tramadol Hydrochloride	Tramadol LA	LA
FIORICET WITH CODEINE	Apap/Butalbital/Caff/Codeine Phos	Codeine	SA
IBUDONE	Hydrocodone Bitartrate/Ibuprofen	Hydrocodone SA	SA
ACETAMINOPHEN-CAFFEINE-DIHYDROCODEINE	Acetaminophen/Caffeine/Dihydrocodeine Bitartrate	Dihydrocodeine	SA
XYLON 10	Hydrocodone Bitartrate/Ibuprofen	Hydrocodone SA	SA

© 2023 Lyu X et al. *JAMA Network Open*.

REPREXAIN	Hydrocodone Bitartrate/Ibuprofen	Hydrocodone SA	SA
LORTAB 10/325	Acetaminophen/Hydrocodone Bitartrate	Hydrocodone SA	SA
LORTAB 7.5/325	Acetaminophen/Hydrocodone Bitartrate	Hydrocodone SA	SA
OXAYDO	Oxycodone Hydrochloride	Oxycodone SA	SA
LORTAB 7.5/500	Acetaminophen/Hydrocodone Bitartrate	Hydrocodone SA	SA
ZAMICET	Acetaminophen/Hydrocodone Bitartrate	Hydrocodone SA	SA
LORTAB 10/500	Acetaminophen/Hydrocodone Bitartrate	Hydrocodone SA	SA
LORCET HD	Acetaminophen/Hydrocodone Bitartrate	Hydrocodone SA	SA
LORTAB 5/325	Acetaminophen/Hydrocodone Bitartrate	Hydrocodone SA	SA
LORCET PLUS	Acetaminophen/Hydrocodone Bitartrate	Hydrocodone SA	SA
RYBIX ODT	Tramadol Hydrochloride	Tramadol SA	SA
XODOL	Acetaminophen/Hydrocodone Bitartrate	Hydrocodone SA	SA
OXYCODONE HCL-ASPIRIN	Asa/Oxycodone Hcl/Oxycodone Terephthalate	Oxycodone SA	SA
XODOL 7.5/300	Acetaminophen/Hydrocodone Bitartrate	Hydrocodone SA	SA
XODOL 5/300	Acetaminophen/Hydrocodone Bitartrate	Hydrocodone SA	SA
HYDROCET	Acetaminophen/Hydrocodone Bitartrate	Hydrocodone SA	SA
LORTAB 5/500	Acetaminophen/Hydrocodone Bitartrate	Hydrocodone SA	SA
LORCET	Acetaminophen/Hydrocodone Bitartrate	Hydrocodone SA	SA
LORCET 10/650	Acetaminophen/Hydrocodone Bitartrate	Hydrocodone SA	SA
RYZOLT	Tramadol Hydrochloride	Tramadol LA	LA
BELBUCA	Buprenorphine	Buprenorphine	LA
XTAMPZA ER	Oxycodone	Oxycodone LA	LA
FENTANYL TRANSDERM	Fentanyl	Fentanyl LA	LA
BUTALBITAL/ASPIRIN	Aspirin/Butalbital/Caffeine/Codeine Phosphate	Codeine	SA
VERDROCET	Acetaminophen/Hydrocodone Bitartrate	Hydrocodone SA	SA
BUT/APAP/CAFF/COD	Apap/Butalbital/Caff/Codeine Phos	Codeine	SA
OXYCODONE HCL-ACETAMINOPHEN	Acetaminophen/oxycodone Hydrochloride	Oxycodone SA	SA
VICODIN	Acetaminophen/hydrocodone Bitartrate	Hydrocodone SA	SA
VICODIN ES	Acetaminophen/hydrocodone Bitartrate	Hydrocodone SA	SA
VICODIN HP	Acetaminophen/hydrocodone Bitartrate	Hydrocodone SA	SA
ACETAMINOPHEN-CODEINE PHOSPHATE	Acetaminophen/codeine Phosphate	Codeine	SA
HYDROCODONE BITARTRATE-ACETAMINOPHEN	Acetaminophen/hydrocodone Bitartrate	Hydrocodone SA	SA

© 2023 Lyu X et al. *JAMA Network Open*.

OXYCODONE HCL-IBUPROFEN	Ibuprofen/oxycodone Hydrochloride	Oxycodone SA	SA
TRAMADOL HCL/ACETAMINOPHEN	Acetaminophen/tramadol Hydrochloride	Tramadol SA	SA
BUTALBITAL/ASPIRIN/CAFFEINE/CODEINE	Aspirin/butalbital/caffeine/codeine Phosphate	Codeine	SA
PENTAZOCINE HCL-NALOXONE HCL	Naloxone Hydrochloride/pentazocine Hydrochloride	Pentazocine	SA
BUTALBITAL-APAP-CAFFEINE-CODEINE	Apap/butalbital/caff/codeine Phos	Codeine	SA
OXYCODONE HCL-ASPIRIN	Aspirin/oxycodone Hydrochloride	Oxycodone SA	SA
HYDROCODONE BITARTRATE-IBUPROFEN	Hydrocodone Bitartrate/ibuprofen	Hydrocodone SA	SA
XARTEMIS XR	Acetaminophen/oxycodone Hydrochloride	Oxycodone LA	LA
OXYCODONE HCL-ACETAMINO	Acetaminophen/Oxycodone Hydrochloride	Oxycodone SA	SA
SYNALGOS-DC	Aspirin/caffeine/dihydrocodeine Bitartrate	Dihydrocodeine	SA
TYLENOL WITH CODEINE NO. 3	Acetaminophen/codeine Phosphate	Codeine	SA
TYLENOL WITH CODEINE NO. 4	Acetaminophen/codeine Phosphate	Codeine	SA
ASCOMP W/CODEINE	Aspirin/butalbital/caffeine/codeine Phosphate	Codeine	SA
FIORICET WITH CODEINE	Apap/butalbital/caff/codeine Phos	Codeine	SA
NORCO	Acetaminophen/hydrocodone Bitartrate	Hydrocodone SA	SA
EMBEDA	Morphine Sulfate/naltrexone Hydrochloride	Morphine LA	LA
ENDOCET	Acetaminophen/oxycodone Hydrochloride	Oxycodone SA	SA
PERCOCET	Acetaminophen/oxycodone Hydrochloride	Oxycodone SA	SA
MORPHABOND ER	Morphine Sulfate	Morphine LA	LA
ZAMICET	Acetaminophen/hydrocodone Bitartrate	Hydrocodone SA	SA
PRIMLEV	Acetaminophen/oxycodone Hydrochloride	Oxycodone SA	SA
ACETAMINOPHEN-CAFFEINE-DIHYDROCODEINE	Acetaminophen/caffeine/dihydrocodeine Bitartrate	Dihydrocodeine	SA
ULTRACET	Acetaminophen/tramadol Hydrochloride	Tramadol SA	SA
IBUDONE	Hydrocodone Bitartrate/ibuprofen	Hydrocodone SA	SA
FIORINAL W/CODEINE	Aspirin/butalbital/caffeine/codeine Phosphate	Codeine	SA
REPREXAIN	Hydrocodone Bitartrate/ibuprofen	Hydrocodone SA	SA
TREZIX	Acetaminophen/caffeine/dihydrocodeine Bitartrate	Dihydrocodeine	SA
ARYMO ER	Morphine Sulfate	Morphine LA	LA
ROXICET	Acetaminophen/oxycodone Hydrochloride	Oxycodone SA	SA
LORTAB 5/325	Acetaminophen/hydrocodone Bitartrate	Hydrocodone SA	SA
LORTAB 7.5/325	Acetaminophen/hydrocodone Bitartrate	Hydrocodone SA	SA
ROXYBOND	Oxycodone Hydrochloride	Oxycodone SA	SA

© 2023 Lyu X et al. *JAMA Network Open*.

TRAMADOL HCL/ACETA	Acetaminophen/Tramadol Hydrochloride	Tramadol SA	SA
VICOPROFEN	Hydrocodone Bitartrate/ibuprofen	Hydrocodone SA	SA
LORTAB 10/325	Acetaminophen/hydrocodone Bitartrate	Hydrocodone SA	SA
ASPIRIN, CAFFEINE, AND DIHYDROCODEINE BITARTRATE	Aspirin/caffeine/dihydrocodeine Bitartrate	Dihydrocodein e	SA
XODOL 5/300	Acetaminophen/hydrocodone Bitartrate	Hydrocodone SA	SA
NALOCET	Acetaminophen/Oxycodone Hydrochloride	Oxycodone SA	SA
XODOL	Acetaminophen/hydrocodone Bitartrate	Hydrocodone SA	SA
XODOL 7.5/300	Acetaminophen/hydrocodone Bitartrate	Hydrocodone SA	SA
BUTALBITAL COMPOUND W/CODEINE	Aspirin/butalbital/caffeine/codeine Phosphate	Codeine	SA
TYLENOL W/CODEINE #3	Acetaminophen/codeine Phosphate	Codeine	SA
CAPITAL W/CODEINE	Acetaminophen/codeine Phosphate	Codeine	SA
ENDODAN	Aspirin/oxycodone Hydrochloride	Oxycodone SA	SA
BUT/APAP/CAFF/COD	Apap/butalbital/caff/codeine Phos	Codeine	SA

© 2023 Lyu X et al. *JAMA Network Open*.

eTable 2. Changes in slope (pre- vs. post-Guideline) by census region for each of the four primary outcomes

	Census Region			
Prescribing measures	**Northeast (N = 9)**	**Midwest (N = 12)**	**South (N = 17)**	**West (N = 13)**
Overall Dispensing Rate				
0.00 – 20.00	3 (33%)	4 (33%)	4 (24%)[1]	4 (31%)
20.01 – 30.00	2 (22%)	2 (17%)	4 (24%)	3 (23%)
30.01 – 40.00	2 (22%)	5 (42%)	5 (29%)	4 (31%)
>40.00	2 (22%)	1 (8%)	4 (24%)	2 (15%)
High-dosage (>90MME/Day) Dispensing Rate				
<0.00	0 (0%)	0 (0%)	2 (12%)[2]	1 (8%)[3]
0.00 – 3.00	2 (22%)[4]	6 (50%)	3 (18%)	2 (15%)
3.01 – 7.00	3 (33%)	3 (25%)	6 (35%)	6 (46%)
7.01 – 10.00	3 (33%)	3 (25%)	4 (24%)	3 (23%)
>10.00	1 (11%)	0 (0%)	2 (12%)	1 (8%)
Long-acting Dispensing Rate				
0.00 – 4.00	3 (33%)	5 (42%)	5 (29%)	2 (15%)
4.01 – 7.00	3 (33%)	5 (42%)	4 (24%)	7 (54%)
7.01 – 10.00	1 (11%)	2 (17%)	6 (35%)	4(31%)
>10.00	2 (22%)	0 (0%)	2 (12%)	0 (0%)
MME Dispensed per Capita				
0.0 – 0.2	1 (11%)	1 (8%)	4 (24%)[5]	2 (15%)
0.2 – 0.4	3 (33%)	6 (50%)	3 (18%)	4 (31%)
0.4 – 0.6	2 (22%)	3 (25%)	4 (24%)	4 (31%)
0.6+	3 (33%)	2 (17%)	6 (35%)	2 (15%)

Note: Categories are based on changes in the slope and correspond to dispensing rate before vs. after the release of the 2016 CDC Guideline.

[1] Three out of four states had statistically not significant changes.
[2] One out of two states had statistically not significant changes.
[3] One out of one state had statistically not significant changes.
[4] One out of two states had statistically not significant changes.
[5] One out of four states had statistically not significant changes.

© 2023 Lyu X et al. *JAMA Network Open*.

eFigure 1. Trends in the overall dispensing rate from 2012 through 2018, with linear regression lines showing the pre- and post-Guideline slopes superimposed

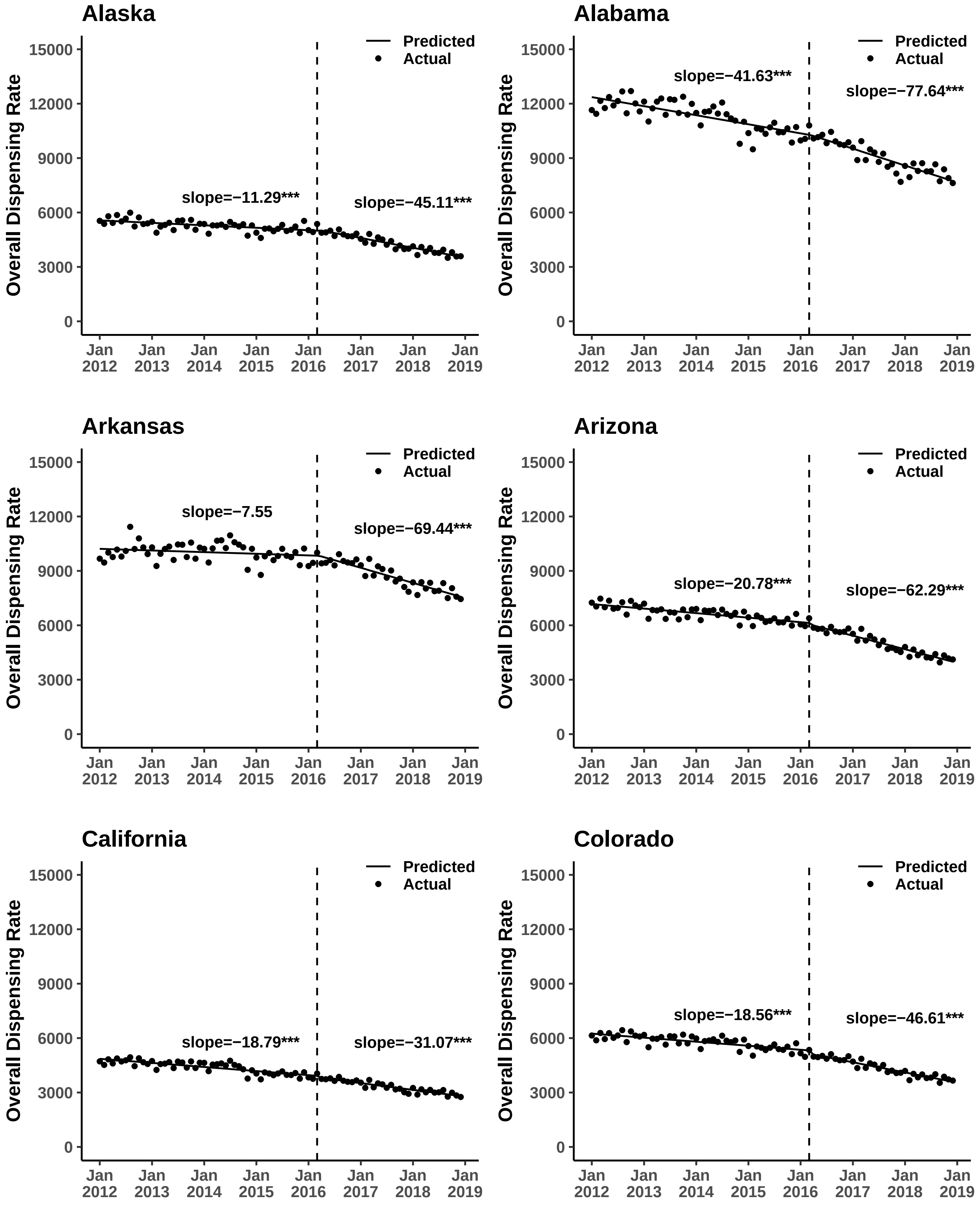

© 2023 Lyu X et al. *JAMA Network Open*.

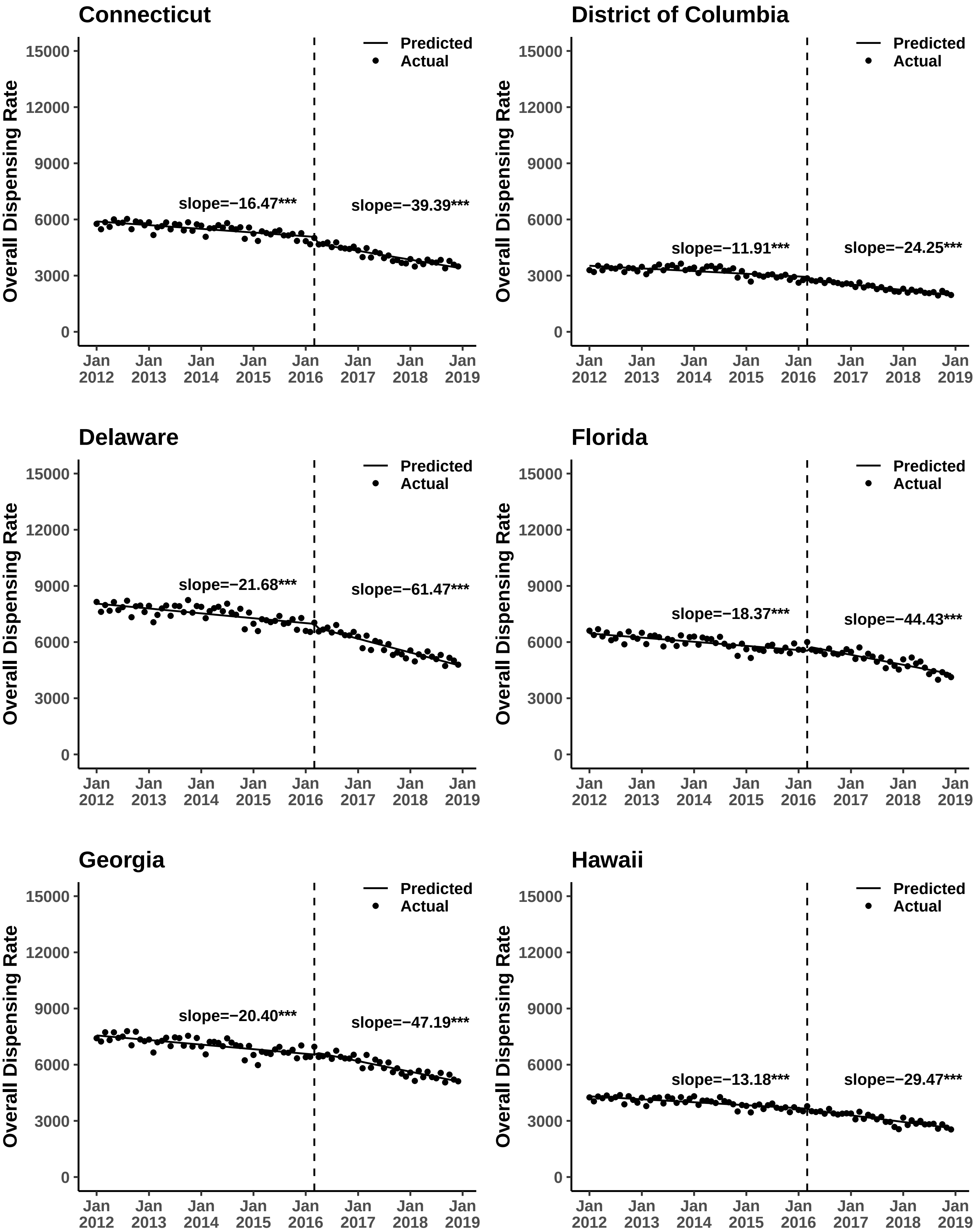

© 2023 Lyu X et al. *JAMA Network Open*.

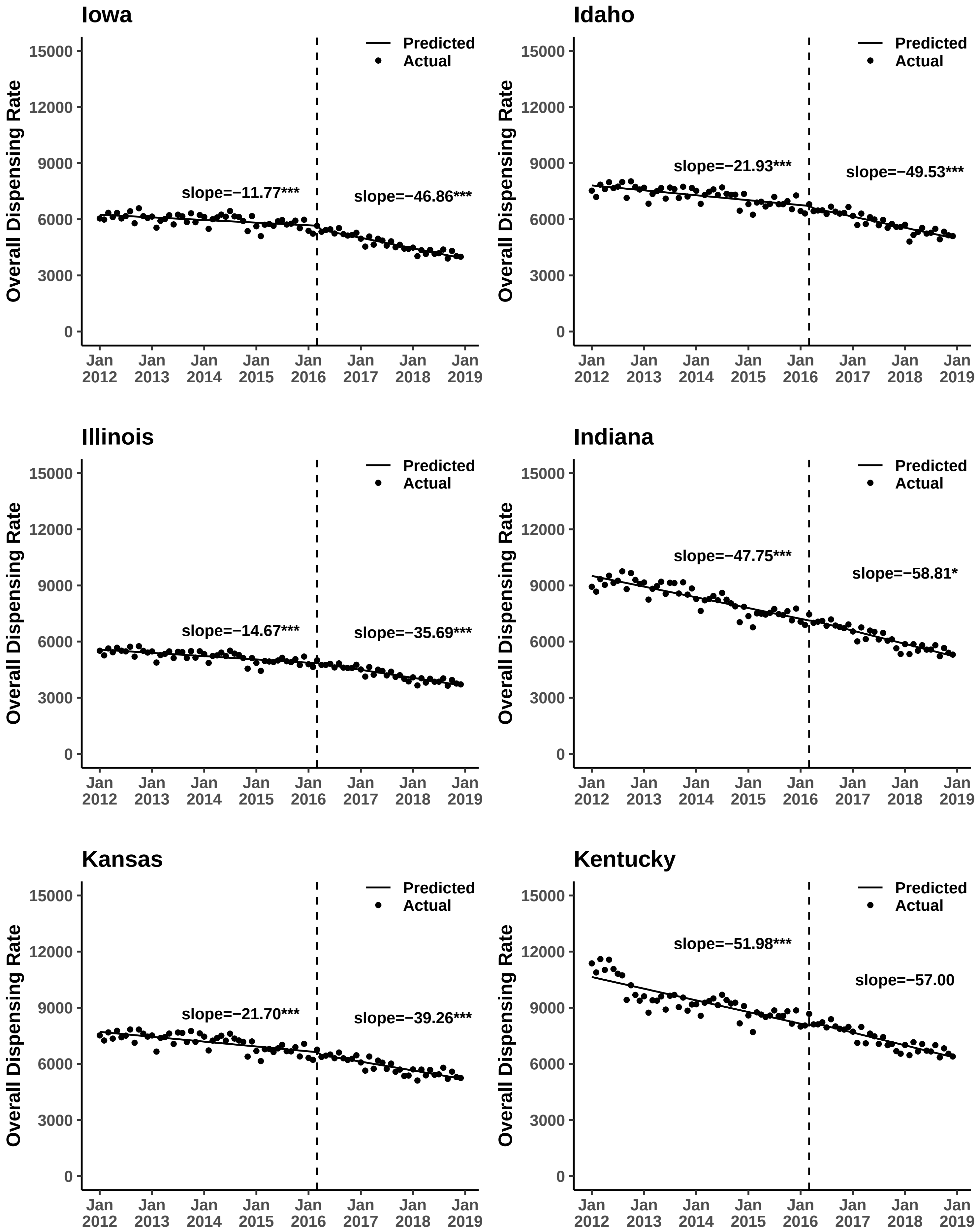

© 2023 Lyu X et al. *JAMA Network Open*.

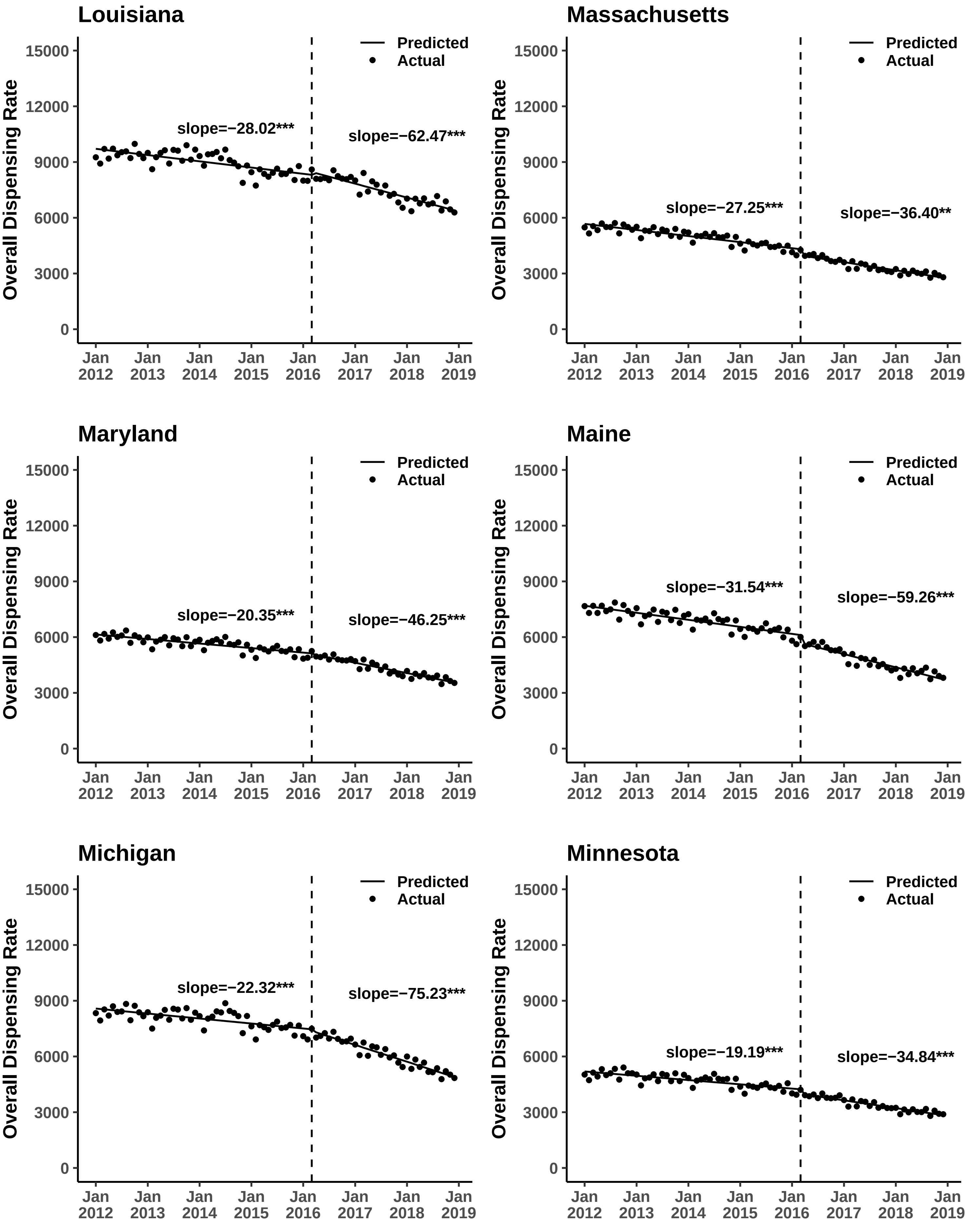

© 2023 Lyu X et al. *JAMA Network Open*.

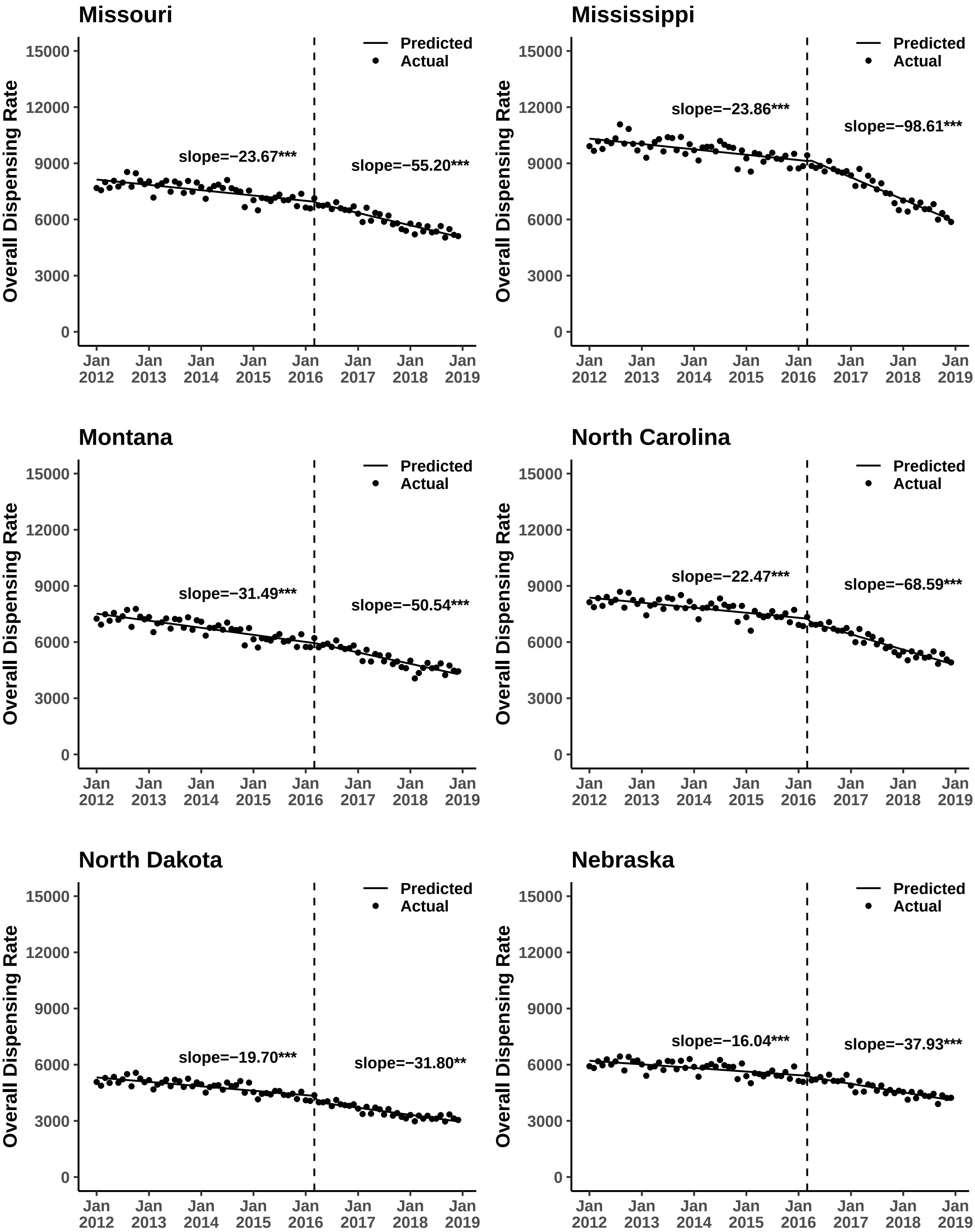

© 2023 Lyu X et al. *JAMA Network Open*.

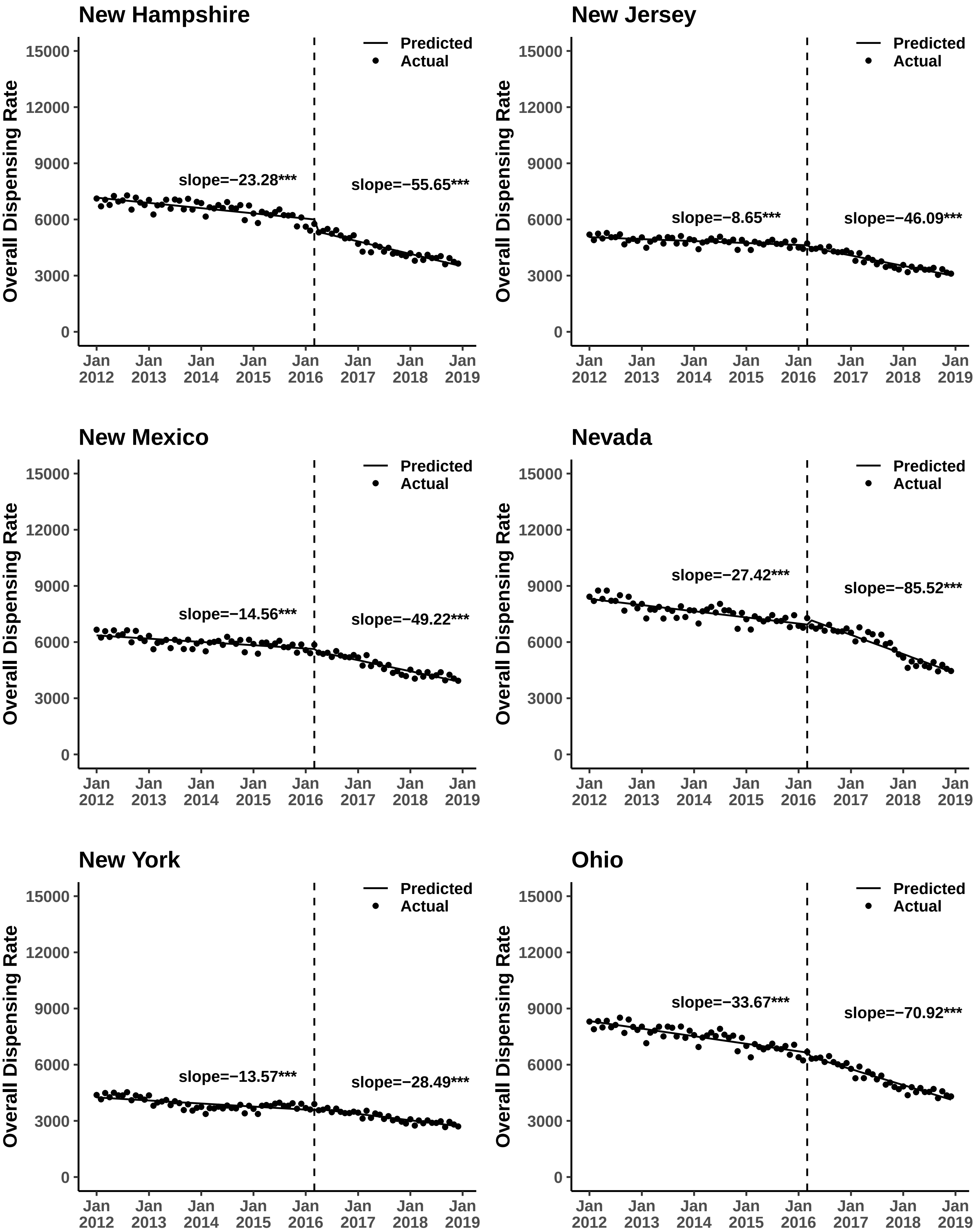

© 2023 Lyu X et al. *JAMA Network Open*.

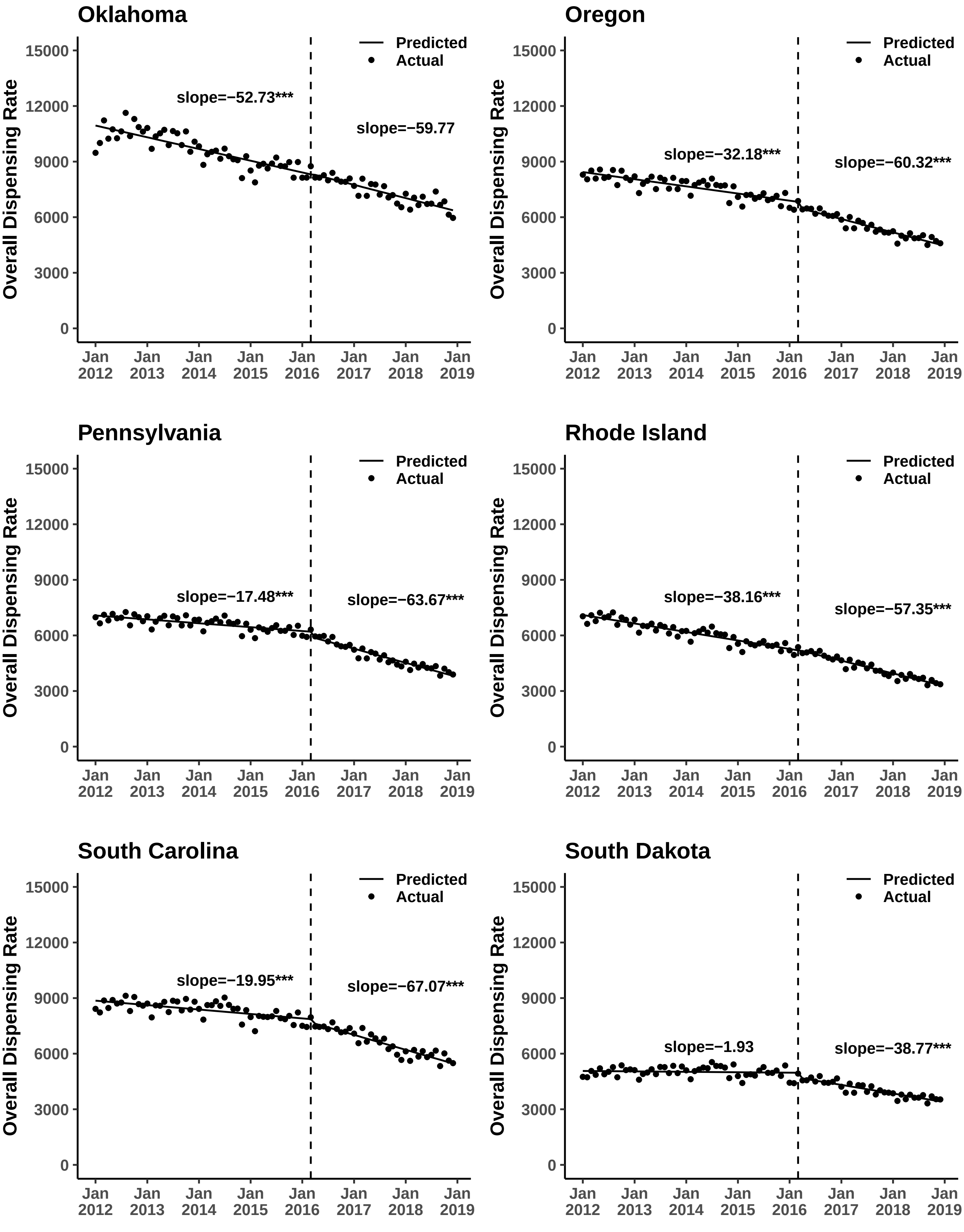

© 2023 Lyu X et al. *JAMA Network Open*.

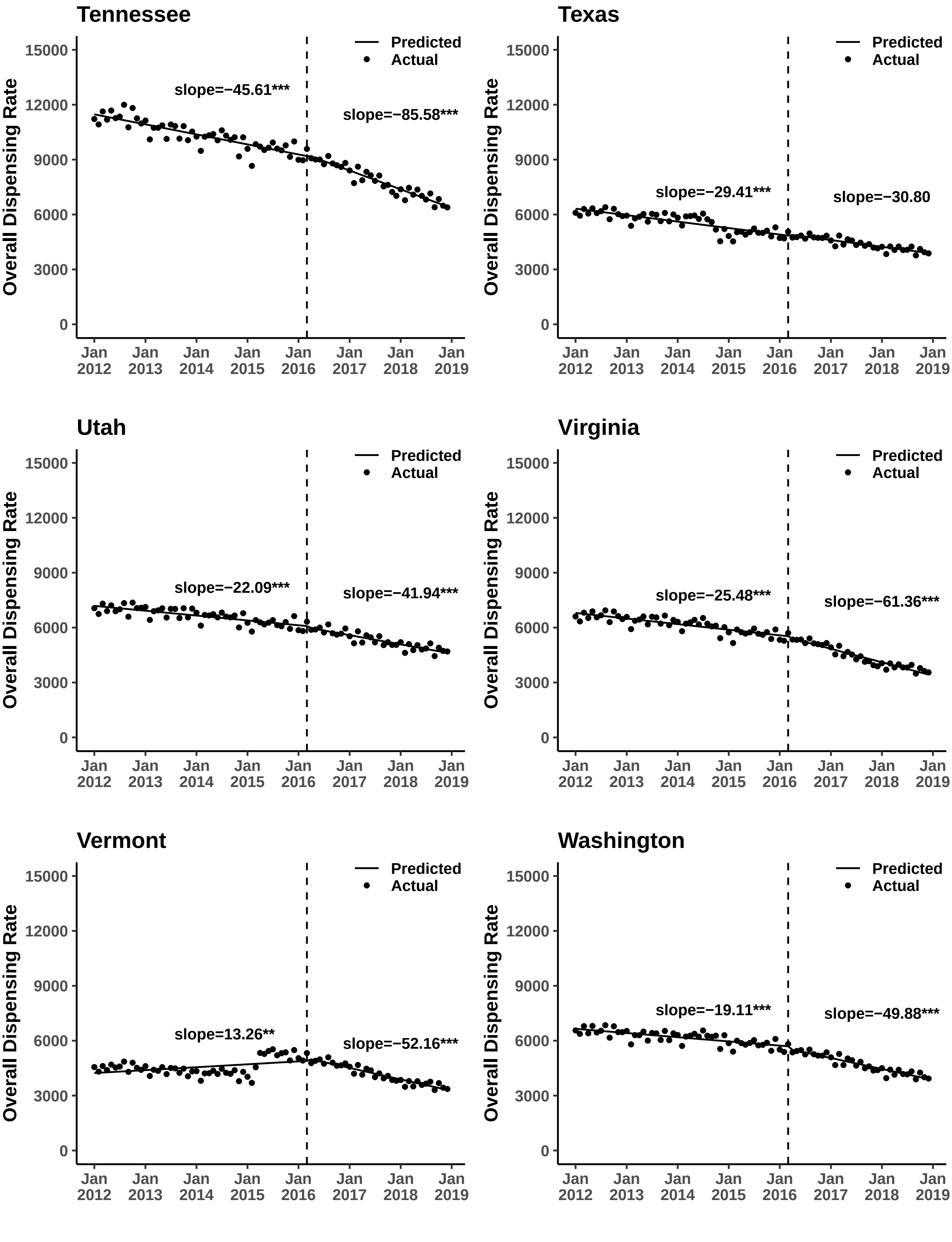

© 2023 Lyu X et al. *JAMA Network Open*.

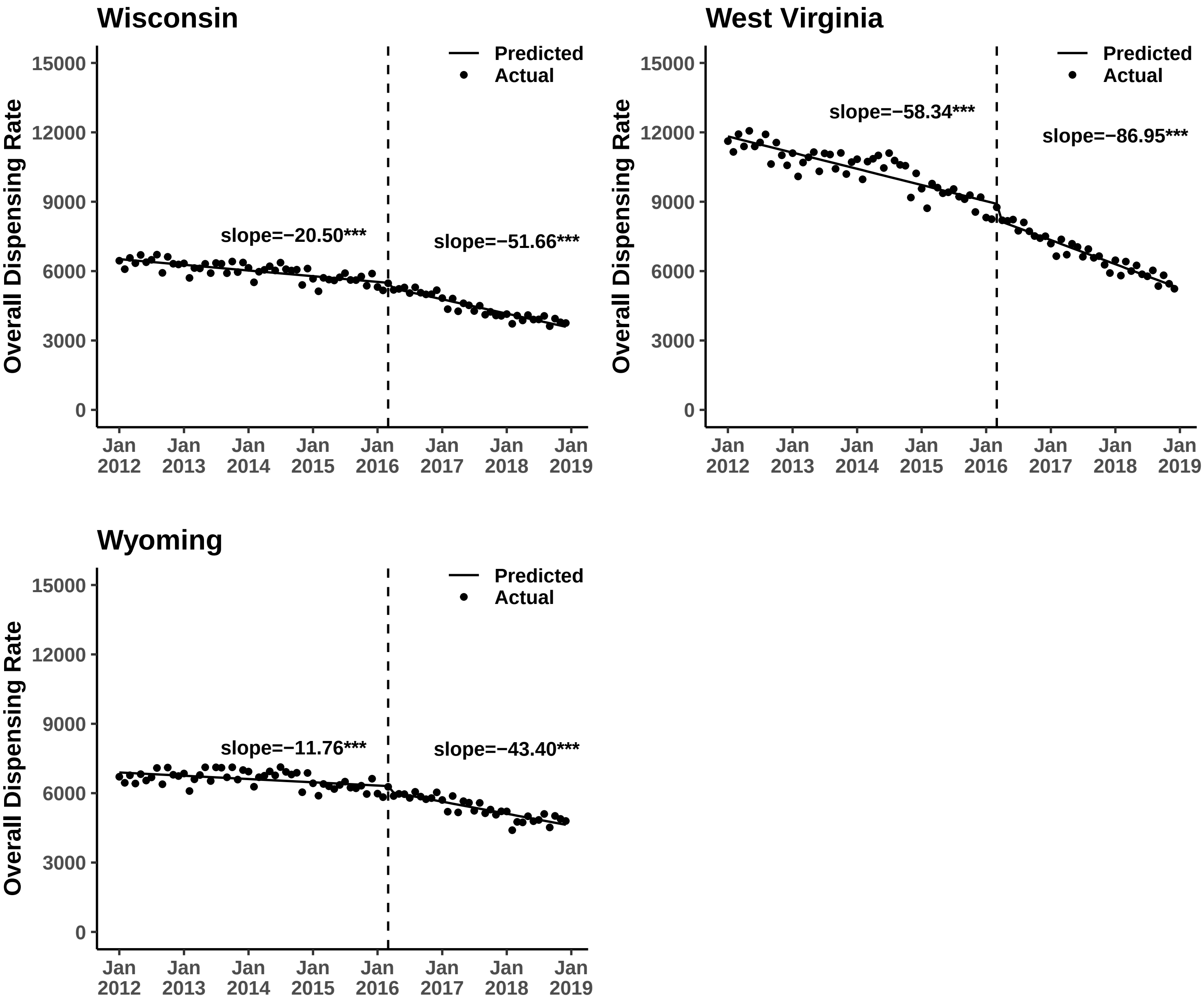

© 2023 Lyu X et al. *JAMA Network Open*.

eFigure 2. Trends in the long-acting dispensing rate from 2012 through 2018, with linear regression lines showing the pre- and post-Guideline slopes superimposed

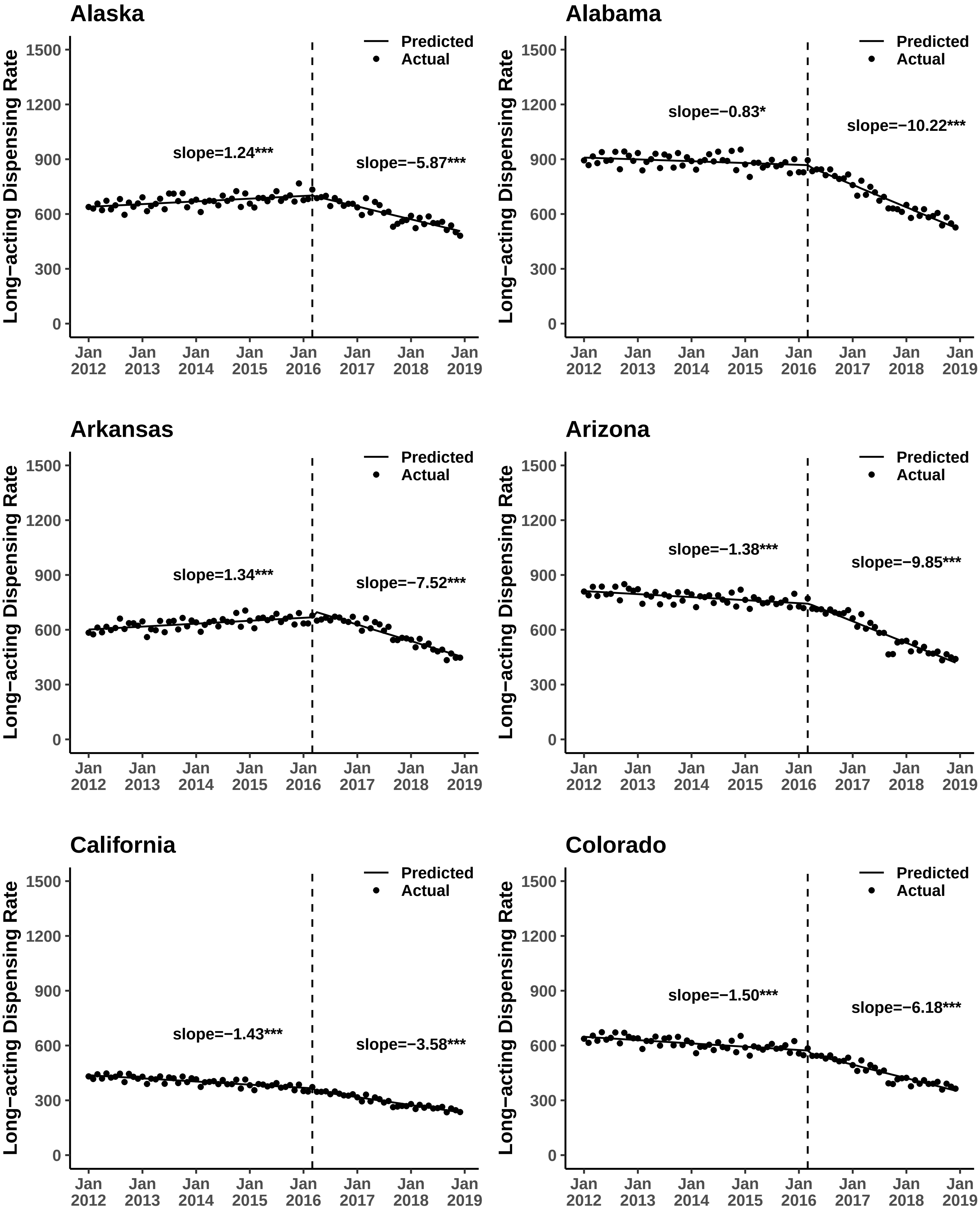

© 2023 Lyu X et al. *JAMA Network Open*.

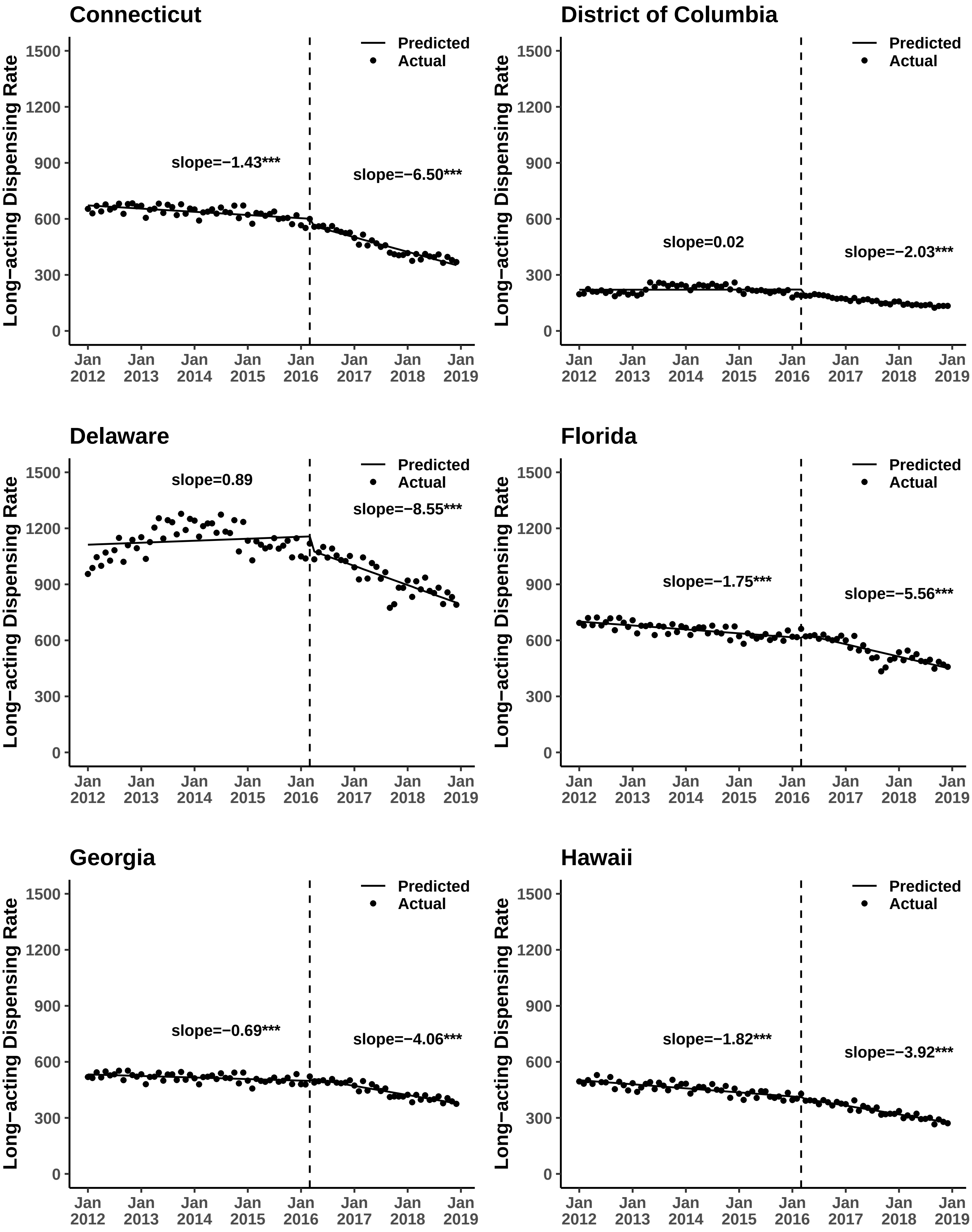

© 2023 Lyu X et al. *JAMA Network Open*.

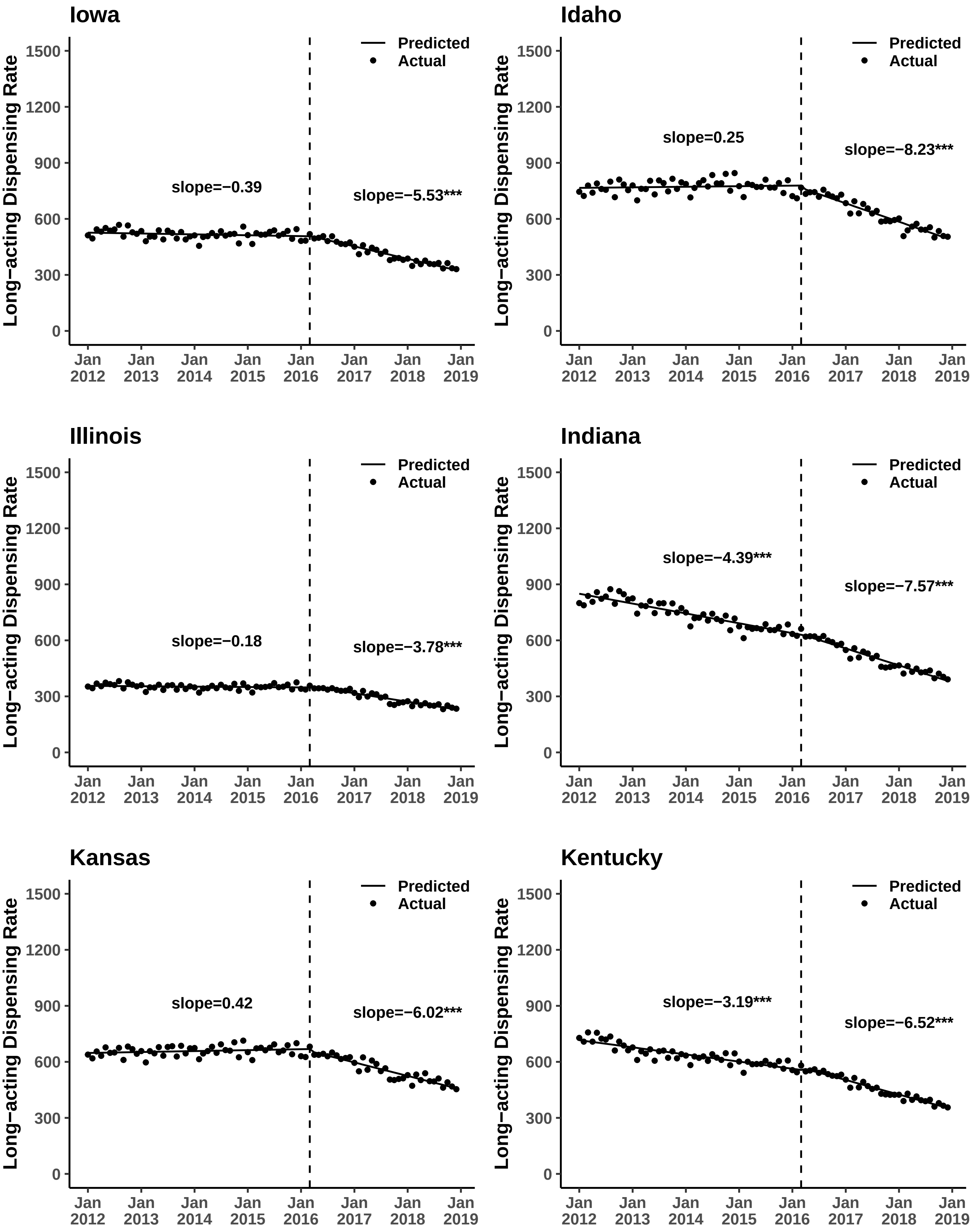

© 2023 Lyu X et al. *JAMA Network Open*.

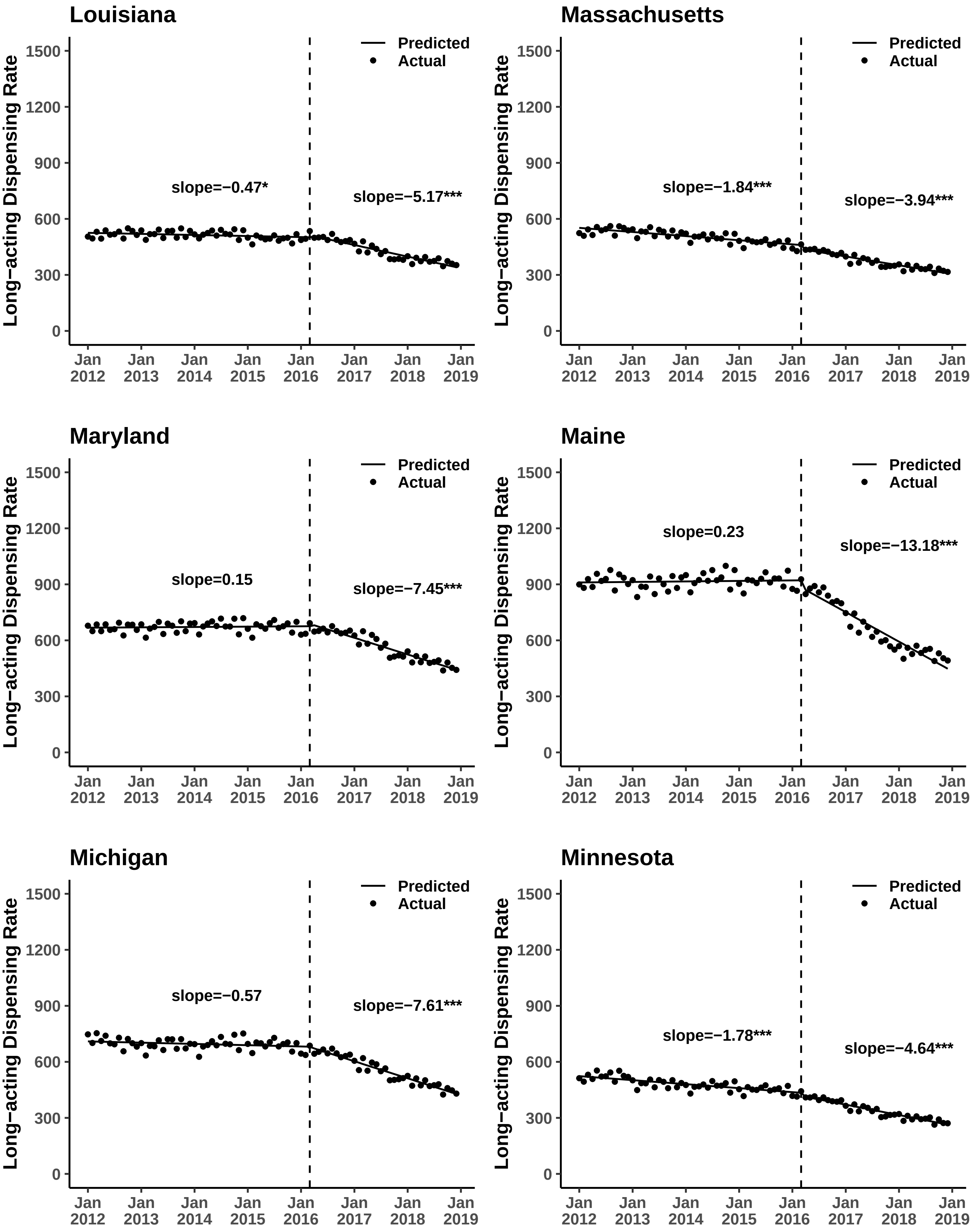

© 2023 Lyu X et al. *JAMA Network Open*.

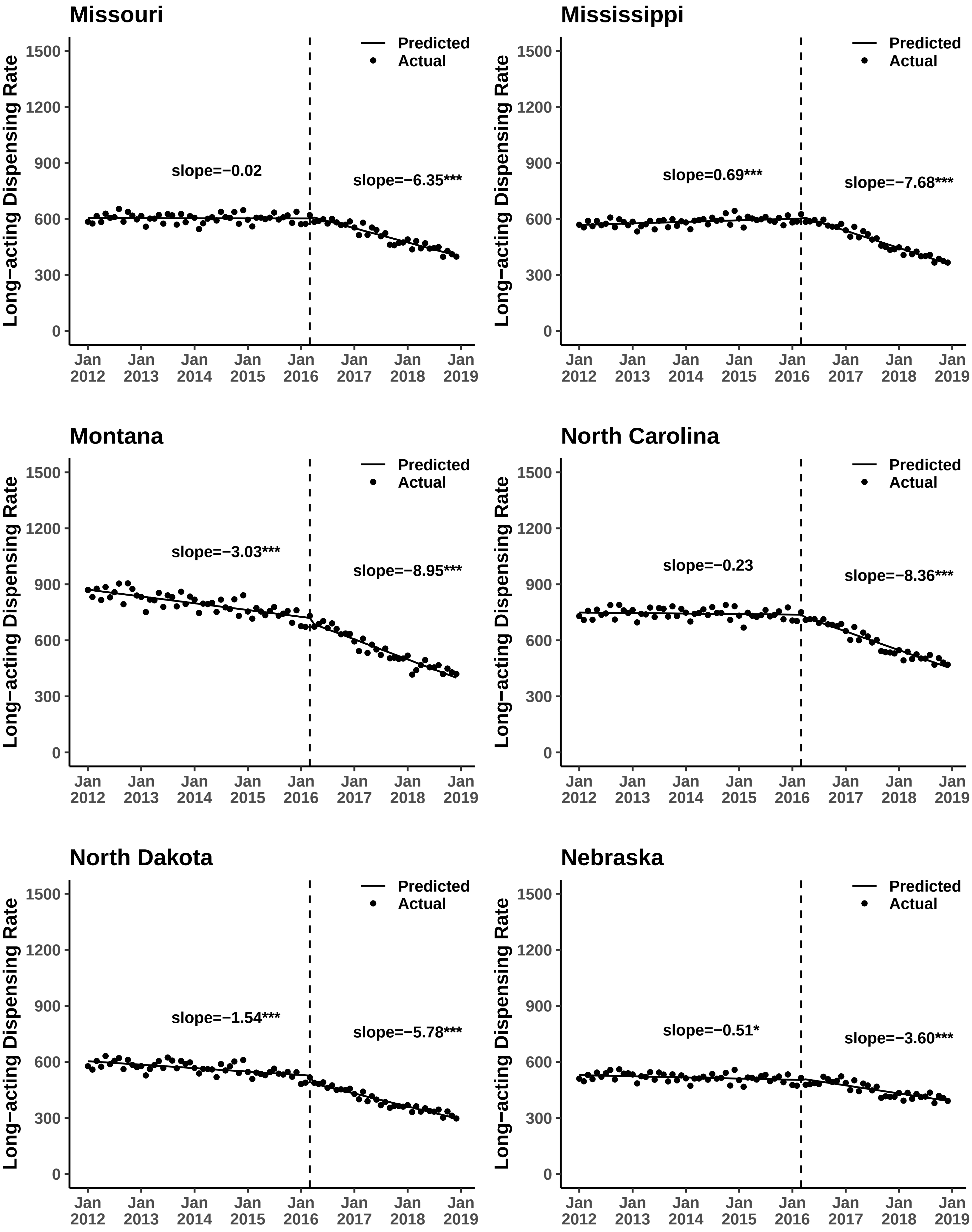

© 2023 Lyu X et al. *JAMA Network Open*.

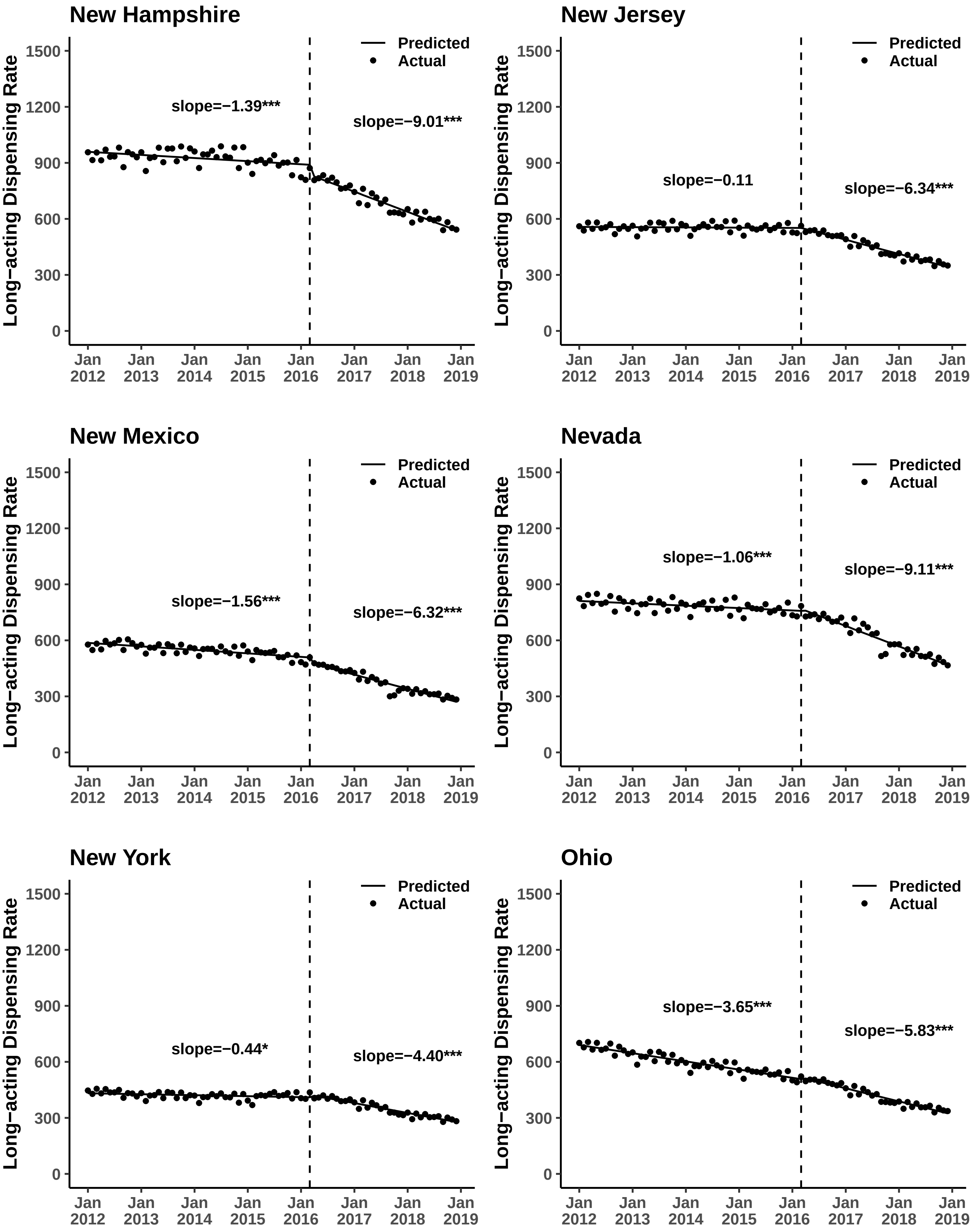

© 2023 Lyu X et al. *JAMA Network Open*.

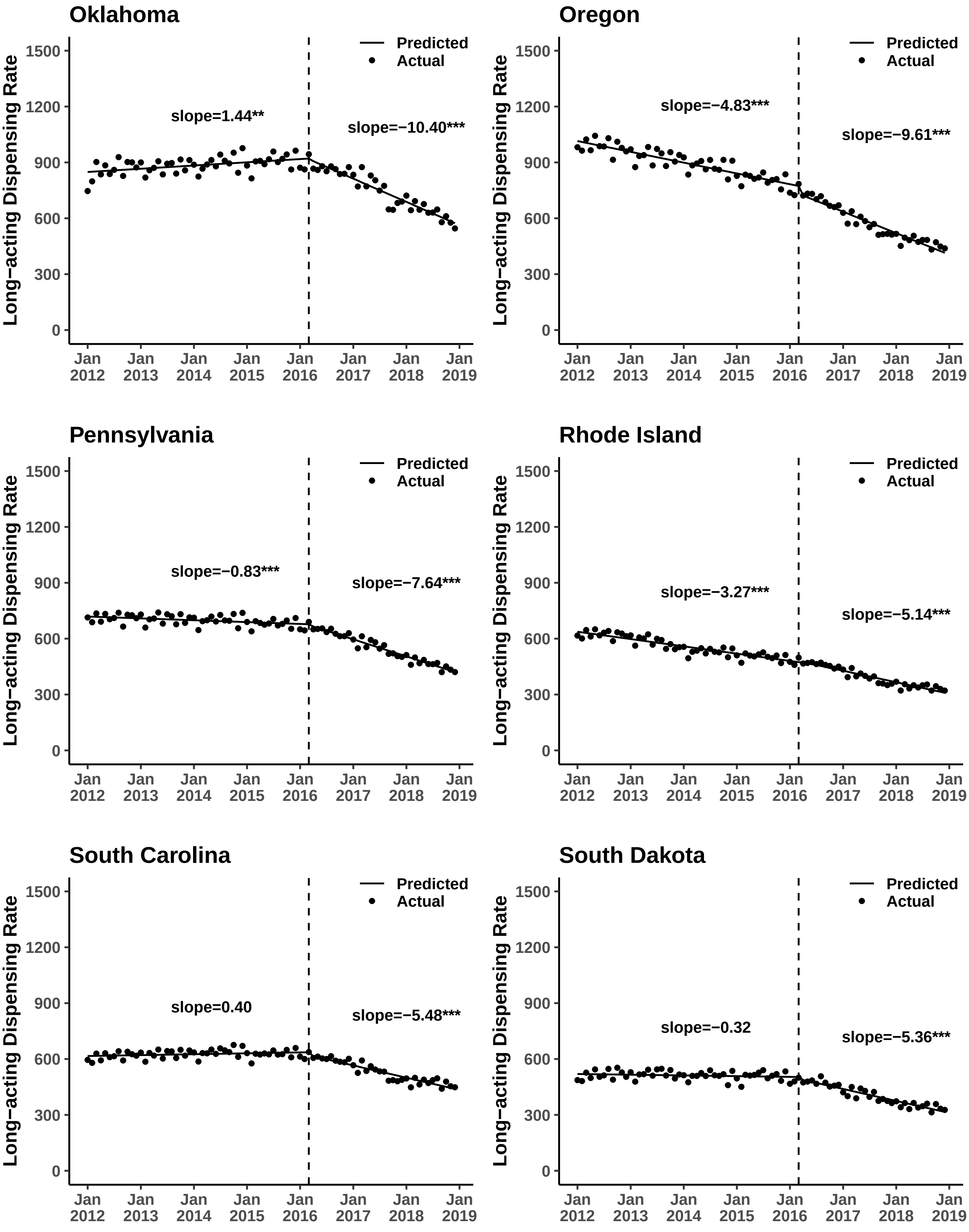

© 2023 Lyu X et al. *JAMA Network Open*.

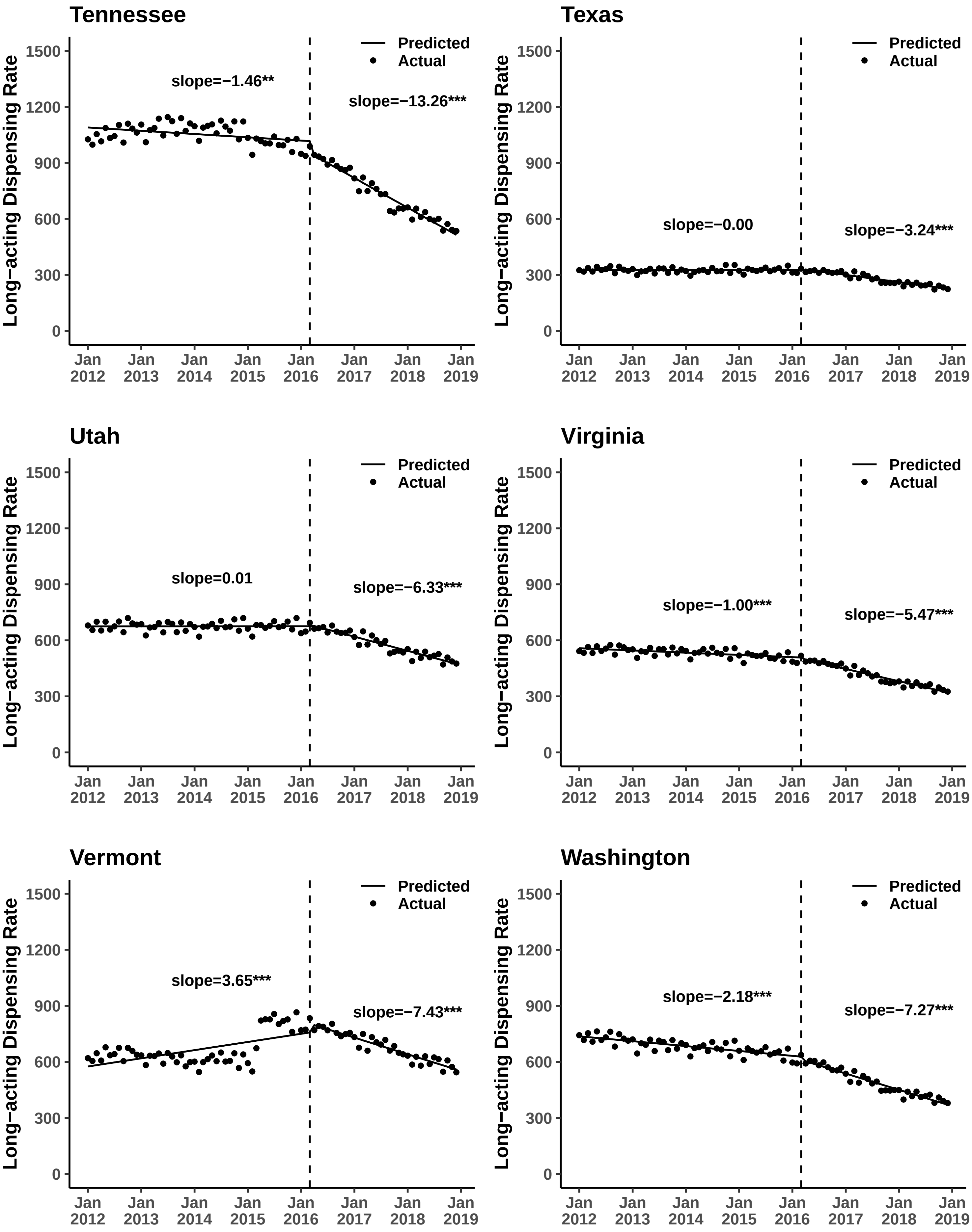

© 2023 Lyu X et al. *JAMA Network Open*.

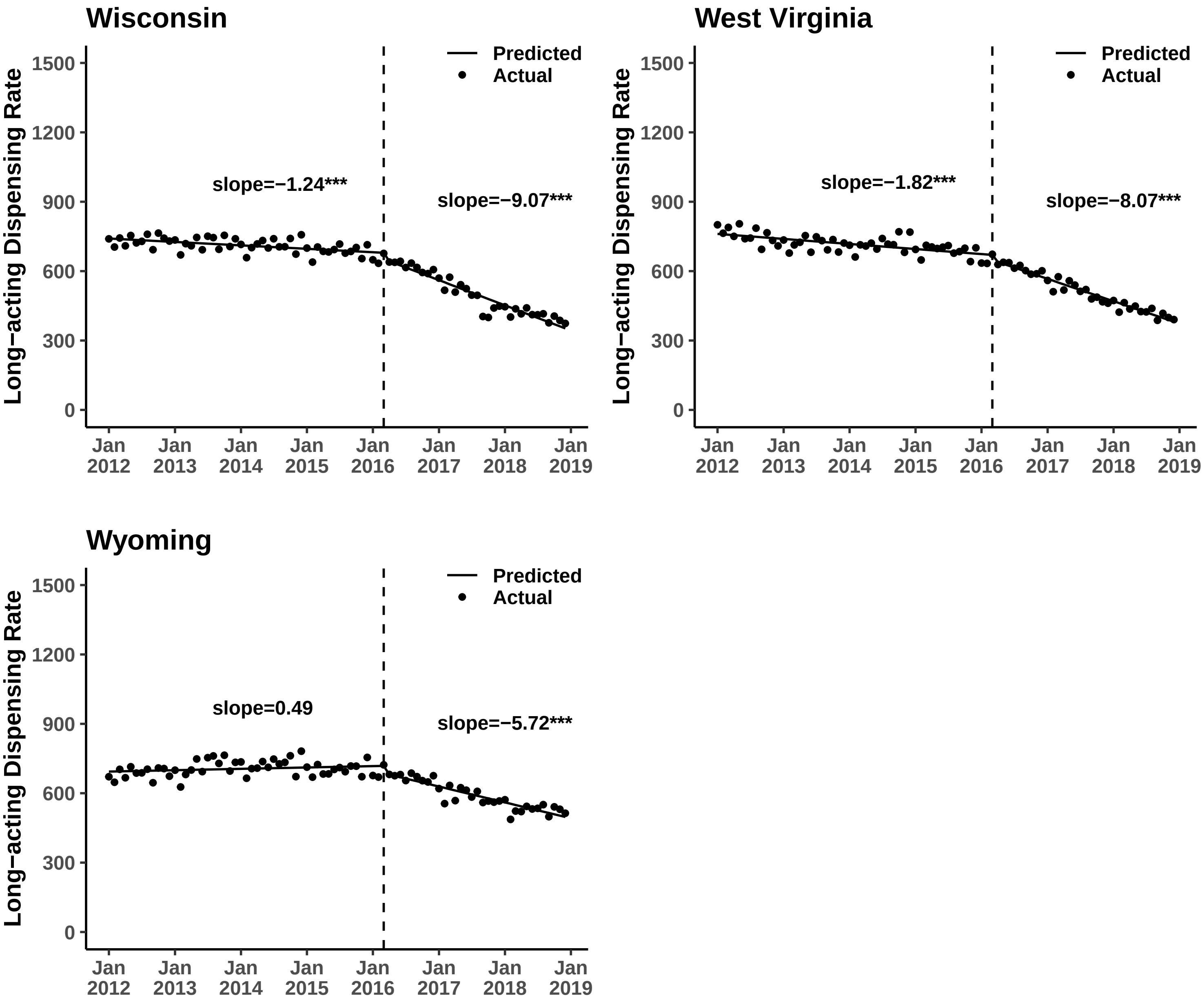

© 2023 Lyu X et al. *JAMA Network Open*.

eFigure 3. Trends in the high-dose (≥90 MME/Day) dispensing rate from 2012 through 2018, with linear regression lines showing the pre- and post-Guideline slopes superimposed

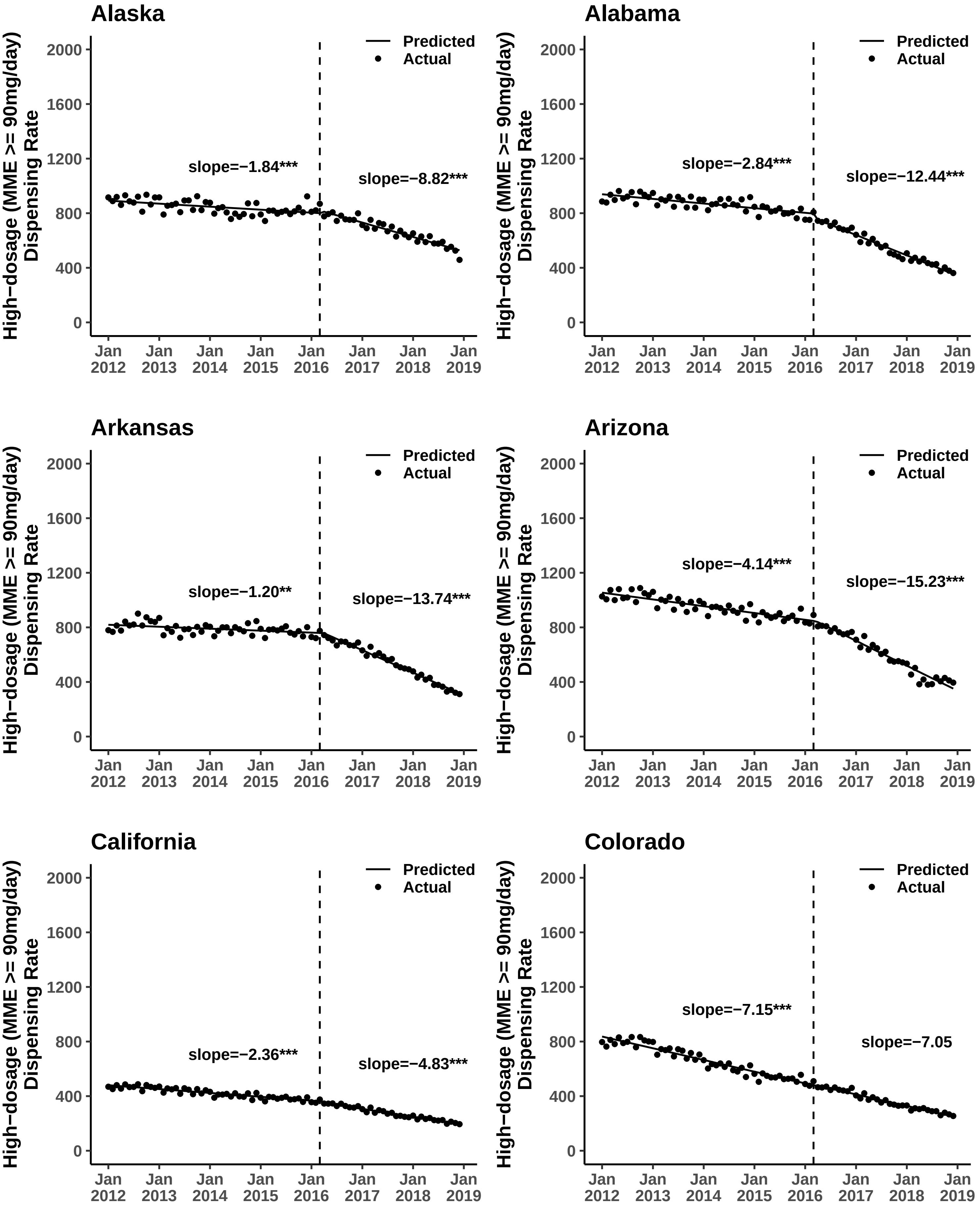

© 2023 Lyu X et al. *JAMA Network Open*.

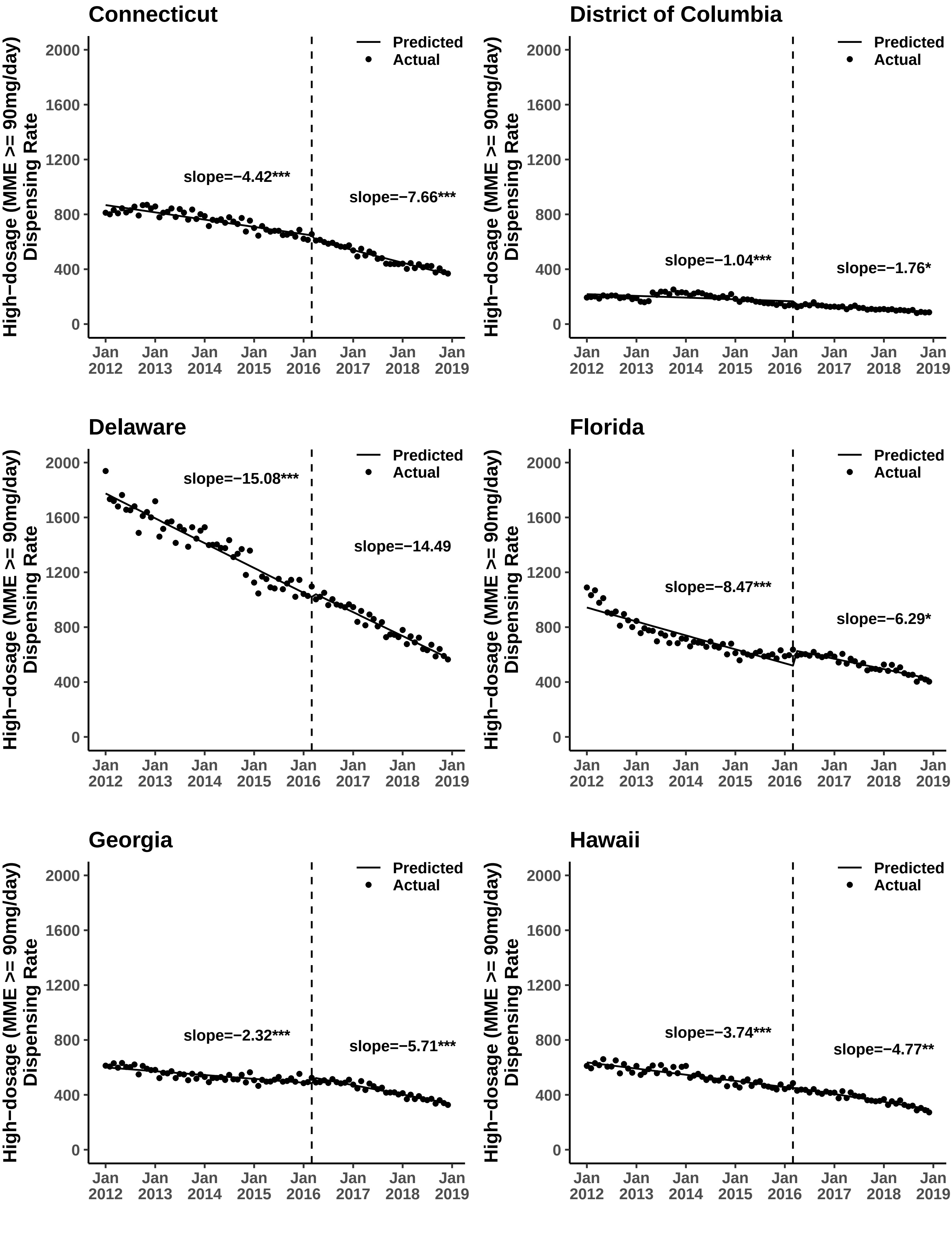

© 2023 Lyu X et al. *JAMA Network Open*.

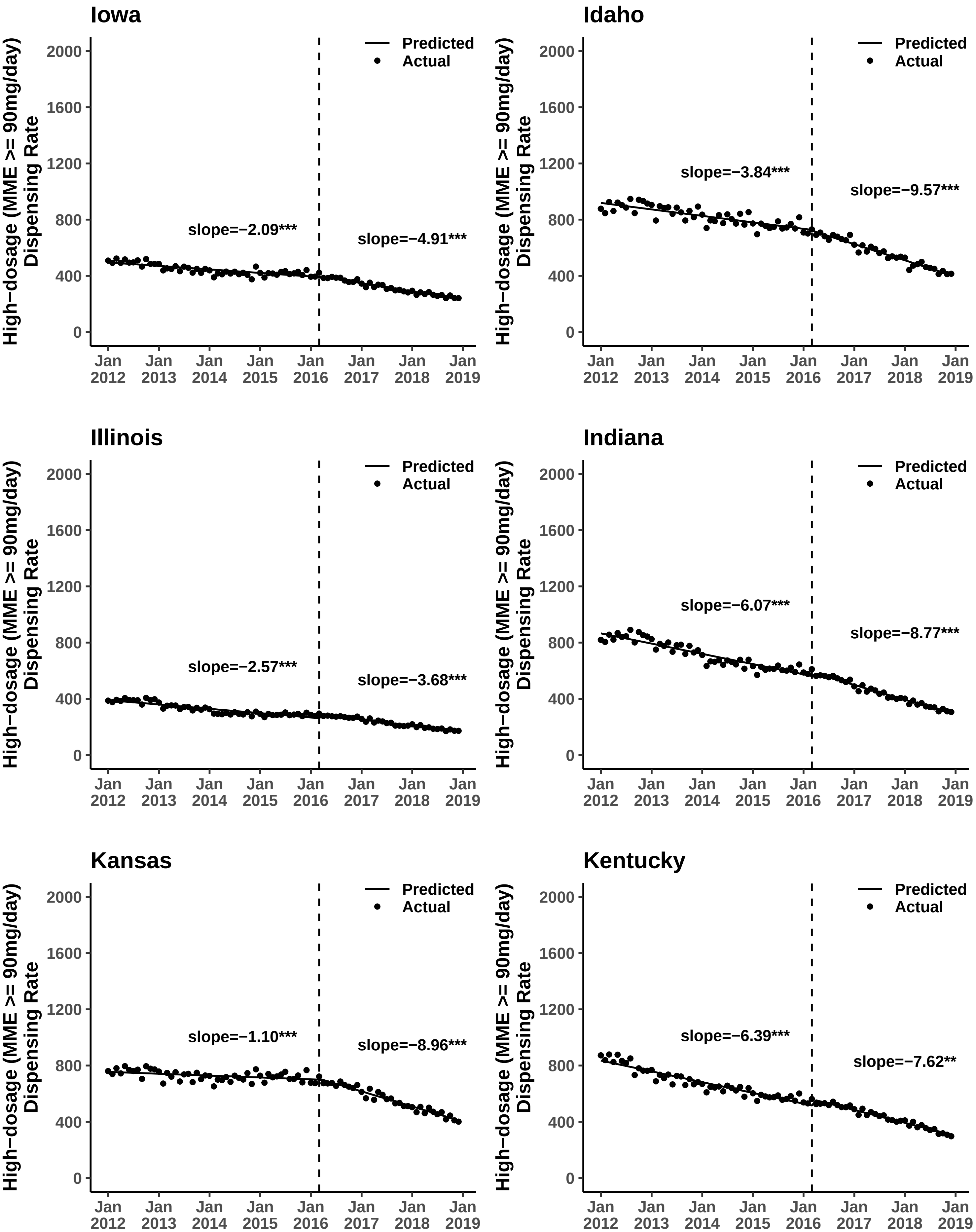

© 2023 Lyu X et al. *JAMA Network Open*.

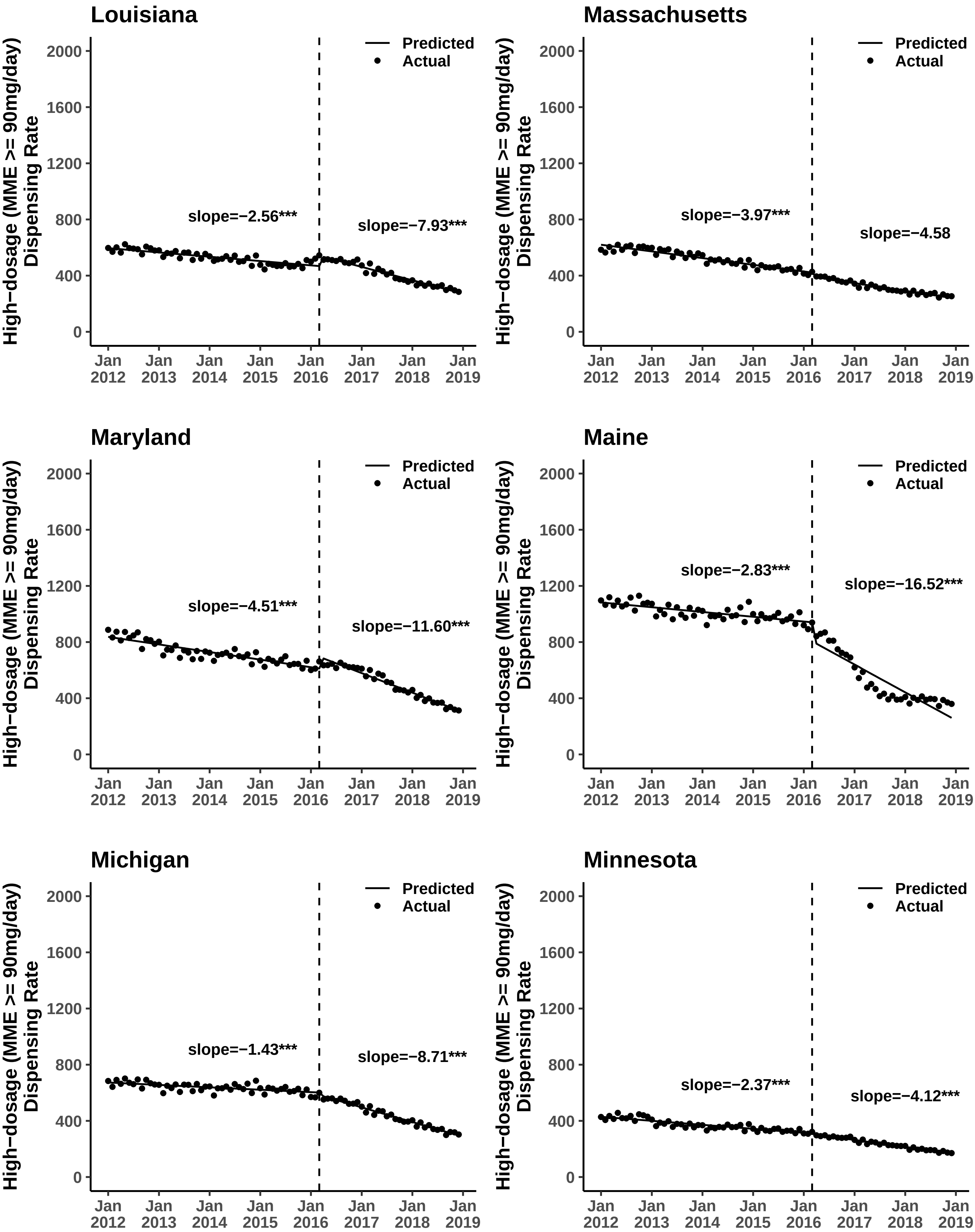

© 2023 Lyu X et al. *JAMA Network Open*.

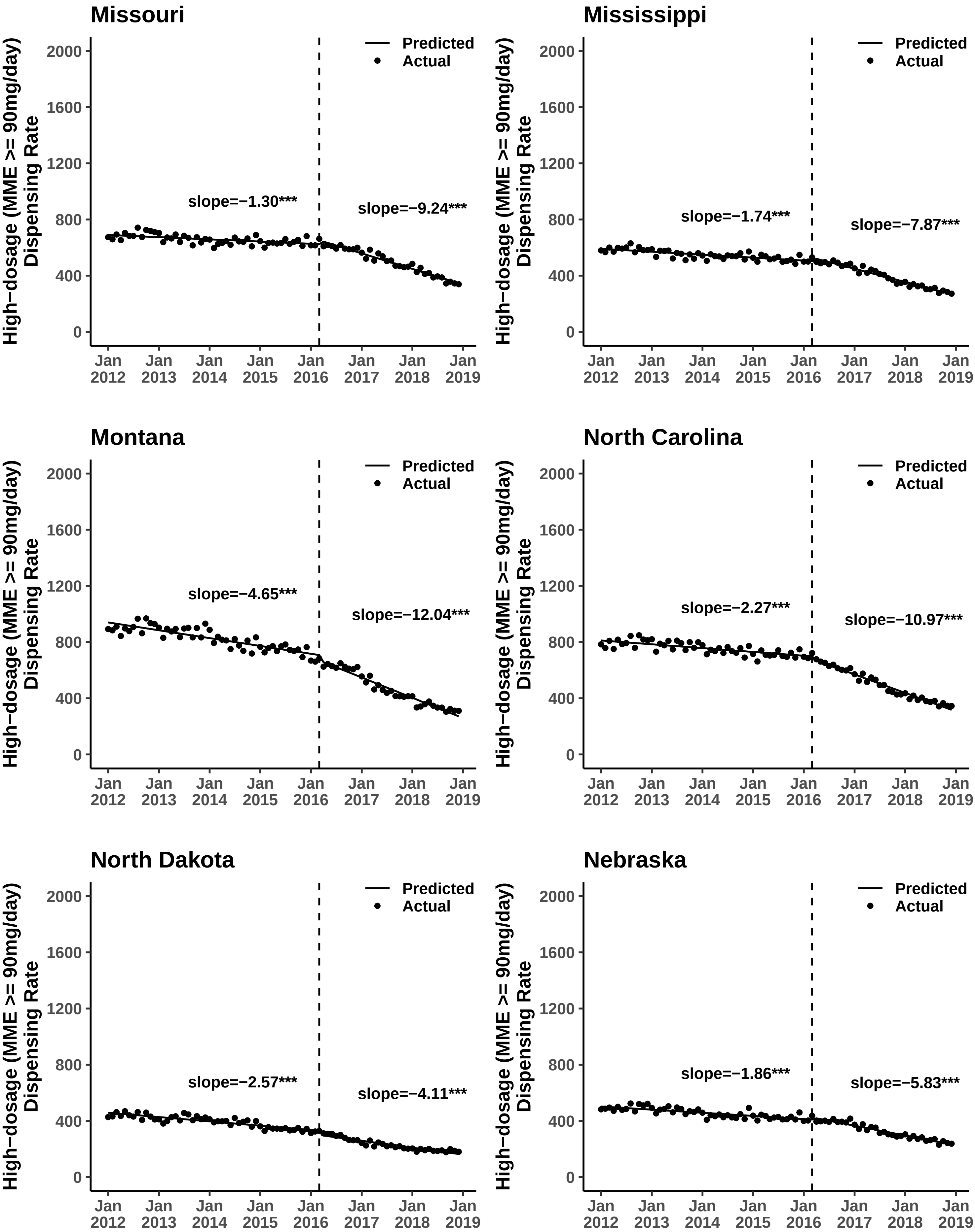

© 2023 Lyu X et al. *JAMA Network Open*.

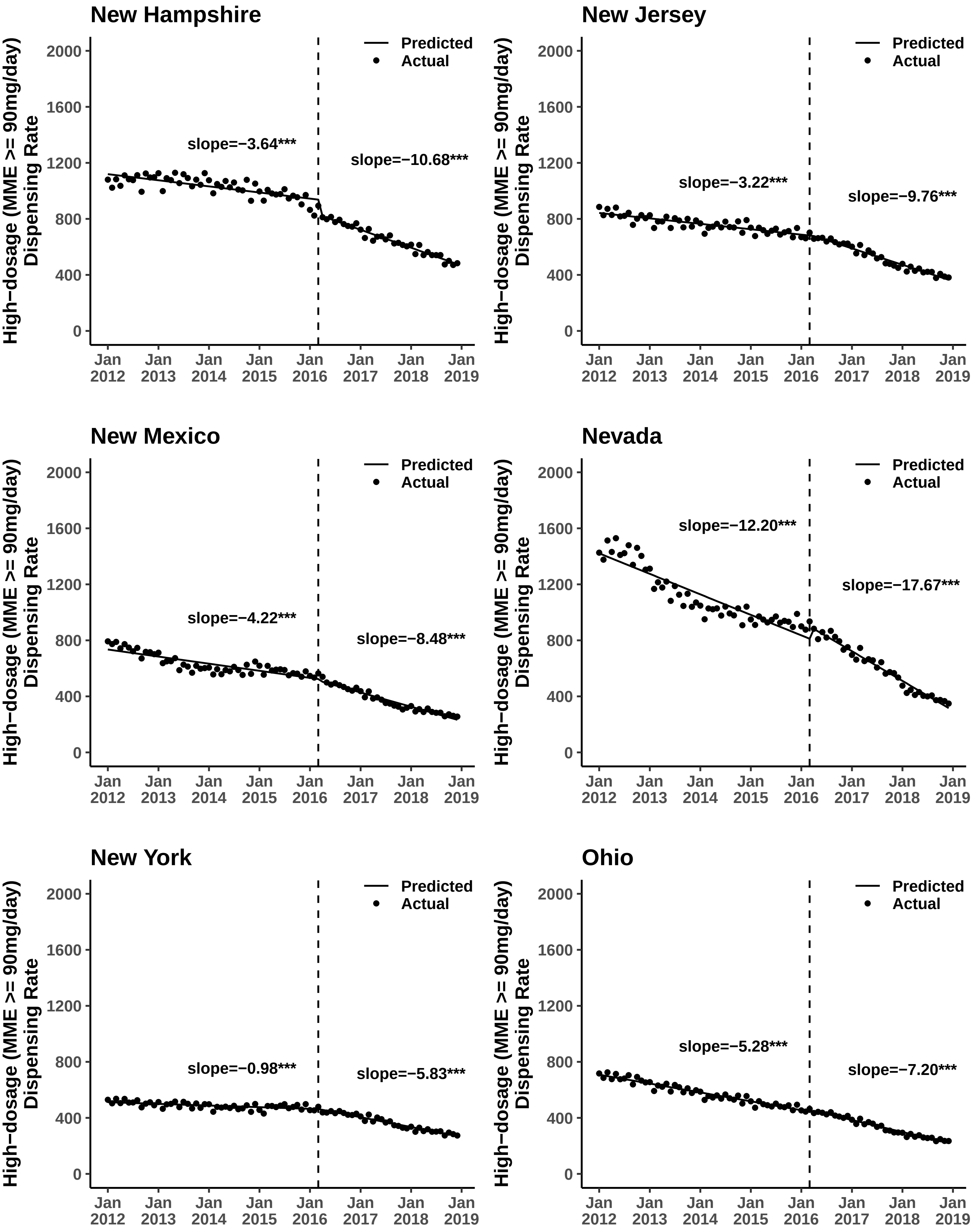

© 2023 Lyu X et al. *JAMA Network Open*.

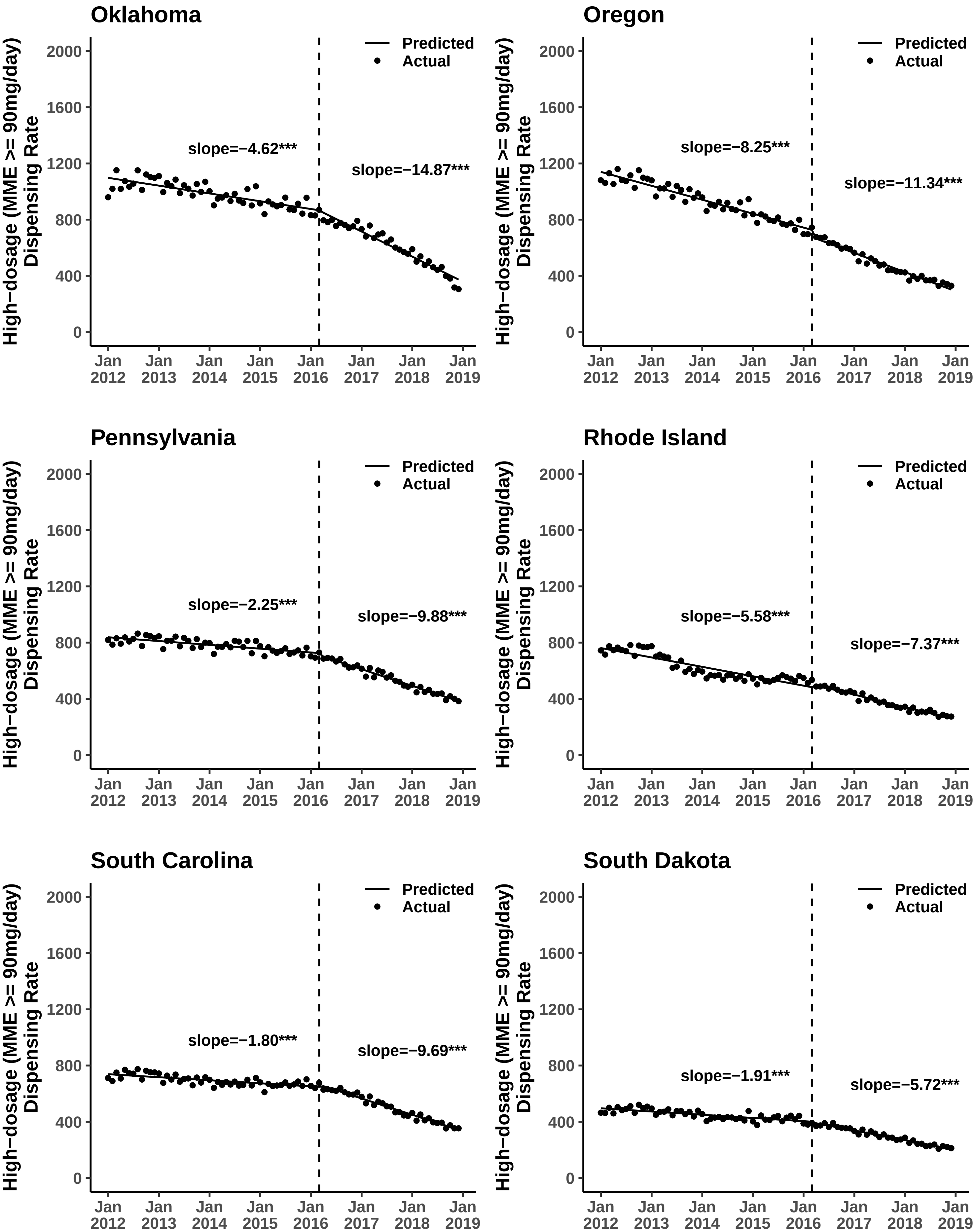

© 2023 Lyu X et al. *JAMA Network Open*.

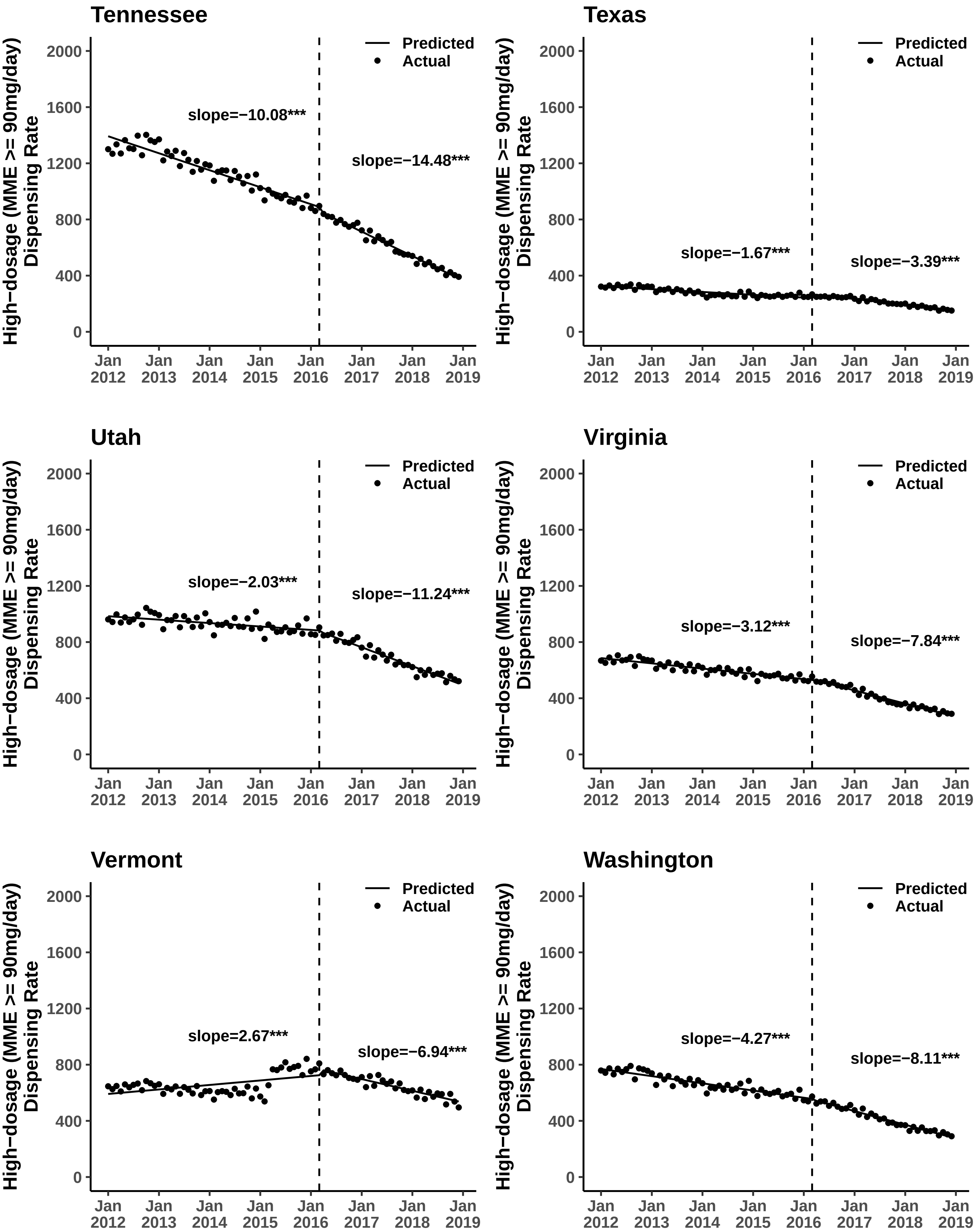

© 2023 Lyu X et al. *JAMA Network Open*.

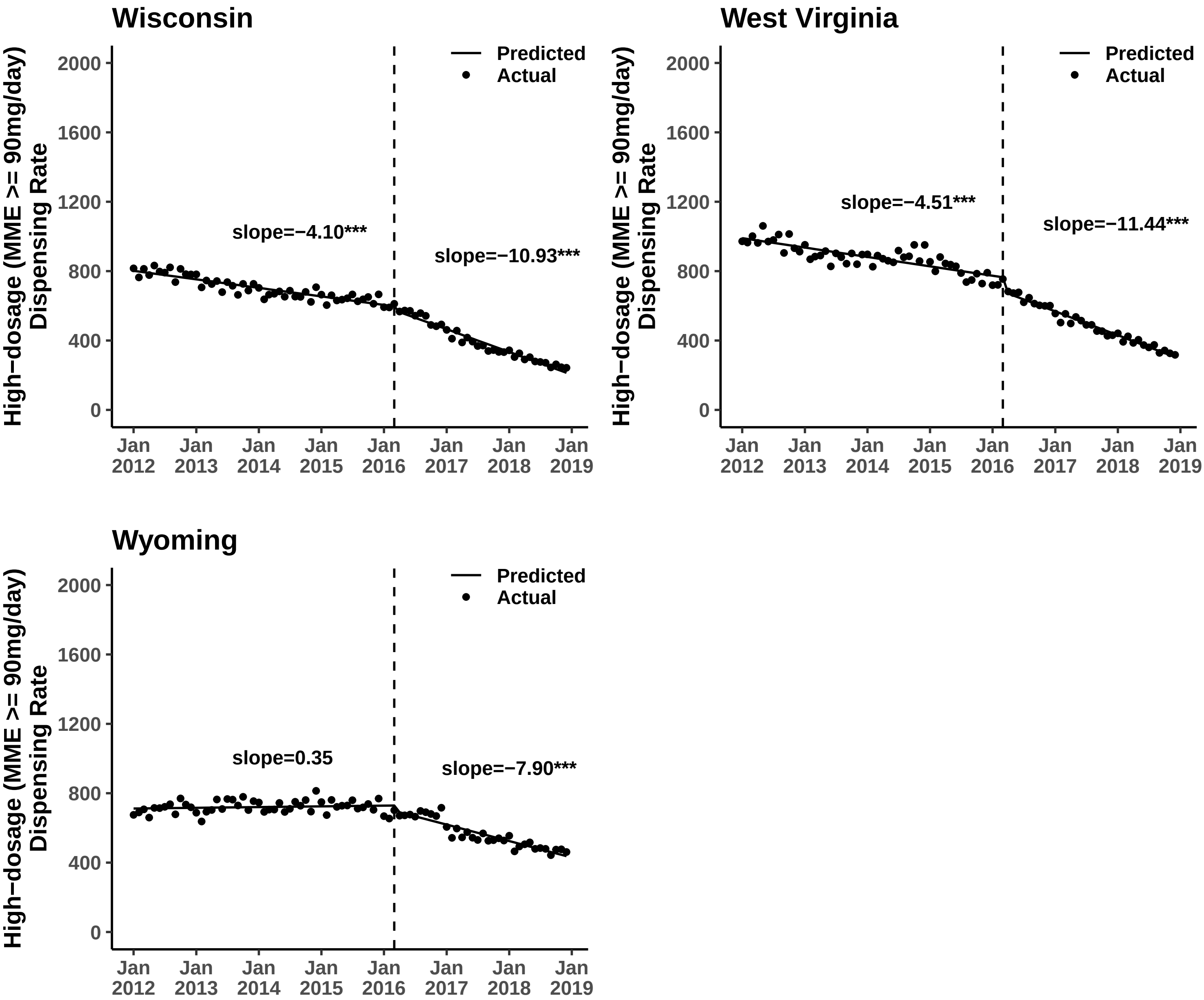

© 2023 Lyu X et al. *JAMA Network Open*.

eFigure 4. Trends in the in per-capita MME dispensing from 2012 through 2018, with linear regression lines showing the pre- and post-Guideline slopes superimposed

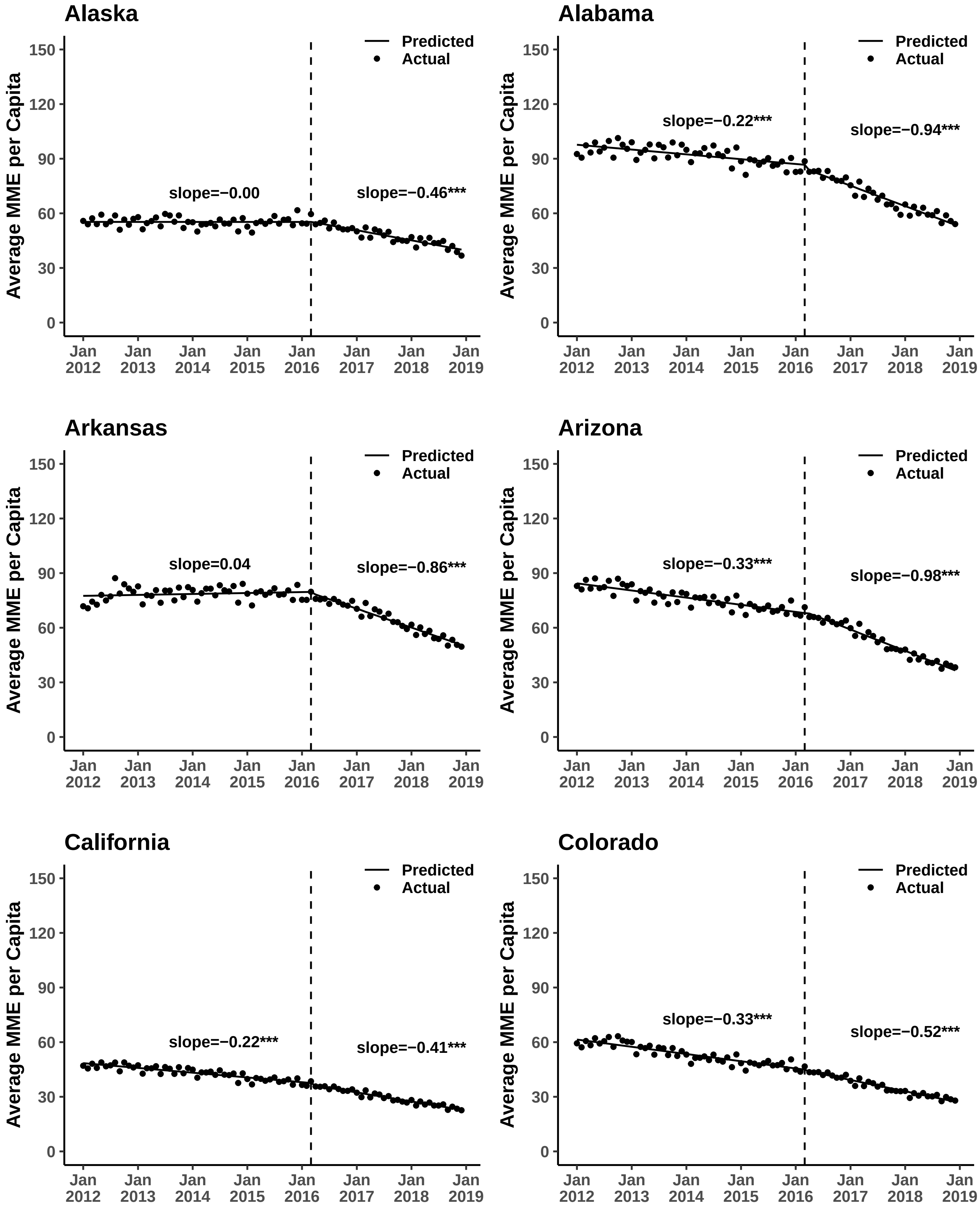

© 2023 Lyu X et al. *JAMA Network Open*.

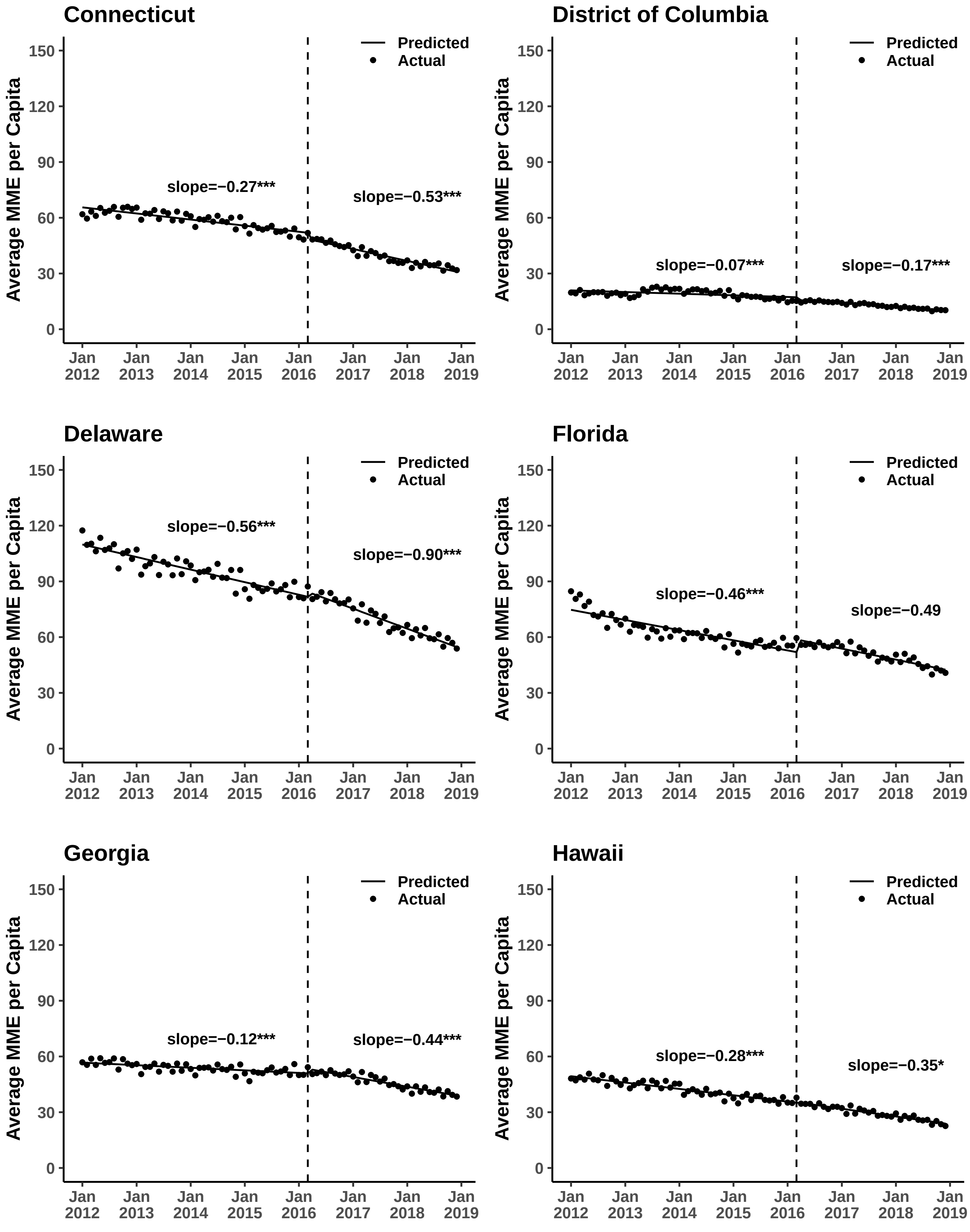

© 2023 Lyu X et al. *JAMA Network Open*.

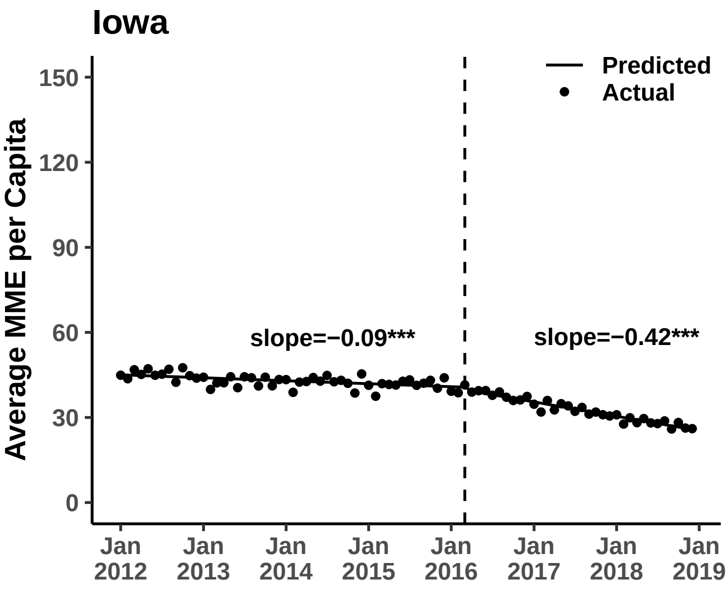

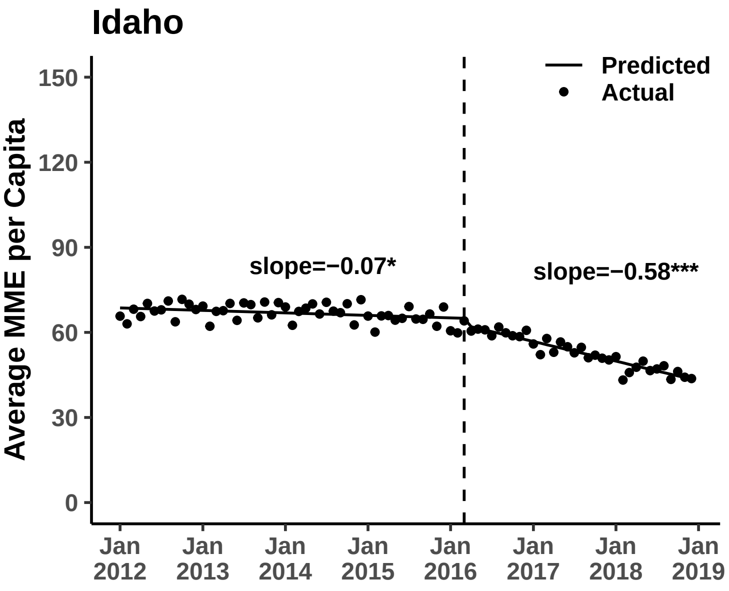

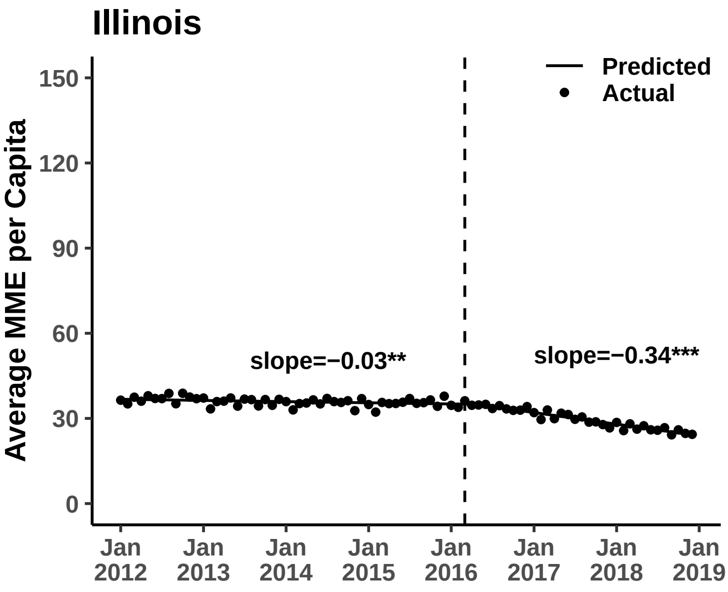

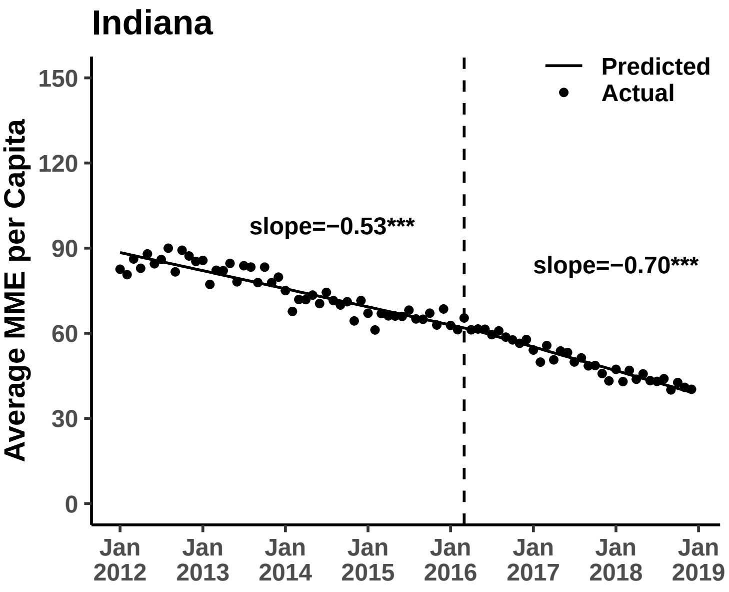

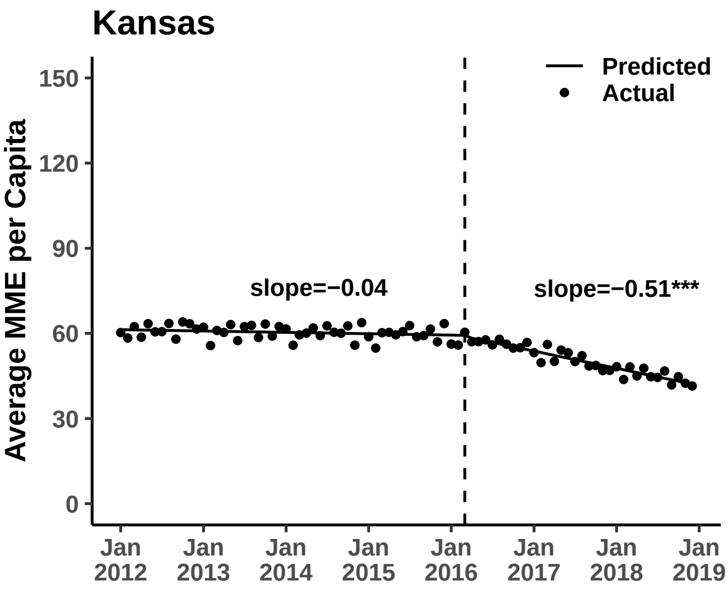

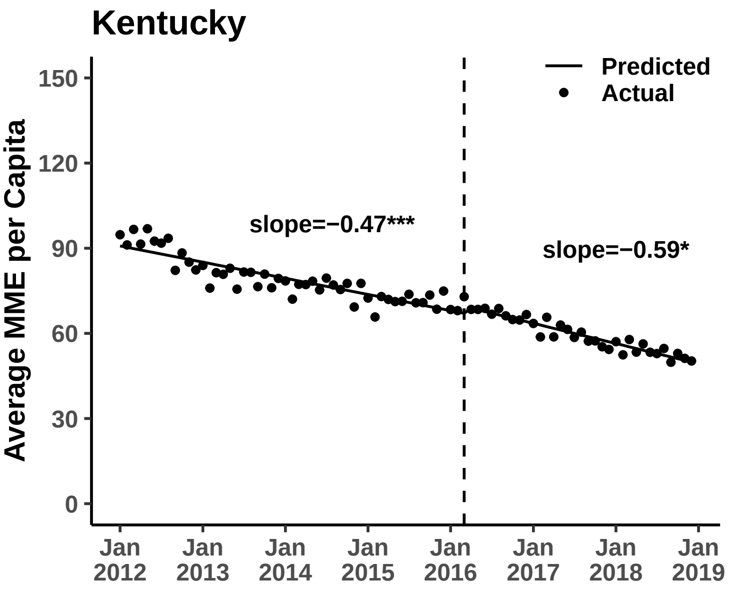

© 2023 Lyu X et al. *JAMA Network Open*.

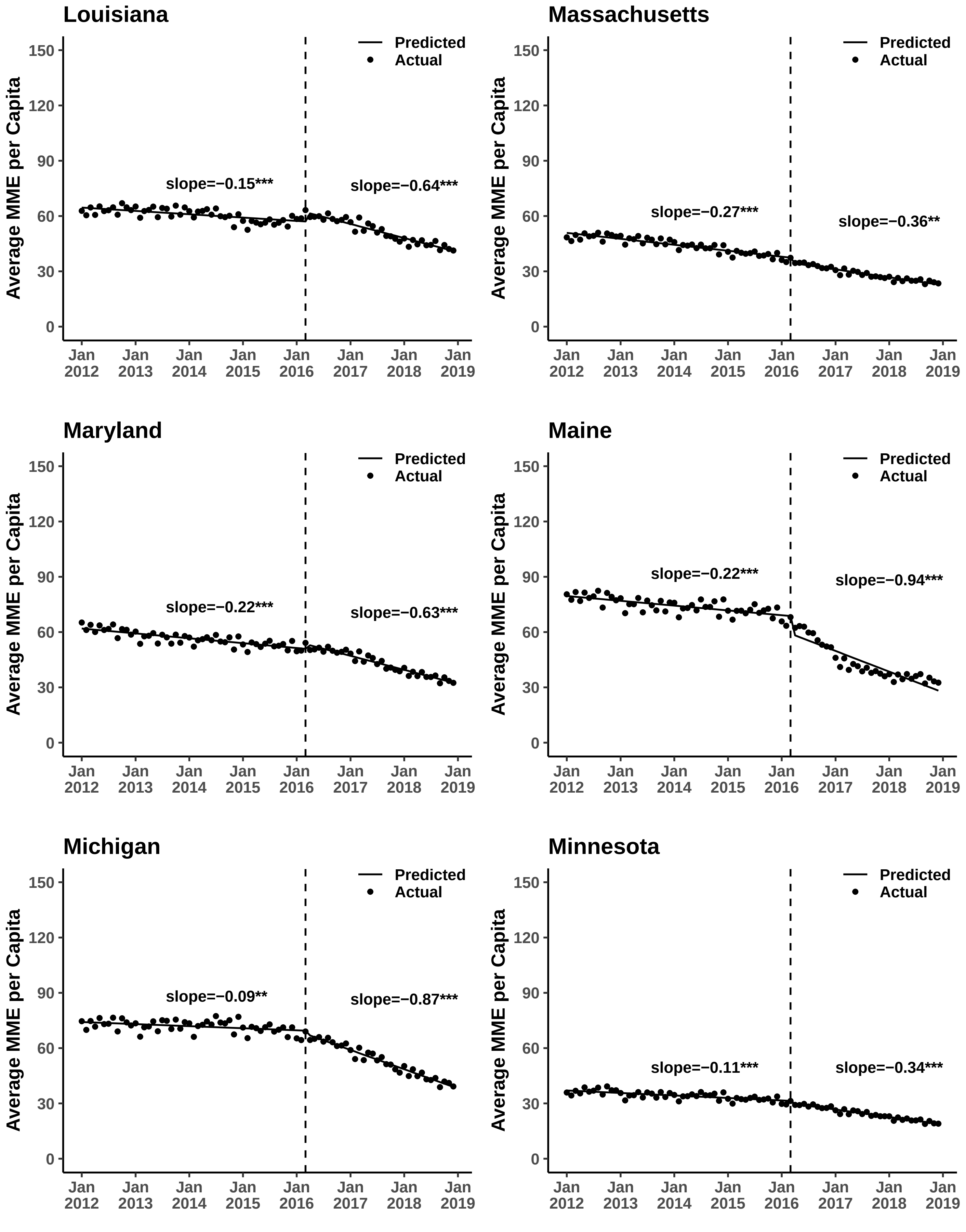

© 2023 Lyu X et al. *JAMA Network Open*.

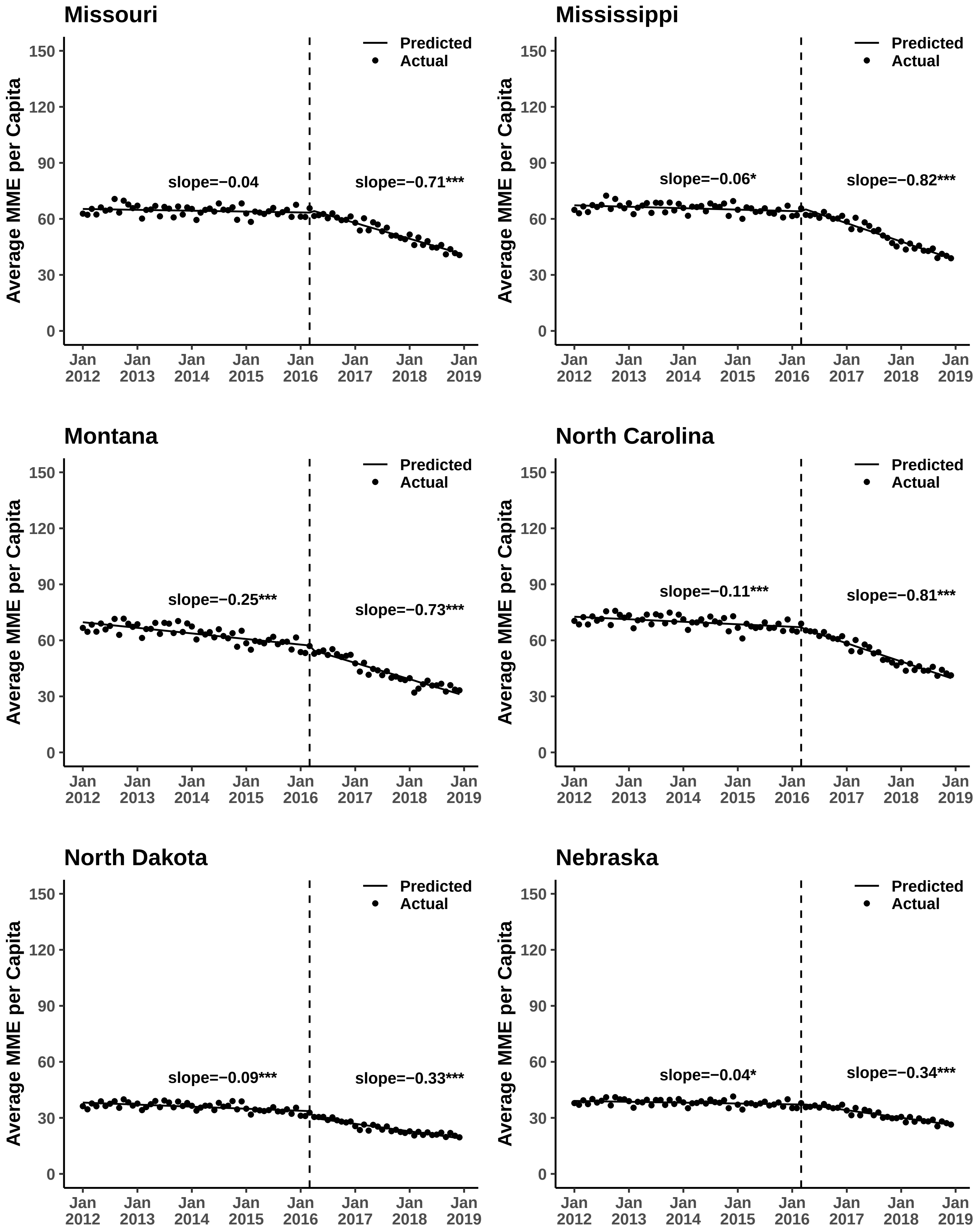

© 2023 Lyu X et al. *JAMA Network Open*.

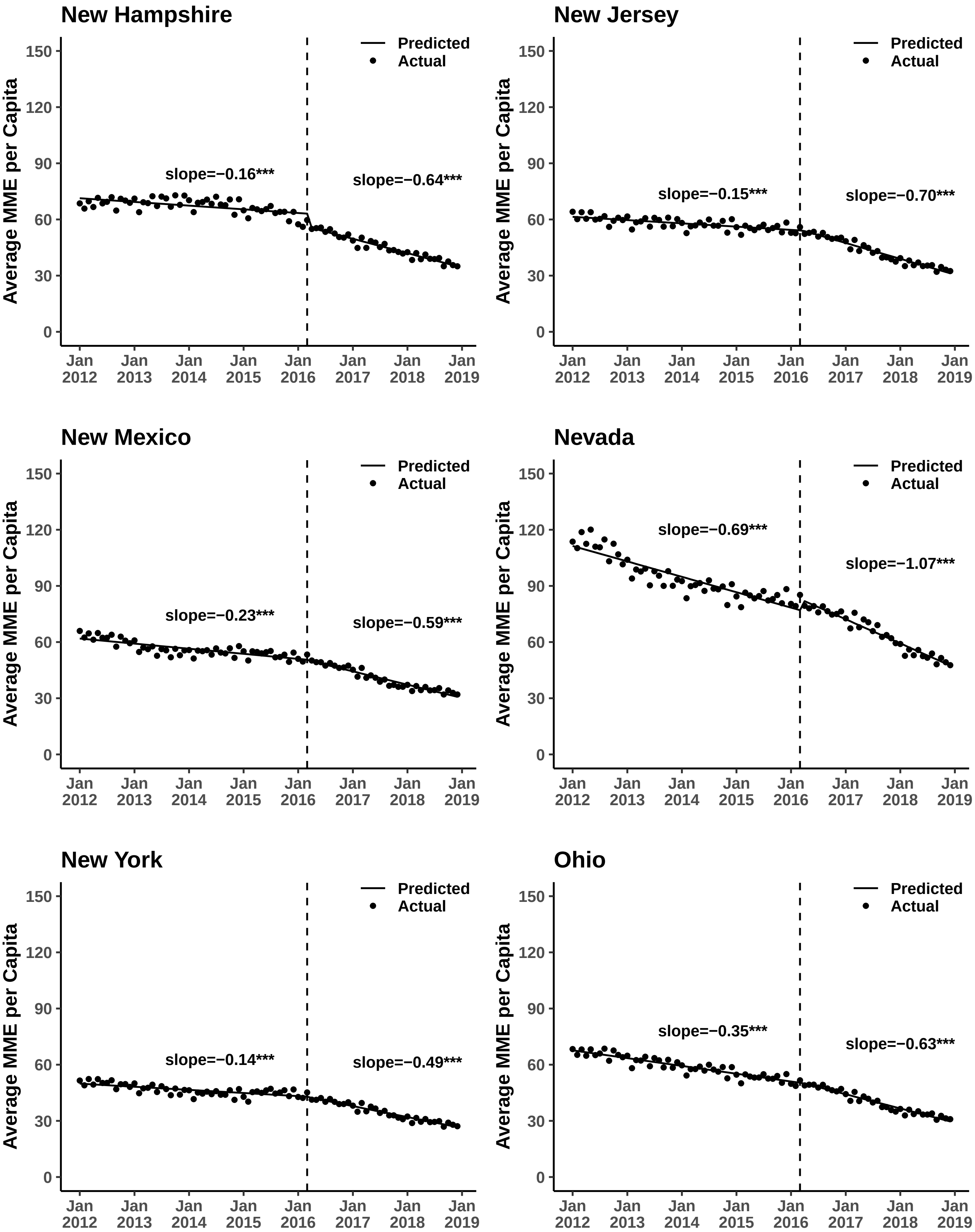

© 2023 Lyu X et al. *JAMA Network Open*.

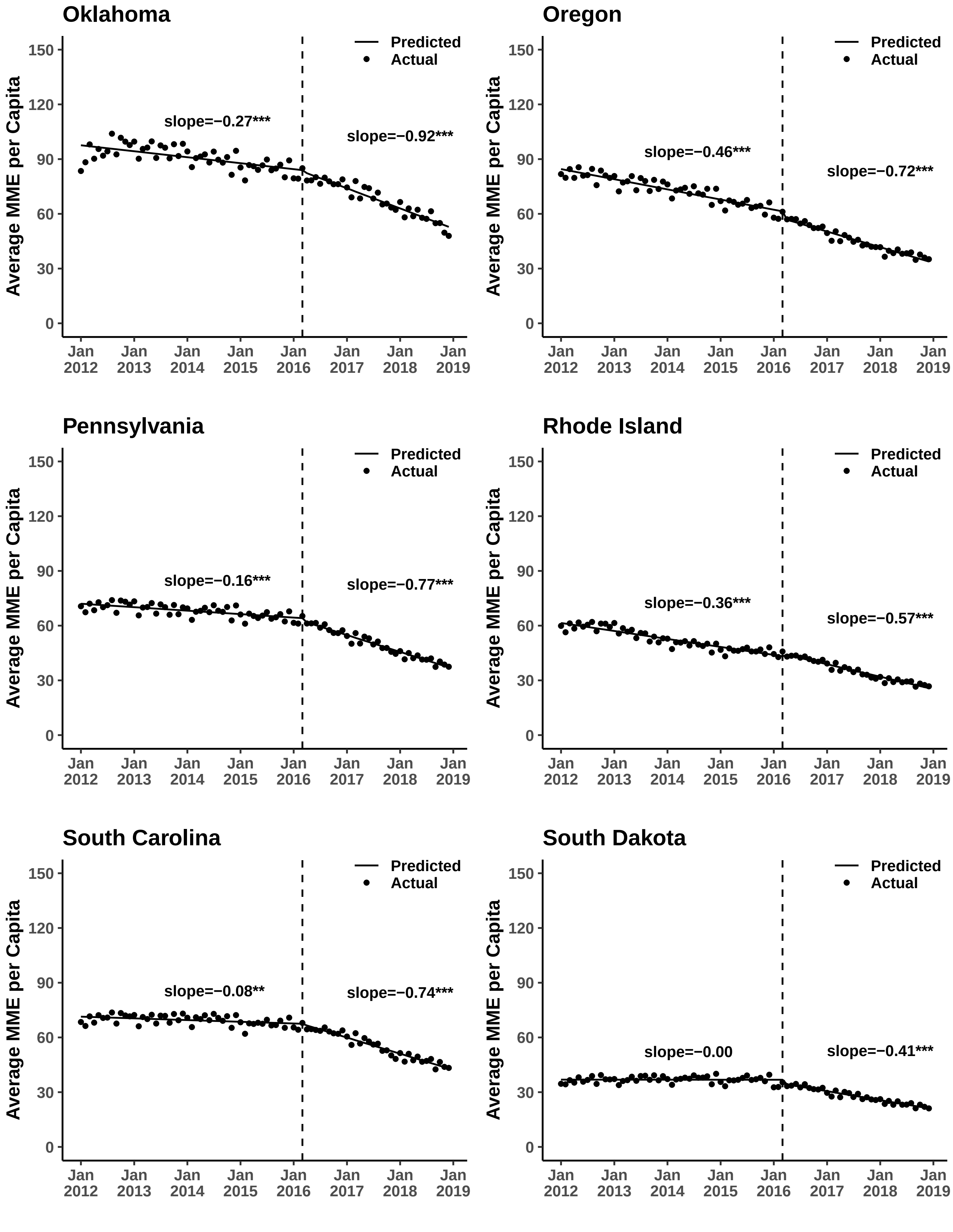

© 2023 Lyu X et al. *JAMA Network Open*.

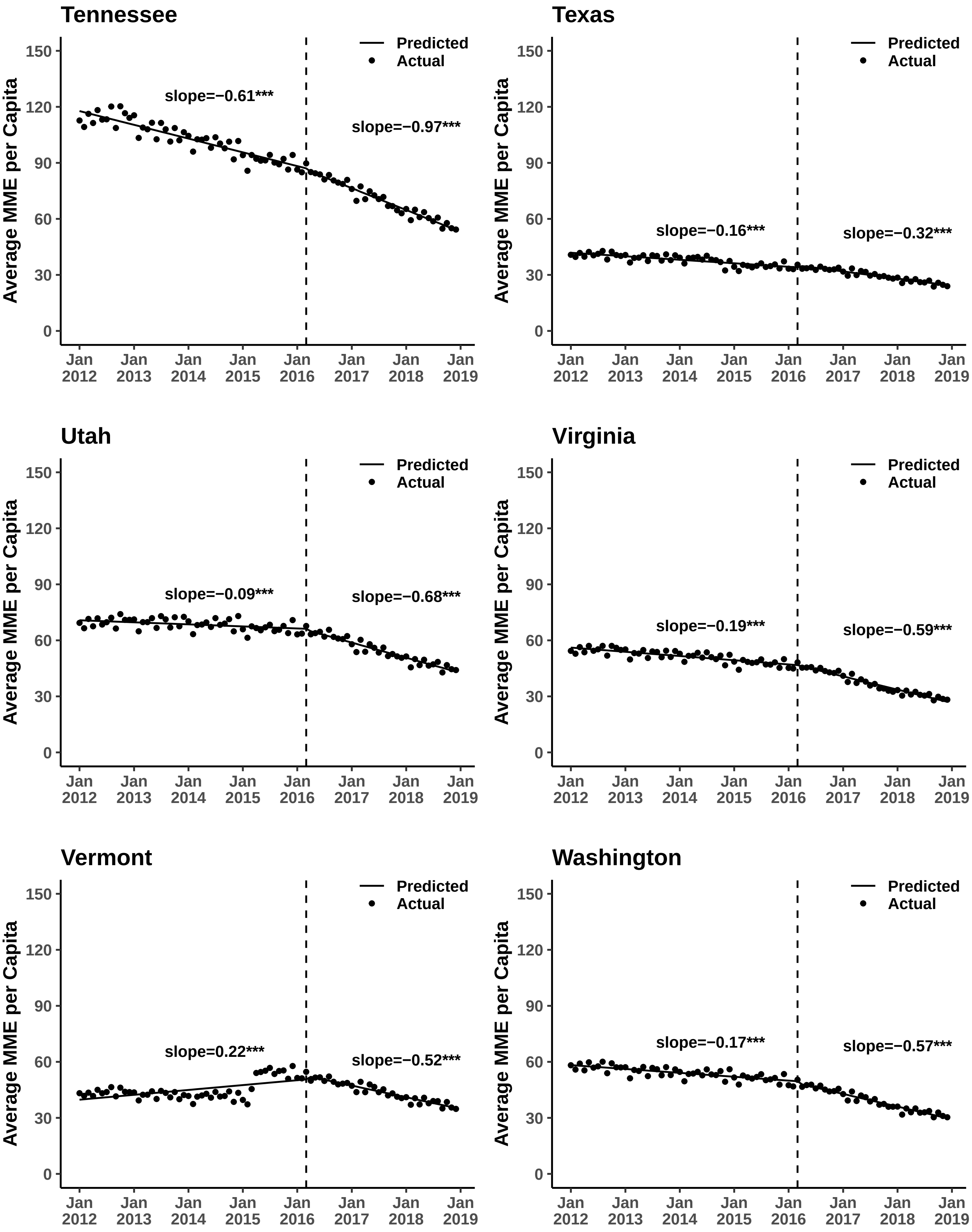

© 2023 Lyu X et al. *JAMA Network Open*.

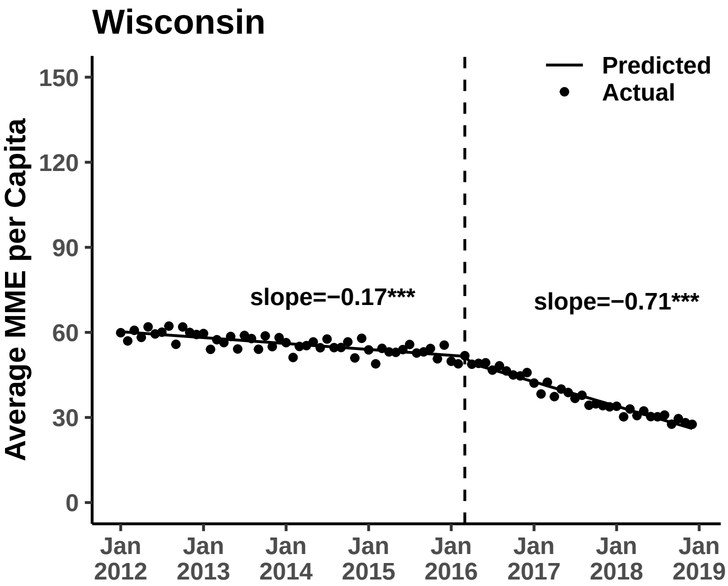

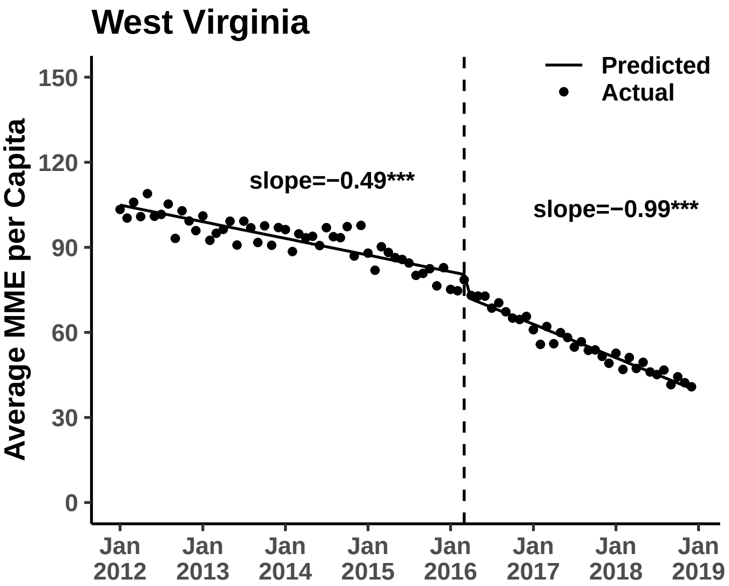

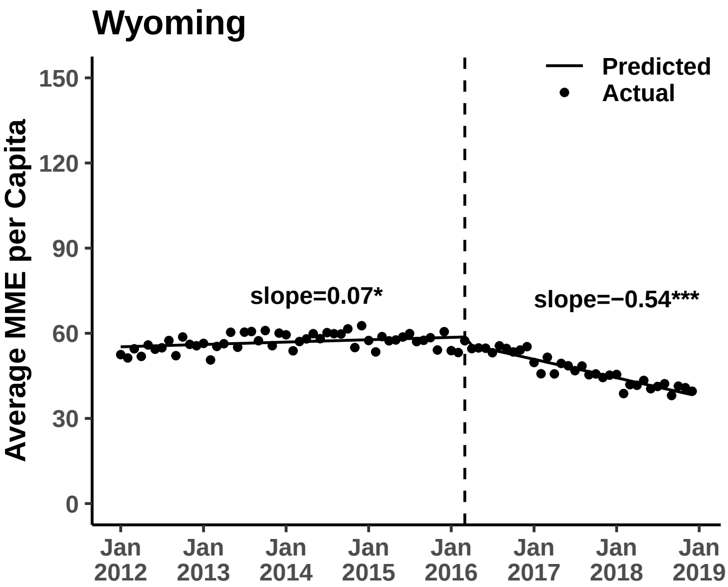

© 2023 Lyu X et al. *JAMA Network Open*.

eFigure 5. Association between baseline dispensing levels and the slope change (pre- vs. post-Guideline) at the state-level for each of the four primary outcomes

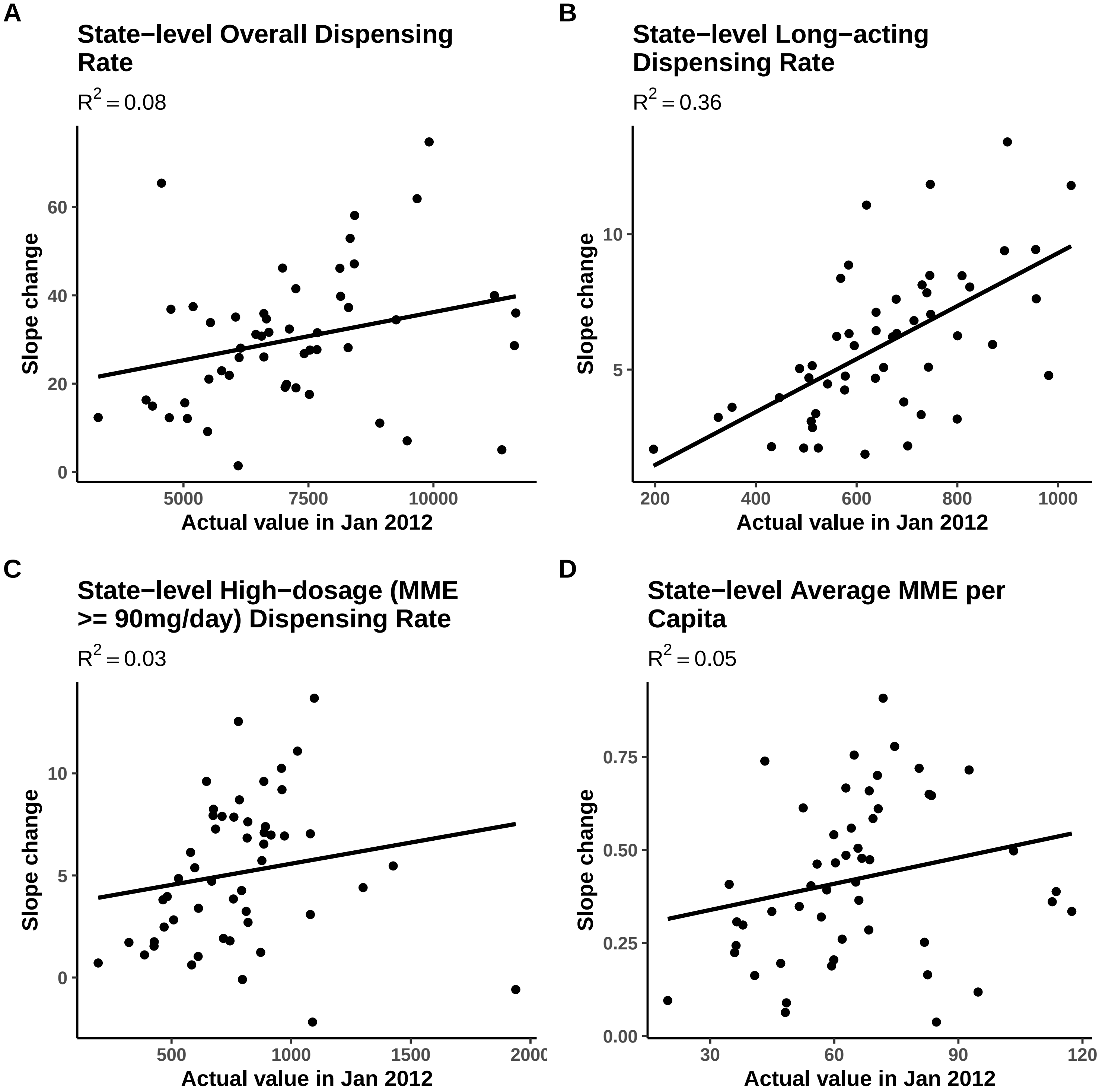

© 2023 Lyu X et al. *JAMA Network Open*.
