# Supplementary material for: State-to-State Variation in Opioid Dispensing Changes Following the Release of the 2016 CDC Guideline for Prescribing Opioids for Chronic Pain
Source: JAMA Netw Open. 2023 Sep 11;6(9):e2332507. doi: 10.1001/jamanetworkopen.2023.32507 (PMC10495870; doi:10.1001/jamanetworkopen.2023.32507)
Supplement: Supplement 2. — Data Sharing Statement [file jamanetwopen-e2332507-s002.pdf]

Data Sharing Statement

Lyu. State-to-State Variation in Opioid Dispensing Changes Following the Release of the 2016 CDC Guideline for Prescribing Opioids for Chronic Pain. *JAMA Netw Open*. Published September 06, 2023. doi:10.1001/jamanetworkopen.2023.32507

Data
Data available: No

Additional Information
Explanation for why data not available: We are unable to publicly share the IQVIA data
